# Supplementary material for: Cell surface protein aggregation triggers endocytosis to maintain plasma membrane proteostasis
Source: Nat Commun. 2023 Feb 28;14:947. doi: 10.1038/s41467-023-36496-y (PMC9974993; doi:10.1038/s41467-023-36496-y)
Supplement: Supplementary file 1 — Supplementary Information [file 41467_2023_36496_MOESM1_ESM.pdf]

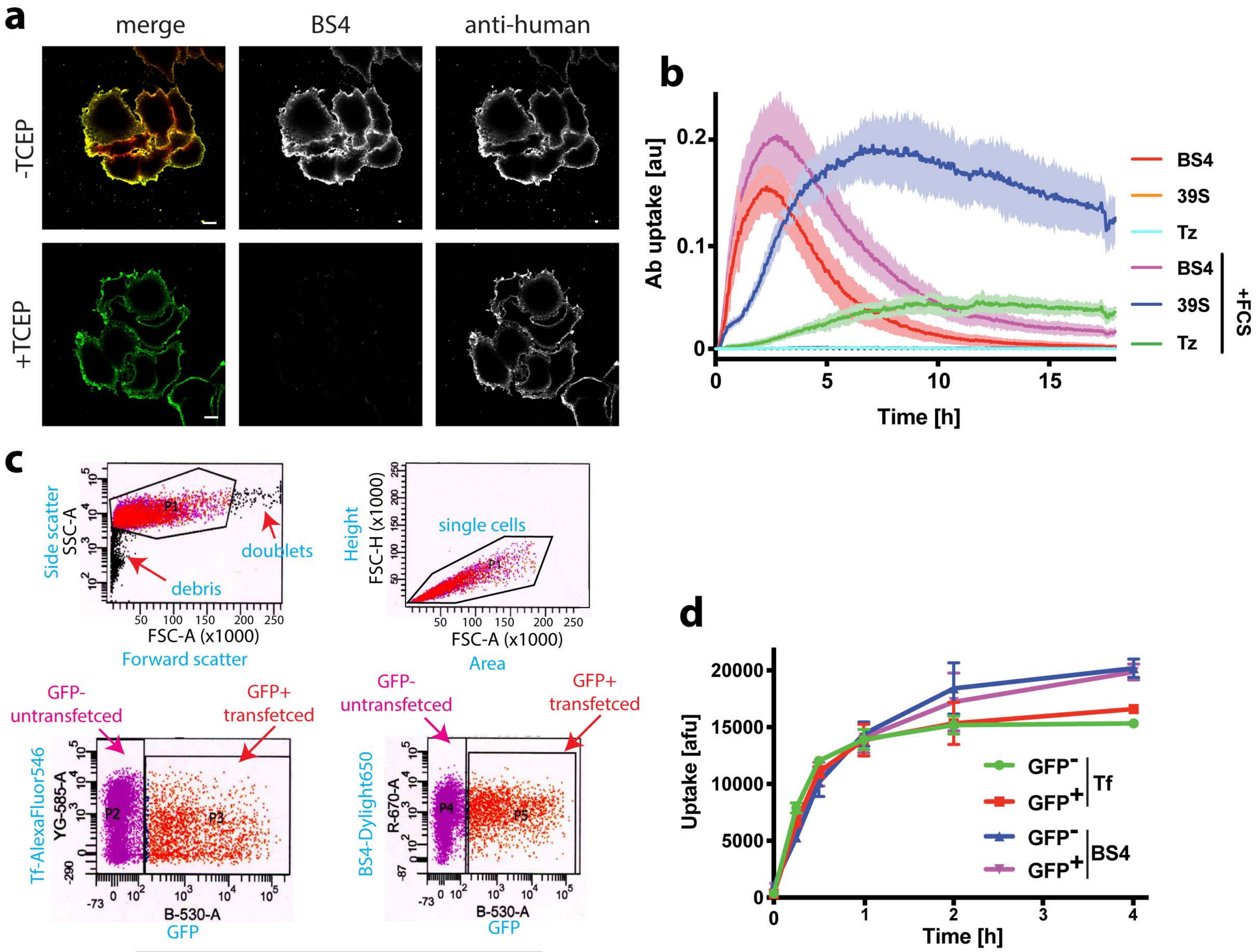

Population	Event	% Parent	B-530-A Median (GFP)	YG-585-A Median (TF-546)	R-670-A Median (BS4-Dy650)
All Events	11372	na	21	610	810
P1	10063	88.5	22	801	935
P2	8066	80.2	15	1125	900
P3	1986	19.7	892	265	1106
P4	8097	80.5	25	1117	900
P5	1892	18.8	984	262	1108

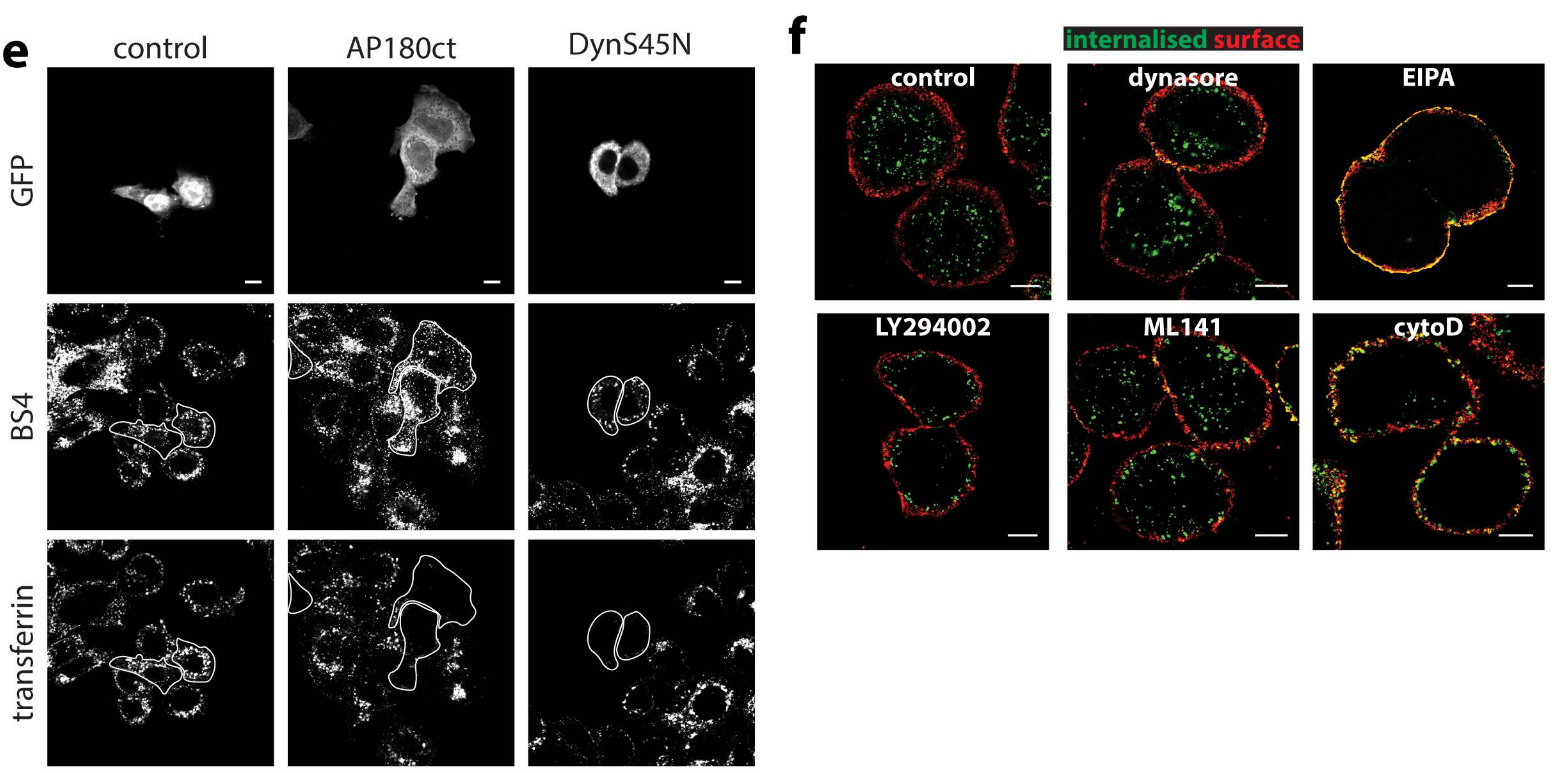

Sup Figure 1

Supplementary figure 1: Characterization of BS4 uptake. (a) Quenching of extracellular dylight-650 BS4 antibody by TCEP. Labelled BS4 was bound to SkBr3 cell surface on ice and after fixation stained with anti-human Alexa-488. Cells were imaged $\pm$ TCEP in the mounting medium. **(b)** Endocytosis of pHrodo-labelled monotopic antibodies Tz and 39S and biparatopic BS4 in the absence and presence of serum (FCS) was quantified using an Incucyte live cell imager. Data are presented as means $\pm$SEM, n= 2 independent experiments **(c)** Gating strategy to quantify transferrin and BS4 uptake in transfected SkBr3 cells. Cell were transfected with dominant negative AP180ct N-terminally GFP tagged expression plasmid were incubated with dylight650 labelled BS4 and AlexaFluor546-transferrin for 15 min and analysed by flow-cytometry as follows: Initial gating was area vs height to eliminate doublets and gating on forward and side scatter to exclude debris. Cells selected by this strategy were further gated on GFP if transfected, providing intrinsic un-transfected controls for each cell population. Transferrin and BS4 uptake was measured in the red and far-red channel, respectively both in un-transfected (GFP-) and transfected (GFP+) cells. **(d)** SkBr3 cells transfected with control (GFP only) plasmid were incubated with dylight650 labelled BS4 and AlexaFluor546-transferrin for indicated times over a period of 4 h and analysed by flow-cytometry. Endocytosis of transferrin and BS4 in cells from the same well expressing or not GFP (GFP^+, GFP^-), (means $\pm$ SD, n=4 independent experiments). **(e)** SkBr3 cells transfected with control (GFP only) and dominant negative AP180ct and DynaminS45N N-terminally GFP tagged expression constructs were incubated with dylight650 labelled BS4 and AlexaFluor546-transferrin for 30 min and analysed by confocal microscopy. Transfected cells (GFP channel) are outlined in the images displaying BS4 and transferrin. **(f)** Endocytosis of Dylight488 labelled BS4in SkBr3 cells in the presence of 80 µM dynasore, 100 µM 5-(N-ethyl-N-isopropyl)amiloride (EIPA), 100 µM LY294002, 10 µM ML141 and 0.5 µM CytochalasinD

(CytoD) for 1h. After fixation surface BS4 was counterstained with anti-human Alexa568 antibody and imaged by confocal microscopy. Representative images for each condition are shown.

Scale bars: 10 µm. Source data are provided as a Source Data file.

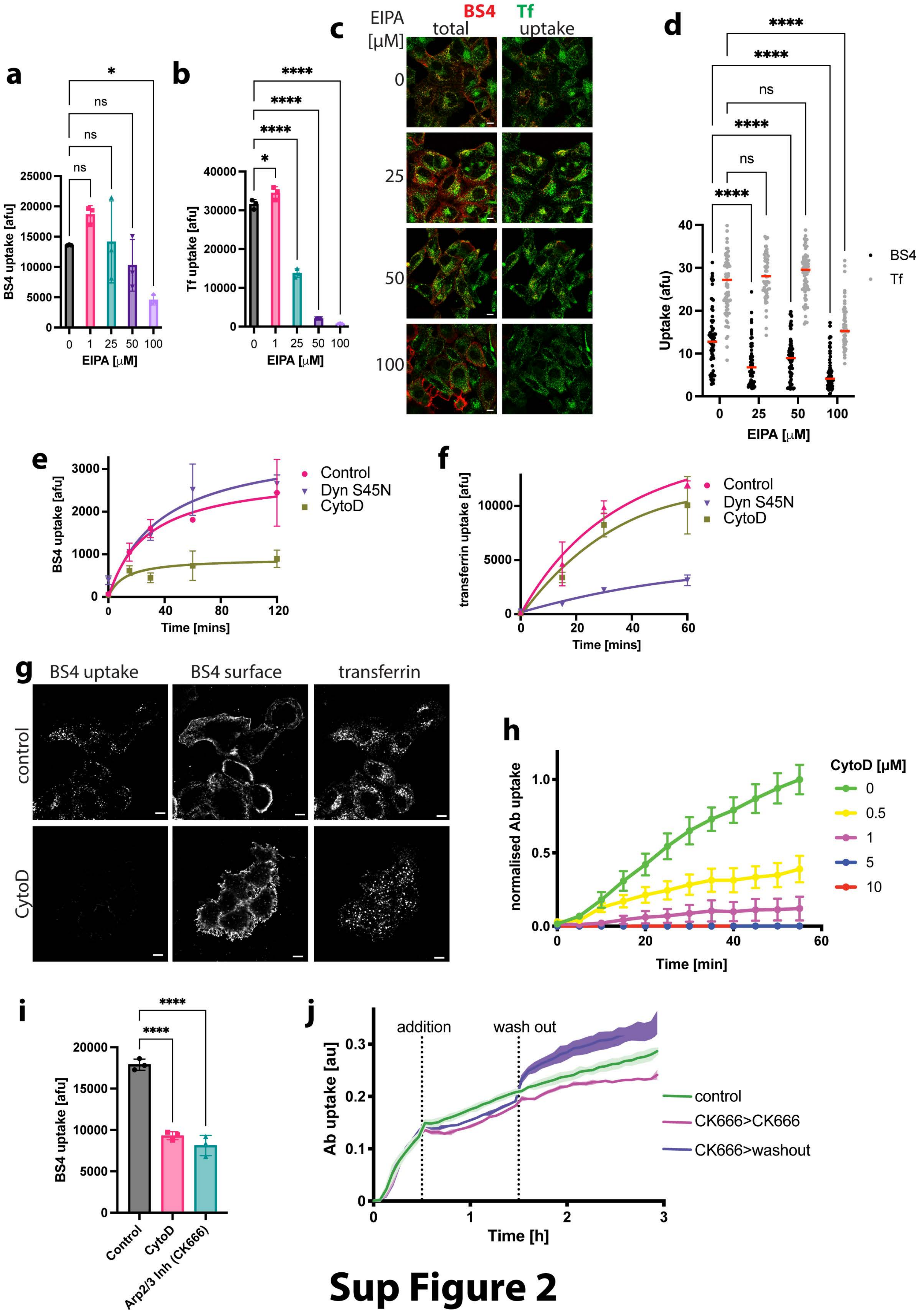

a
BS4 uptake [afu]
EIPA [µM]
ns
*
b
Tf uptake [afu]
EIPA [µM]
****
c
EIPA [µM]
BS4 total
Tf uptake
0
25
50
100
d
Uptake (afu)
EIPA [µM]
BS4
Tf
****
ns
e
BS4 uptake [afu]
Time [mins]
Control
Dyn S45N
CytoD
f
transferrin uptake [afu]
Time [mins]
Control
Dyn S45N
CytoD
g
BS4 uptake
BS4 surface
transferrin
control
CytoD
h
normalised Ab uptake
Time [min]
CytoD [µM]
0
0.5
1
5
10
i
BS4 uptake [afu]
Control
CytoD
Arp2/3 Inh (CK666)
j
Ab uptake [au]
Time [h]
addition
wash out
control
CK666>CK666
CK666>washout

Sup Figure 2

Supplementary figure 2: Inhibition of BS4 uptake by chemical inhibitors. Endocytosis of BS4 **(a)** and transferrin **(b)** in presence of increasing concentrations of 5-(N-ethyl-N-isopropyl)amiloride (EIPA). After 30 min of uptake SkBr3 cells were processed and analysed by flow-cytometry (means ± SD, n=3 independent experiments, ns (non-significant) p>0.05, * p<0.05,**** p<0.0001, one-way ANOVA with Dunnett's multiple comparison test). **(c)** Confocal microscopy images of BS4 (red) and transferrin (green) uptake into Hela cells ectopically expressing full-length HER2 treated with increasing concentrations of EIPA. After fixation surface bound BS4-dylight 650 was counterstained using Alexa568 conjugated anti-human IgG. Surface subtracted images highlighting endocytosed BS4 are shown on the right of each corresponding image. Results of BS4 and Tf uptake in presence of increasing concentrations of EIPA are quantified in **(d)**. Each dot represents the measurement from an individual cell, red lines indicate medians (n>50, ns (non-significant),**** p<0.0001; two-way ANOVA with Dunnett's multiple comparison test).

Endocytosis of BS4 **(e)** and transferrin **(f)** in SkBr3 cells expressing the dominant-negative dynamin 1 S45N mutant or treated with 10 µM CytoD. After indicated times cells were processed and analysed by flow-cytometry (means ± SD, n=3 independent experiments).

(g) Confocal microscopy images of BS4 and transferrin uptake into cells treated with 10 µM CytoD. After fixation surface bound BS4-dylight 650 was quenched and counterstained using Alexa488 conjugated anti-human IgG. **(h)** Cells treated with increasing concentrations of CytochalasinD (CytoD) were incubated with pHrodo-labelled BS4 and uptake was quantified using an Incucyte live cell imager (means ± SD, n=3 independent experiments). **(i)** Uptake of BS4 in SkBr3 cells treated with 10 µM CytoD or 300 µM CK666. After 30 min of uptake SkBr3 cells were processed and analysed by flow-cytometry (means ± SD, n=3 independent experiments ,**** p<0.0001, one-way ANOVA with Dunnett's multiple comparison test). **(j)**

Cells endocytosing pHrodo-labelled BS4 were treated with 300 µM CK666, which was subsequently washed out as indicated, (means $\pm$ SEM, n=2 independent experiments).

Scale bars: 10 µm. Source data are provided as a Source Data file.

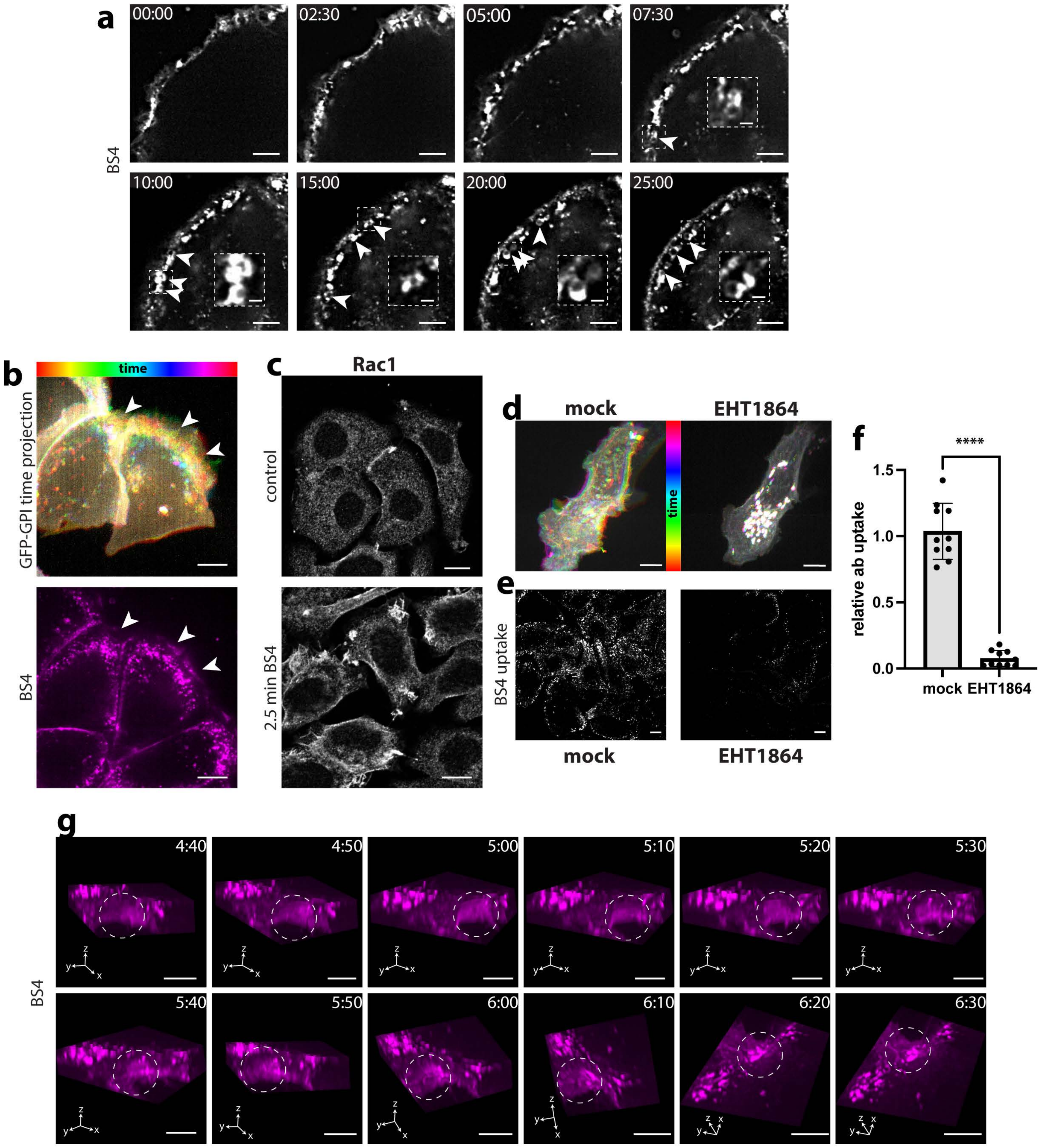
a
00:00
02:30
05:00
07:30
10:00
15:00
20:00
25:00
BS4
b
time
GFP-GPI time projection
BS4
c
Rac1
control
2.5 min BS4
d
mock
EHT1864
time
e
BS4 uptake
mock
EHT1864
f
****
relative ab uptake
1.5
1.0
0.5
0.0
mock
EHT1864
g
4:40
4:50
5:00
5:10
5:20
5:30
5:40
5:50
6:00
6:10
6:20
6:30
BS4

Sup Figure 3

Supplementary figure 3: Rac1-dependent plasma membrane rearrangements during BS4 uptake resemble macro-endocytosis. (a) BS4 marks large endocytic vesicles. Snapshots of BS4-dylight650 endocytosis in SkBr3 cells at indicated times after antibody addition (see also movie 1). Arrowheads indicate plasma membrane proximal vesicles containing BS4. Dashed boxes show vesicles at higher magnification. **(b)** BS4 preferentially endocytoses in regions exhibiting high plasma membrane motility. GFP-GPI expressing cells were imaged during BS4 uptake. Maximum intensity time projections of the GFP-GPI channel over the imaging period are displayed, with time frames color-coded as indicated. Arrowheads highlight highly motile regions of the plasma membrane, which coincide with areas of BS4 endocytosis. **(c)** Accumulation of Rac1 at regions of the plasma membrane harbouring BS4 aggregates (see Fig. 2e). SKBR3 cells before and after 2.5 min incubation with BS4 were immune-stained for endogenous Rac1 protein and imaged by confocal microscopy. **(d)** Plasma membrane lamellipodia movement is blocked by Rac1 inhibition. GFP-GPI expressing cells were imaged prior to and after incubation with Rac1 inhibitor EHT1864 (see also movie 5). Maximum intensity time projections over the imaging period are displayed, with time frames color-coded as indicated. **(e)** Rac1 inhibition blocks BS4 endocytosis. Dylight labelled BS4 was added to cells treated with EHT1864 and uptake was allowed for 20 min. Surface bound antibody was quenched. **(f)** Quantification of the effect of EHT1864 on BS4 uptake (means $\pm$ SD, n=10 cells from 3 independent experiments; ****p<0.0001; two-tailed unpaired Student's *t*-test). **(g)** 3D views of the BS4 channel only corresponding to frames from Figure 2 c are displayed. Dashed circles indicate the position of the macropinocytic cup.

Scale bars 5 µm (a,f) insets in (a) 1 µm, 10 µm (b,c, d,e). Source data are provided as a Source Data file.

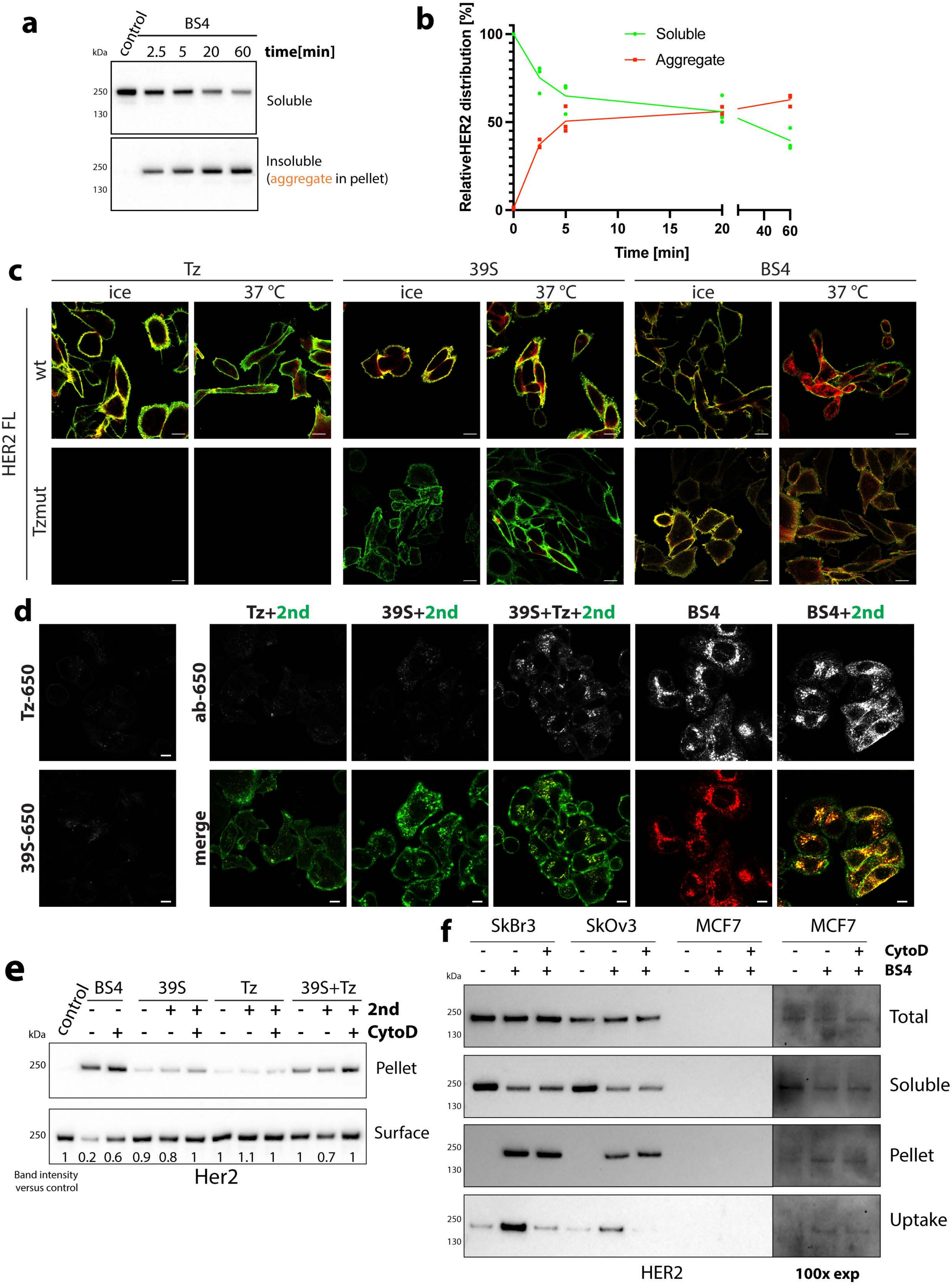

a
control
BS4
kDa
2.5 5 20 60
time[min]
250
130
Soluble
Insoluble
(aggregate in pellet)
b
RelativeHER2 distribution [%]
Soluble
Aggregate
Time [min]
c
Tz
39S
BS4
ice
37 °C
HER2 FL
wt
Tzmut
d
Tz+2nd
39S+2nd
39S+Tz+2nd
BS4
BS4+2nd
Tz-650
39S-650
ab-650
merge
e
control
BS4
39S
Tz
39S+Tz
2nd
CytoD
kDa
Pellet
Surface
1 0.2 0.6 0.9 0.8 1 1 1.1 1 1 0.7 1
Band intensity versus control
Her2
f
SkBr3
SkOv3
MCF7
MCF7
CytoD
BS4
Total
Soluble
Pellet
Uptake
HER2
100x exp

Sup Figure 4

Supplementary figure 4: BS4-induced receptor aggregation triggers HER2 endocytosis (a) Time course of HER2 receptors aggregation by BS4. SkBr3 cells were incubated with BS4 for indicated times and samples lysed in low detergent buffer. Samples were spun to separate soluble (Sup) from insoluble/aggregated proteins (Pelletand assayed by immunoblot for the HER2 protein. **(b)** Time dependence of BS4-triggered aggregation and concomitant loss in the soluble fraction of HER2 receptor is quantified (means $\pm$ SD, n=3 independent experiments). **(c)** Surface binding and internalisation of dylight650-labelled antibodies Trastuzumab (Tz), 39S and BS4 in HER2 expressing CHO cells analysed by microscopy. CHO cells expressing either wt or TZ binding mutant (Tzmut) of full-length (FL) HER2 were incubated with dylighyt650-BS4 for 30 min on ice or at 37 °C. Cells were fixed, surface bound antibody stained with anti-human Alexa488 and imaged by confocal microscopy**. (d)** Cells were incubated with equal amounts of indicated antibodies, with or without a cross-linking anti-human Alexa488 antibody (2nd) for 1h. After fixation surface bound, dylight650 labelled antibody was quenched and cell sections analysed by confocal microscopy. All control conditions related to Fig 3e are shown. **(e)** Cross-linking of both monotopic antibodies phenocopies the effect of BS4 on HER2 aggregation and endocytosis. Cells were incubated for 10 min with equal amounts of indicated antibodies, with or without a cross-linking anti-human antibody (2nd) and $\pm$ CytochalasinD (CytoD). After cell surface biotinylation a fraction of sample was spun to pellet insoluble/aggregated proteins (Pellet). Protein in the remaining sample was solubilised (see materials and methods for protocol) and surface remaining biotinylated proteins concentrated on Streptavidin beads (surface). Samples were analysed by immunoblot for HER2. **(f)** Abundance of HER2 protein is a prerequisite for BS4-mediated clustering and endocytosis. Indicated cell lines were surface biotinylated and incubated with BS4 for 10 min. Samples were analysed as described in the

materials and methods section**.** A 100 fold longer exposure of HER2 blots for MCF7 cells is shown on the right.

Scale bars: 10 µm. Source data are provided as a Source Data file.

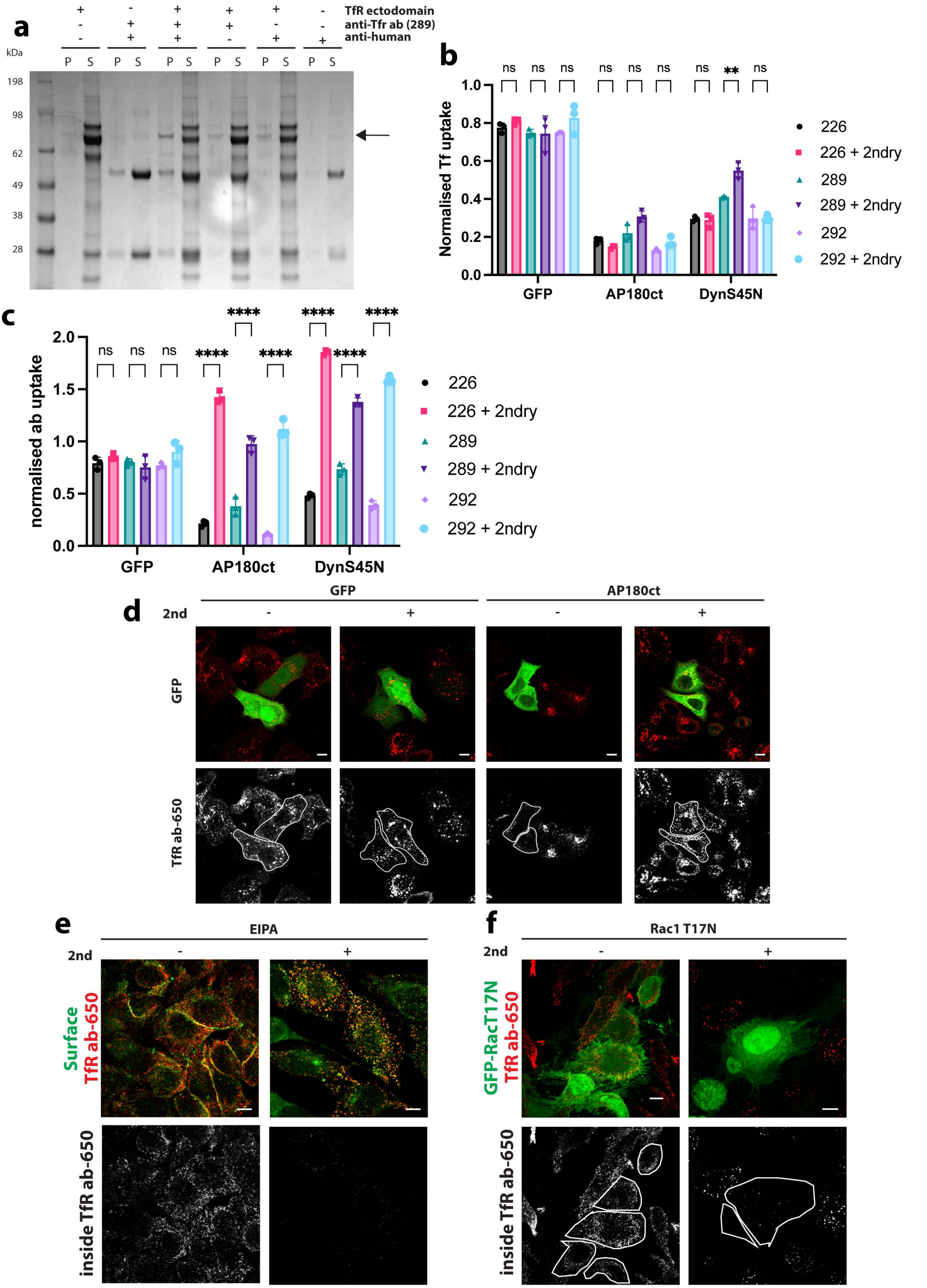

a
TfR ectodomain
anti-Tfr ab (289)
anti-human
kDa
P S
198
98
62
49
38
28
b
ns
**
Normalised Tf uptake
1.0
0.8
0.6
0.4
0.2
0.0
GFP
AP180ct
DynS45N
226
226 + 2ndry
289
289 + 2ndry
292
292 + 2ndry
c
****
normalised ab uptake
2.0
1.5
1.0
0.5
0.0
d
GFP
AP180ct
2nd
TfR ab-650
e
EIPA
Surface
TfR ab-650
inside TfR ab-650
f
Rac1 T17N
GFP-RacT17N

Sup Figure 5

Supplementary figure 5: Aggregation-dependent endocytosis re-routes transferrin receptor. (a) Aggregation of transferrin receptor ecto-domain (TfR) by antibody-mediated cross-linking in vitro. TfR was mixed with anti-transferrin receptor antibody 289 ± secondary antibody (2nd). Samples were spun to separate soluble (S) from insoluble/aggregated protein (P). Total protein coomassie stain is shown, the arrow indicates the MW of the TfR band that moved to the pellet. **(b, c)** Endocytosis of cross-linked transferrin receptor is independent of clathrin and dynamin, but transferrin remains dependent. SkBr3 cells transfected with control (GFP only) and dominant negative AP180ct and DynaminS45N N-terminally GFP tagged expression constructs were incubated with a panel of anti-TfR antibodies ± secondary antibody (2nd) and AlexaFluor546-transferrin for 30 min and analysed by flow-cytometry. Results for transferrin **(b)** and anti-TfR antibodies **(c)** endocytosis (means ± SD, n=3 independent experiments, ns non-specific $p>0.05$, , ** $p=0.0014$,**** $p<0.0001$ two-way ANOVA with Tukey's multiple comparison test.). **(d)** Endocytosis of cross-linked transferrin receptor is independent of clathrin and dynamin. SkBr3 cells transfected with control (GFP only) and dominant negative AP180ct N-terminally GFP tagged expression constructs were incubated with dylight650 labelled anti-TfR antibody ± secondary antibody (2nd) for 30 min and analysed confocal microscopy. **(e)** Cross-linked transferrin-receptor endocytosis is inhibited by 5-(N-ethyl-N-isopropyl)amiloride (EIPA). Hela cells were treated with 50 µM EIPA for 30 min, followed by incubation with dylight650 labelled anti-transferrin receptor antibody ± secondary antibody (2nd) for 30 min. After fixation surface bound antibody was counterstained and samples analysed by confocal microscopy. Subtraction of surface from total antibody signal yielded the endocytosed pool, which is shown in the bottom panels. **(f)** Cross-linked transferrin-receptor endocytosis requires Rac1. Hela cells were transfected with a vector expressing a dominant-negative mutant (T17N) of Rac1 for 16h incubated with

antibodies and analysed by confocal microscopy as described in (e). Transfected cells in the bottom panels showing the endocytosed anti-TfR antibody pool are outlined.

Scale bars: 10 µm. Source data are provided as a Source Data file.

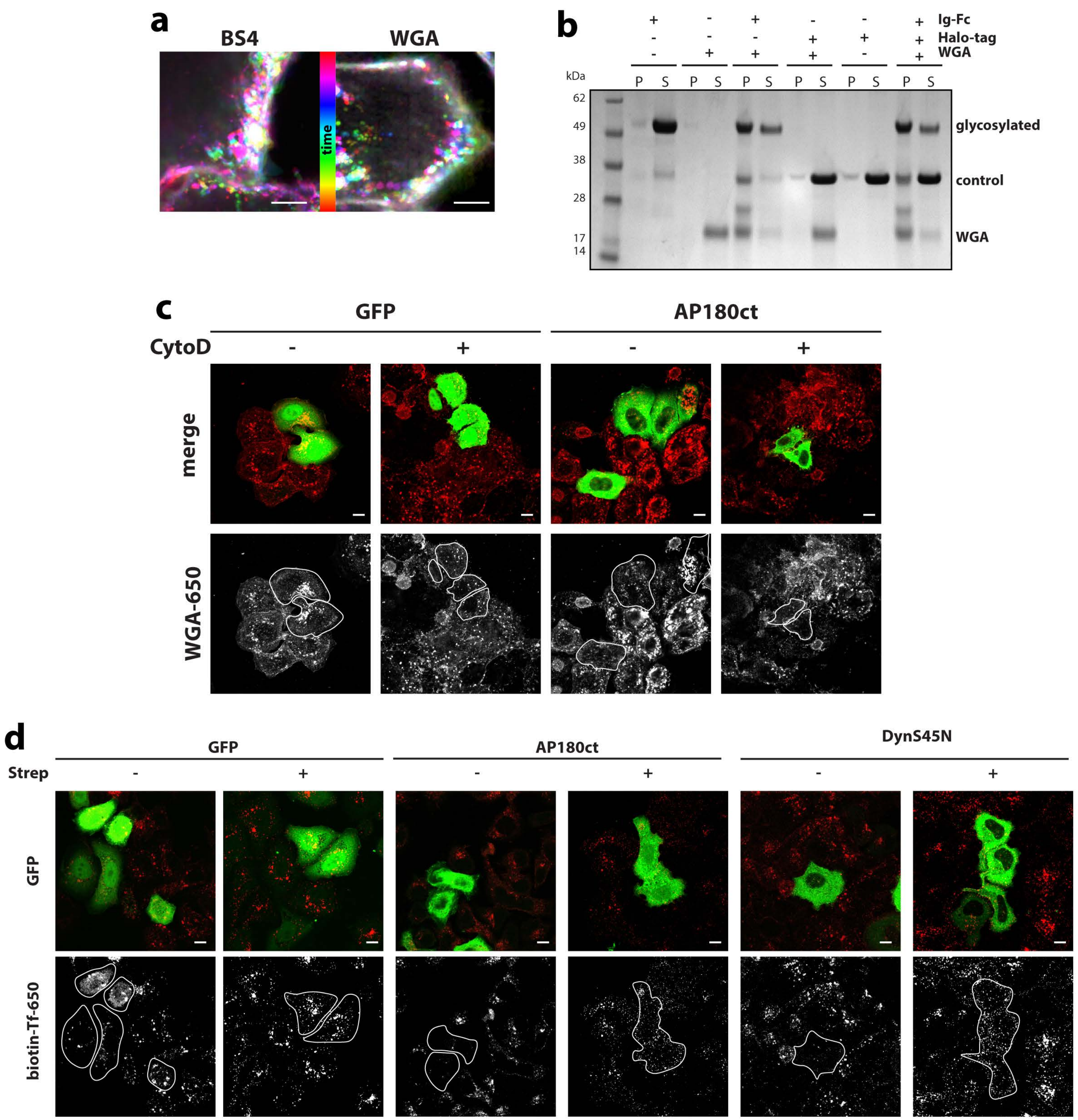
a
BS4
WGA
time
b
Ig-Fc
Halo-tag
WGA
kDa
P S
62
49
38
28
17
14
glycosylated
control
WGA
c
GFP
AP180ct
CytoD
merge
WGA-650
d
GFP
AP180ct
DynS45N
Strep
GFP
biotin-Tf-650

Sup Figure 6

Supplementary figure 6: Aggregation-dependent endocytosis is induced by cross-linking molecules. **(a)**Uptake dynamics of wheat-germ-agglutinin (WGA) resemble BS4 endocytosis. Dylight650 labelled WGA or biparatopic antibody BS4 was added onto SkBr3 cells, and imaging started immediately after (see also movie 8). Maximum intensity time projections over the imaging period are displayed, with time frames color-coded as indicated. **(b)** WGA specifically aggregates glycosylated proteins in vitro. WGA was mixed with recombinant glycosylated and non-glycosylated (control) protein as indicated. Samples were spun to separate soluble (S) from insoluble/aggregated protein (P). Total protein coomassie stain is shown. **(c)** WGA uptake is mediated by both clathrin-dependent and actin-dependent endocytic pathways. Cells transfected with dominant negative AP180ct N-terminally GFP tagged expression construct were incubated ± CytochalasinD (CytoD) with dylight650 labelled WGA and analysed by confocal microscopy. **(d)** Endocytosis of TfR by cross-linked transferrin is independent of clathrin and dynamin. SkBr3 cells transfected with control (GFP only) and dominant negative AP180ct and DynaminS45N N-terminally GFP tagged expression constructs were incubated with dylight650 labelled, biotinylated transferrin ± streptavidin (strep) for 30 min and analysed confocal microscopy

Scale bars: 10 µm. Source data are provided as a Source Data file.

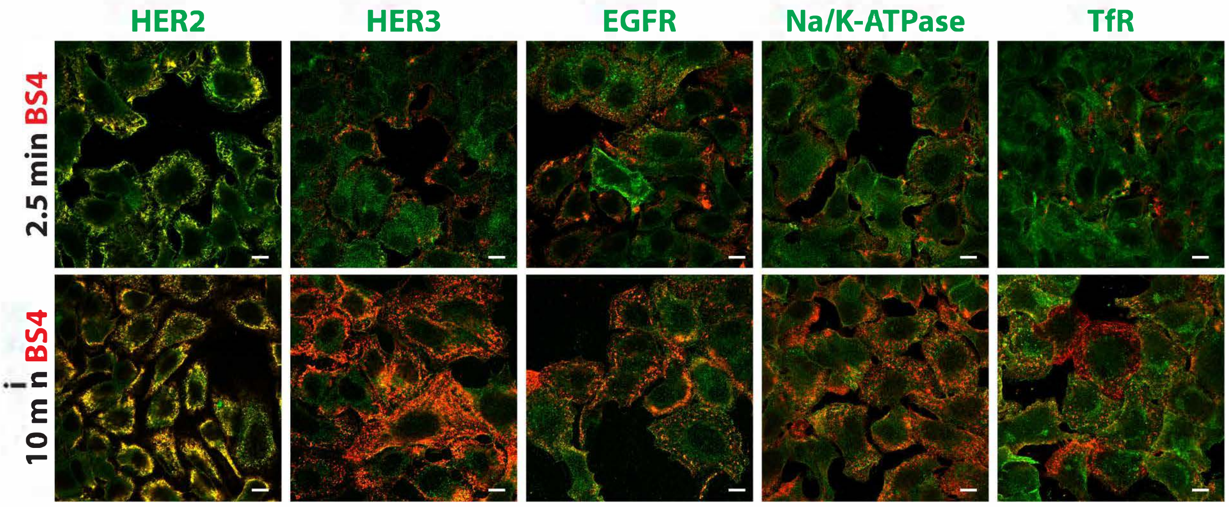

Sup Figure 7

Supplementary figure 7: BS4 clusters HER2 but no other receptors in the plasma membrane resulting in HER2-specific endocytosis. SkBr3 cells were incubated with HER2-specific, dylight650 labelled biparatopic antibody BS4 (red) for indicated times. After fixation, total receptor tyrosine-protein kinase ErbB-2 (HER2), receptor tyrosine-protein kinase ErbB-3 (HER3), epidermal growth factor receptor (EGFR), sodium/potassium-transporting ATPase Alpha1 (Na/K-ATPase) and Transferrin receptor 1 (TfR) in cell sections were stained (green) and samples were analysed by confocal microscopy.

Scale bars: 10 µm.

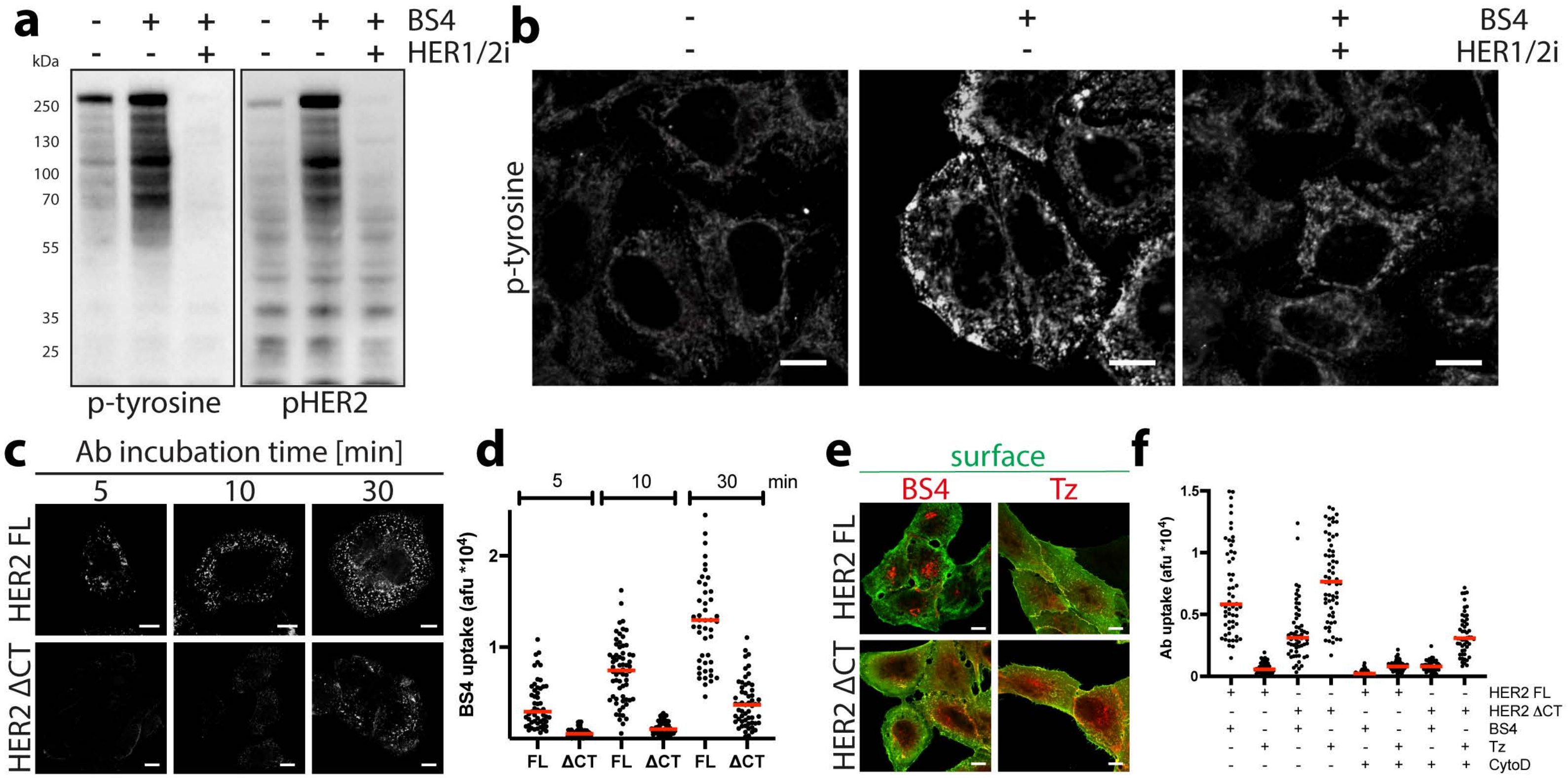
a
BS4
HER1/2i
kDa
250
130
100
70
55
35
25
p-tyrosine
pHER2
b
BS4
HER1/2i
p-tyrosine
c
Ab incubation time [min]
5
10
30
HER2 FL
HER2 ΔCT
d
5
10
30
min
BS4 uptake (afu *10⁴)
FL
ΔCT
e
surface
BS4
Tz
HER2 FL
HER2 ΔCT
f
Ab uptake (afu *10⁴)
HER2 FL
HER2 ΔCT
BS4
Tz
CytoD

Sup Figure 8

Supplementary figure 8: BS4-triggered ADE of HER2 requires autophosphorylation in the C-terminal domain. BS4 induces Her2 auto-phosphorylation **(a,b)**. SkBr3 cells ± dual EGFR/HER2 kinase inhibitor lapatinib (HER1/2i) were incubated with BS4 for 2.5 min as indicated. Samples were analysed by immunoblot for phospho-tyrosine residues (p-Y) and phospho-HER2 (pHER2 **(a)** related to Fig. 6a. SkBr3 cells ± dual EGFR/HER2 kinase inhibitor lapatinib (HER1/2i) were incubated with BS4-dylight650 for 2.5 min, after fixation stained for phosphorylated tyrosine residues (p-Y) and analysed by confocal microscopy **(b)** related to Fig. 6b. BS4 uptake in cells expressing full-length vs. kinase deficient HER2 **(c-f)**. U2OS cells ectopically expressing full-length (FL) HER2 or lacking the C-terminal cytoplasmic domain (HER2 ΔCT) were incubated with BS4-dylight650 for indicated times. After fixation surface bound BS4 was counterstained and samples analysed by confocal microscopy. Subtraction of surface from total antibody signal represented the endocytosed pool **(c)**. Results are quantified in **(d).** Dots represent measurements from individual cells, red lines indicate the median; n≥50 cells from 3 independent experiments. **(e)** U2OS cells ectopically expressing full-length (FL) HER2 or lacking the C-terminal cytoplasmic domain (HER2 ΔCT) were incubated with TZ-or BS4-dylight650 (red) for 30 min**.** After fixation surface bound antibodies were counterstained (green) and samples analysed by confocal microscopy. **(f)** U2OS cells ectopically expressing full-length (FL) HER2 or lacking the C-terminal cytoplasmic domain (HER2 ΔCT) were incubated with CytoD and TZ-or BS4-dylight650 for 30 min as indicated**.** Samples were processed and anlaysed as described in **(c)**. A quantification of antibody uptake is shown in **(f)**. Dots represent measurements from individual cells, red lines indicate the median; n≥50 cells from 3 independent experiments.

Scale bars: 10 µm. Source data are provided as a Source Data file.

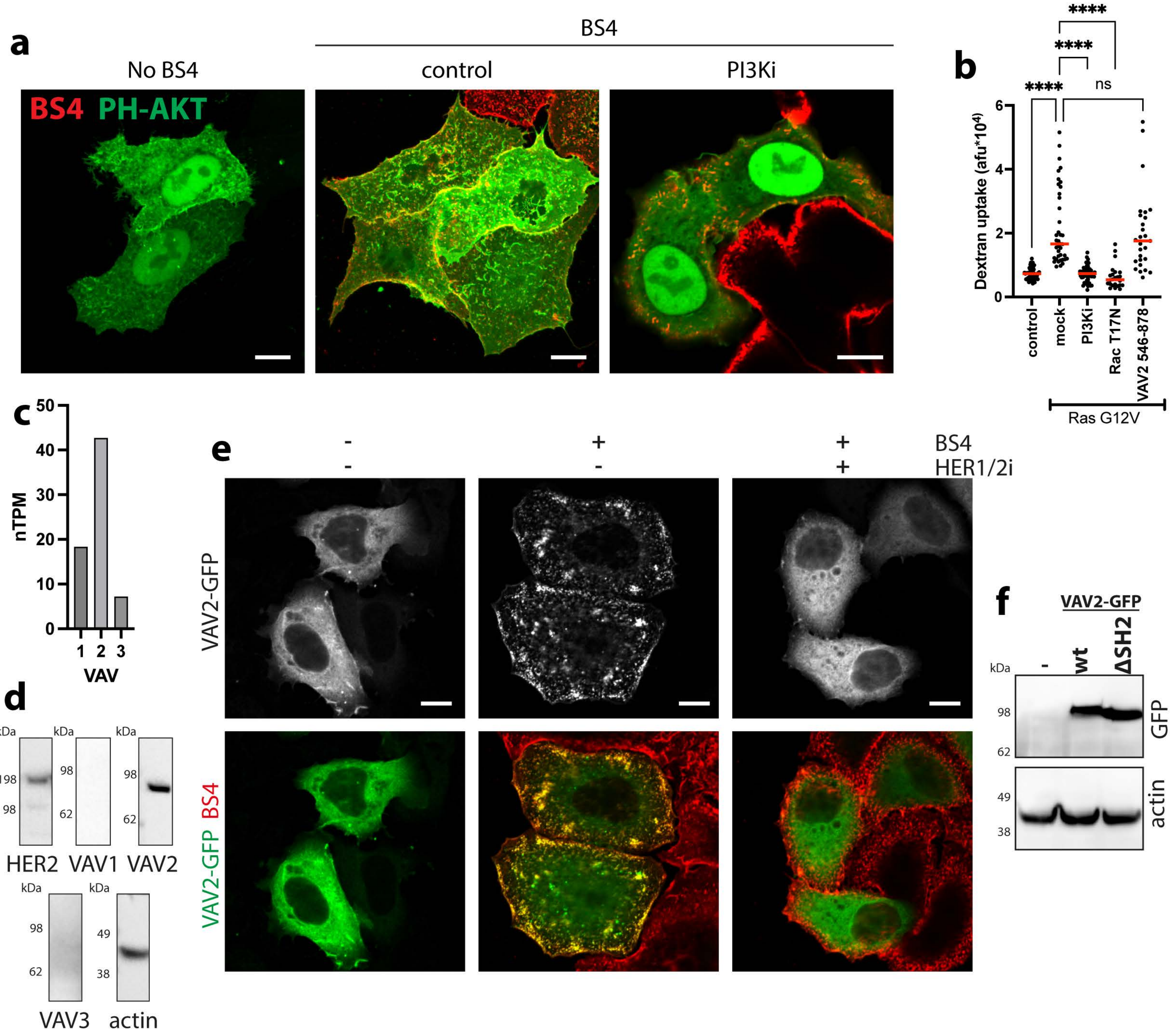

a
BS4
No BS4
control
PI3Ki
BS4 PH-AKT
b
****
****
****
ns
Dextran uptake (afu*10^4)
6
4
2
0
control
mock
PI3Ki
Rac T17N
VAV2 546-878
Ras G12V
c
50
40
30
20
10
0
nTPM
1
2
3
VAV
d
kDa
198
98
98
62
98
62
HER2
VAV1
VAV2
98
62
49
38
VAV3
actin
e
-
+
+
BS4
-
-
+
HER1/2i
VAV2-GFP
VAV2-GFP BS4
f
VAV2-GFP
-
wt
ΔSH2
98
62
GFP
49
38
actin

Sup Figure 9

Supplementary figure 9: PI3K and VAV dependence of BS4 uptake and Ras-induced macropinocytosis. Localisation of PIP3 to BS4-induced HER2 aggregates **(a)** related to Fig. 7c. SkBr3 cells were transfected with a vector expressing the PIP3 sensor PH-AKT-GFP (green) for 16h treated or not with the PI3K inhibitor LY294002 (PI3Ki) followed by incubation with BS4-dylight650 (red) for 2.5 min. After fixation samples were analysed by confocal microscopy. PI3K and Rac dependence of Ras-induced dextran uptake **(b)**. SkBr3 cells stably expressing constitutively active mutant Ras (G12V) were transfected with Rac1 dominant negative mutant (T17N) or dominant-negative VAV2 comprising only the interaction domains (amino acids 546-878) C-terminally GFP tagged expression vectors for 16 h or treated with LY294002 (PI3Ki) as indicated and incubated with dextran(70kDa)-TMR for 30 min. After fixation samples were analysed by confocal microscopy and dextran uptake was quantified. Dots represent measurements from individual cells, red lines indicate the median; $n \geq 25$ cells from 3 independent experiments; ns (non-significant) $p>0.05$, **** $p<0.0001$; one-way ANOVA with Sidak's multiple comparison test. Expression of VAV1-3 in SkBr3 cells **(c, d)**. Normalized transcript expression values (nTPM) retrieved from ProteinAtlas.org for VAV1-3 in SKBR3 cells are displayed **(c)**. SkBr3 cells were immunoblotted for VAV1-3 and HER2 and actin as controls **(d)**. BS4-induced interaction of HER2 with VAV2 is phospho-tyrosine dependent **(e)**. SkBr3 cells were transfected with VAV2 C-terminally GFP tagged expression vector (green) for 16h treated with lapatinib (HER1/2i) and incubated with BS4-dylight650 (red) for 2.5 min as indicated. VAV2 wt and ΔSH2 (lacking residues 665-772) steady-state protein levels **(f)** related to Fig. 8b. SkBr3 cells were transfected with VAV2 wt or a lacking residues 665-772 (ΔSH2) C-terminally GFP tagged expression vector for 16h followed by immunoblot analysis for GFP and actin as a loading control.

Scale bars: 10 µm. Source data are provided as a Source Data file.

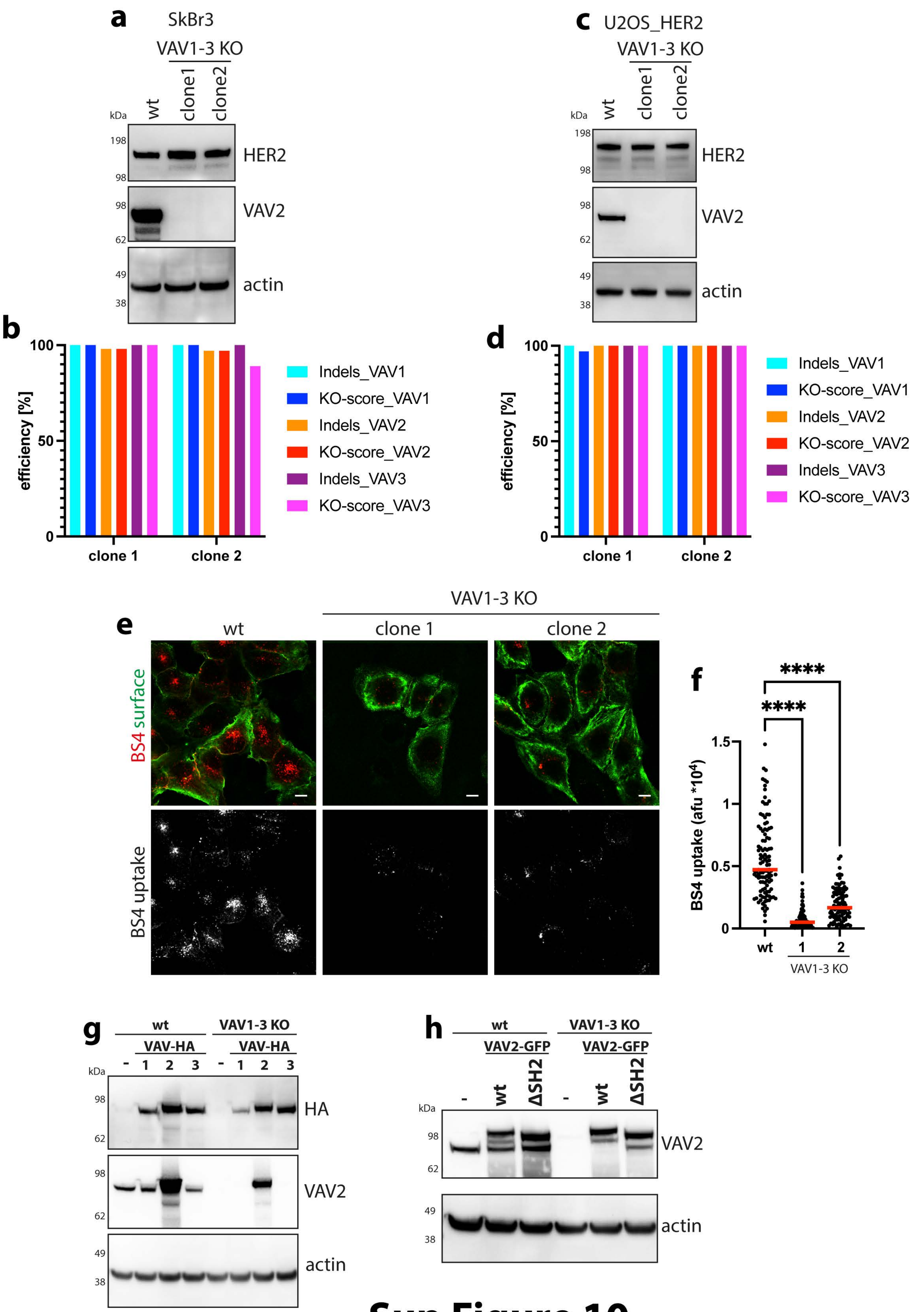

Sup Figure 10

Supplementary figure 10: BS4-induced ADE of HER2 requires VAV proteins. Characterisation of VAV1-3 KO cells **(a-d)**. Wt as well as VAV1-3 KO SkBr3 (a) and U2OS_HER2 (c) cells were immunoblotted for VAV2, HER2 and actin. VAV1-3 KO cell clones in SkBr3 (b) and U2OS_HER2 (d) backgrounds were analysed by sequencing of genomic loci targeted by gRNAs. Sanger sequencing files were analyses using the Inference of CRISPR Edits (ICE) tool from ice.synthego.com. VAV proteins are required for BS4 uptake in U2OS cells ectopically expressing HER2 **(e, f)**. U2OS_HER2 wt or VAV1-3 knock-out (KO) cells were incubated with BS4-dylight650 (red) for 30 min. After fixation, surface bound BS4 antibody was counterstained (green) and samples were analysed by confocal microscopy **(e)**. Subtraction of surface from total BS4 signal shows endocytosed pool (BS4 uptake)**.** Results are quantified in **(f).** Dots represent measurements from individual cells, red lines indicate the median; n≥50 cells from 3 independent experiments, **** $p<0.0001$; one-way ANOVA with Dunnett's multiple comparison test. VAV 1-3 steady-state protein levels (**g**) related to Fig. 6n. SkBr3 wt or VAV1-3KO cells were transfected with C-terminally HA-tagged VAV1-3 expression vectors for 16 h followed by immunoblot analysis for HA, VAV2 and actin as a loading control. VAV2 wt and ΔSH2 (lacking residues 665-772) steady-state protein levels (**h**) related to Fig. 6o. SkBr3 wt and VAV1-3KO cells were transfected with VAV2 wt or a lacking residues 665-772 (ΔSH2) C-terminally GFP tagged expression vectors for 16h followed by immunoblot analysis for VAV2 and actin as a loading control.

Scale bars: 10 µm. Source data are provided as a Source Data file.

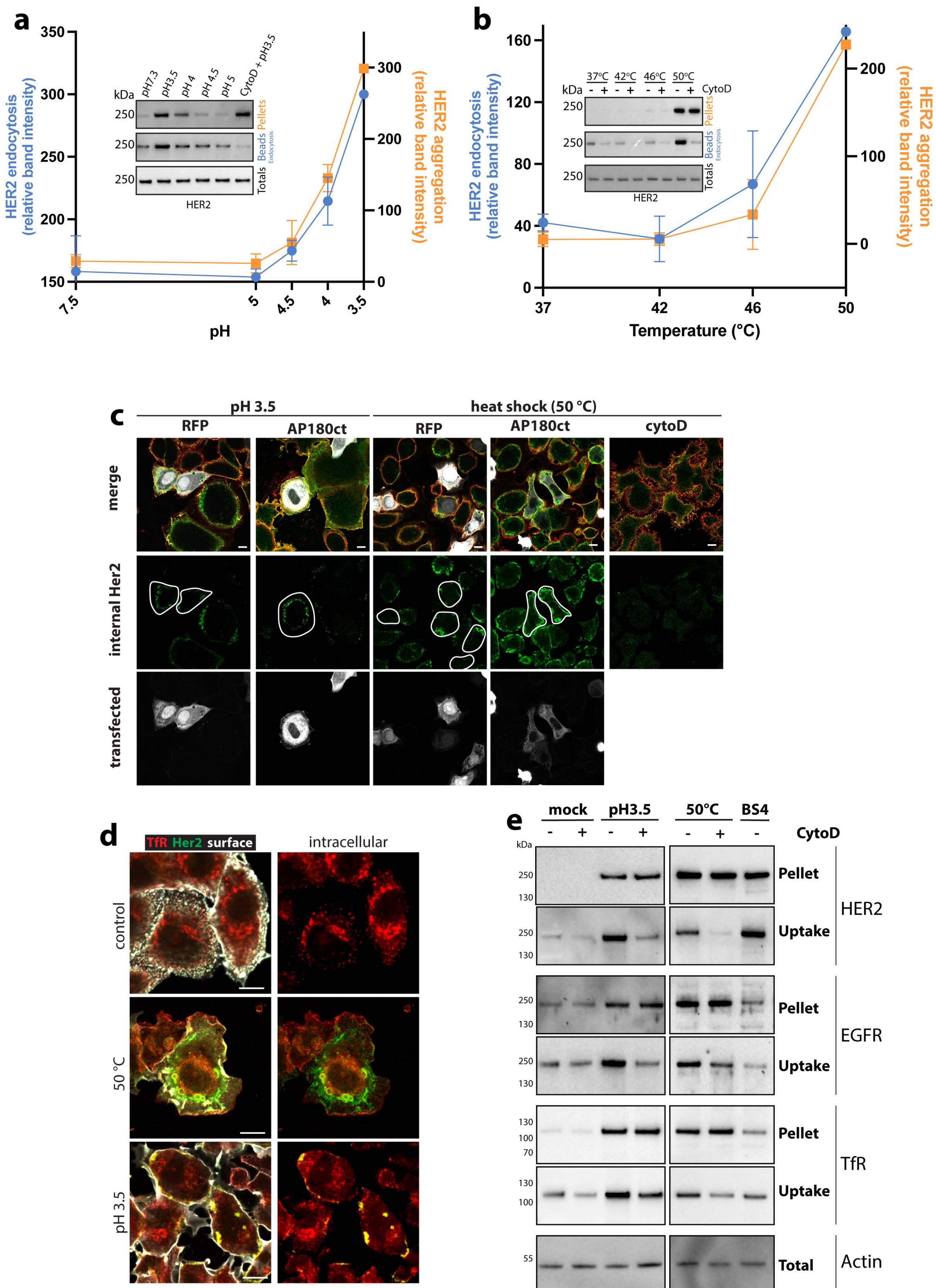

Sup Figure 11

Supplementary figure 11: Stress-induced cell surface receptor aggregates are endocytosed by ADE. (a) Titration of pH dependence of HER2 aggregation and endocytosis. After surface biotinylation, SkBr3 cells ± CytochalasinD (CytoD) were washed in buffers of different pH. After incubation of all samples at 37°C for 30 min, surface remaining biotin was removed and samples lysed in low detergent buffer. A fraction was spun to precipitate insoluble/aggregated proteins (Pellet). Protein in the remaining sample was solubilised (see materials and methods for protocol) and endocytosed biotinylated proteins concentrated on Streptavidin beads (uptake). Samples were assayed by immunoblot for HER2. A representative western blot is shown along with the quantification of band intensities, means ±SD are displayed, n= 3 independent experiments . **(b)** Temperature dependence of HER2 aggregation and endocytosis. After surface biotinylation, SkBr3 cells ± CytochalasinD (CytoD) were incubated for 5 min at different temperatures. After incubation of all samples at 37°C for 30 min, samples were prepared and analysed as descried in (a). **(c)** Stress-induced aggregation-dependent endocytosis occurs independent of dynamin but requires actin polymerisation. SkBr3 cells transfected with RFP control, or dominant negative AP180ct N-terminally RFP tagged expression vector (RFP channel displayed in white) were heat shocked for 5 min (at 50 °C) ± CytochalasinD (CytoD) or washed in an acidic buffer (pH3.5) followed by 30 min incubation at 37 °C. After fixation surface HER2 was stained (false colour-coded in red) before permeabilization and staining for total HER2 (green channel). Cell sections were analysed by confocal microscopy and post-processed by subtraction of surface from total HER2 staining, which allowed for specific visualisation of intracellular HER2. **(d)** Co-localisation of HER2 and TfR in endocytic carriers after stress-induced ADE. SkBr3 cells were treated as described in (c) and stained for TfR (red) and HER2 (green). Right hand panels show intracellular signal after subtraction of cell surface signal (white) from green and red channels.

(e) Physical and chemical stress triggers ADE of multiple receptors. Samples were prepared as described in (a) and immunoblotted for HER2, EGFR and TfR. Actin blots are shown as loading controls.

Scale bars: 10 µm. Source data are provided as a Source Data file.

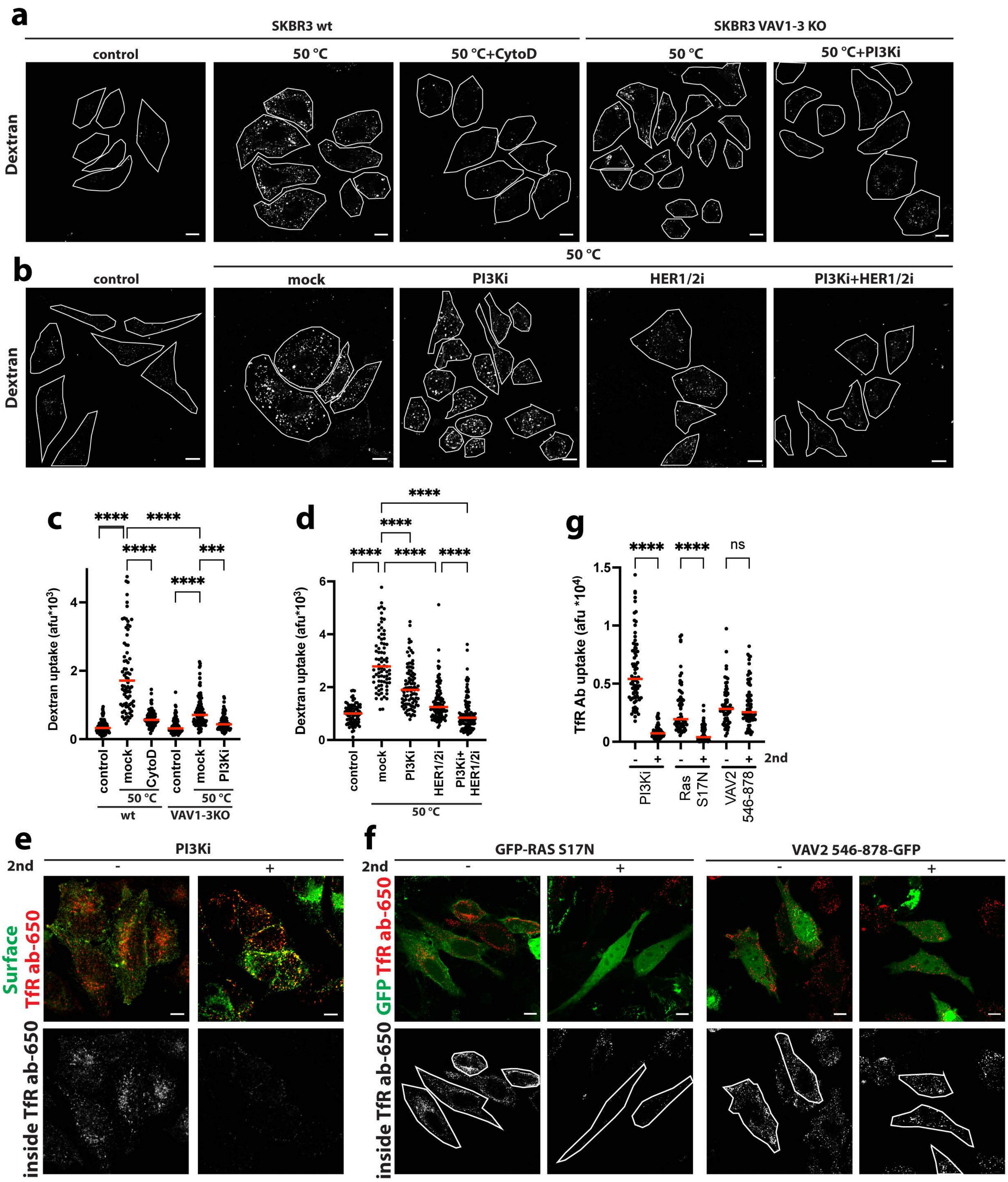

Sup Figure 12

Supplementary figure 12: Dependence of stress-induced ADE on PI3K and VAV proteins. Heat stress-induced fluid-phase uptake partially depends on VAV proteins, HER1/2 tyrosine-phosphorylation and PI3K **(a-d)**. Wt or VAV1-3 KO SkBr3 cells **(a,c)** or Hela cells **(b,d)** ± CytochalasinD (CytoD) or ±LY294002 (PI3Ki) or ±Lapatinib (HER1/2i) were incubated with dextran(70kDa)-fluorescein for 5 min at 50 °C followed by 30 min at 37°C. After fixation samples were analysed by confocal microscopy **(a,b)**. Quantification of heat-stress induced dextran uptake is shown in **(c,d)**. Dots represent measurements from individual cells, red lines indicate the median; n≥50 cells from 3 independent experiments, *** p=0.0003, **** p<0.0001; one-way ANOVA with Sidak's multiple comparison test. Antibody-mediated aggregation-dependent endocytosis of TfR depends on PI3K/Ras but is independent of VAV proteins **(e-g)**. Hela cells ± LY294002 (PI3Ki) were incubated with dylight650 labelled anti-transferrin receptor antibody 289 (red) ± secondary antibody (2nd) for 30 min. After fixation surface bound antibody was counterstained (green) and samples analysed by confocal microscopy. Subtraction of surface from total antibody signal yielded the endocytosed pool **(e)**. Hela cells were transfected with a plasmid expressing a dominant-negative mutant (S17N) of Ras or with a dominant-negative mutant of VAV2 comprising only the interaction domains (amino acids 546-878) for 16h followed by incubation with dylight650 labelled anti-transferrin receptor antibody 289 (red) **(f)**. Samples were treated and analysed as described in **(e)**. Quantification of anti-TfR antibody ± secondary antibody (2nd) uptake is shown in **(g)**. Dots represent measurements from individual cells, red lines indicate the median; n>50 from 3 independent experiments, ns (non-significant) p>0.05, **** p<0.0001; one-way ANOVA with Sidak's multiple comparison test.

Scale bars: 10 µm. Source data are provided as a Source Data file.

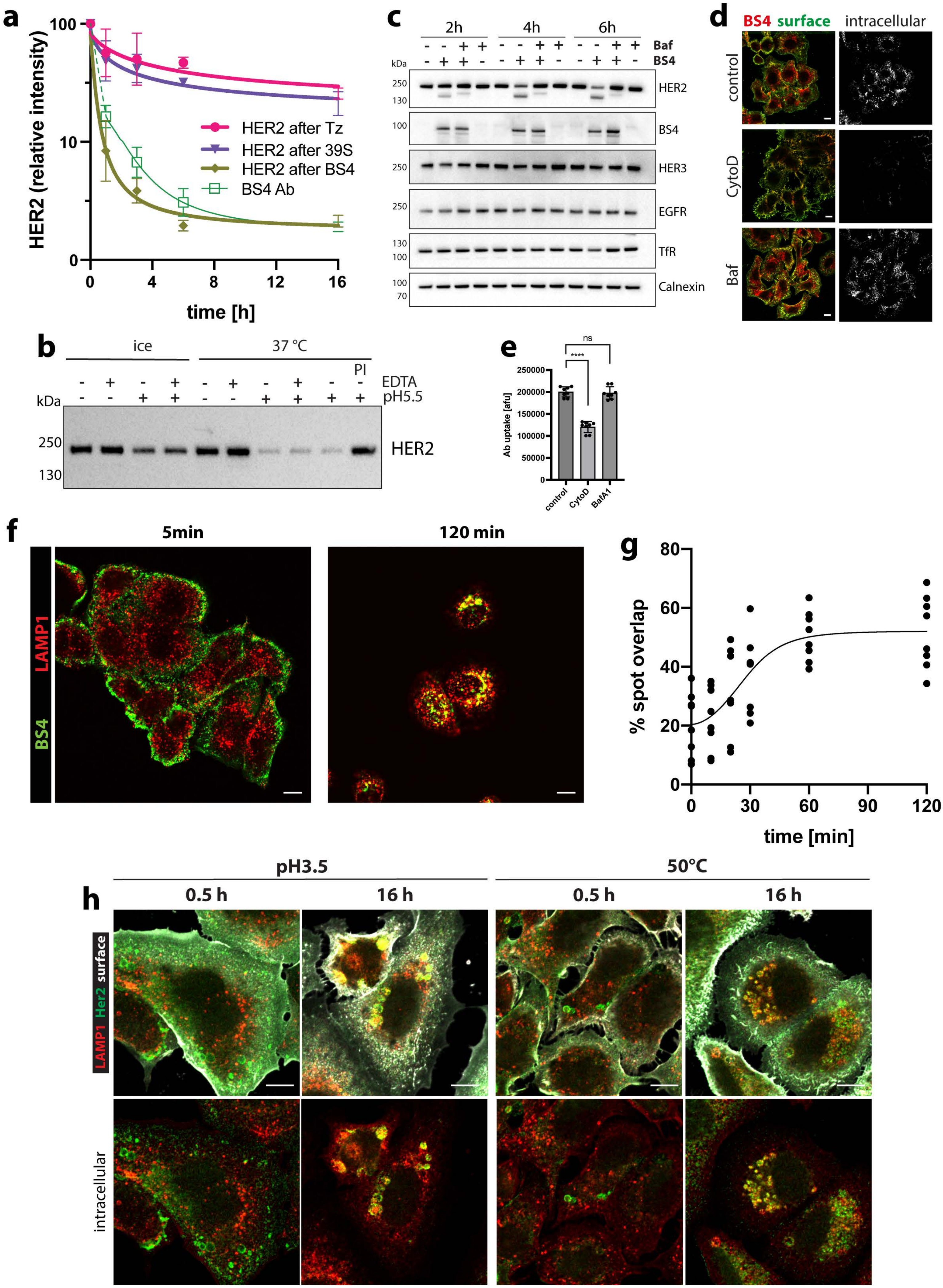

Sup Figure 13

Supplementary figure 13: Lysosomal degradation of HER2 receptor aggregates after BS4- and stress triggered ADE. (a) Quantitation of western blot analysis (see Fig 10a) of steady state HER2 protein levels after exposure to monotopic antibodies Tz and 39S and biparatopic BS4 for increasing length of time (means ± SD, n=3 independent experiments). **(b)** HER2 proteolysis is acid-dependent**.** SkBr3 cell lysates were incubated for 5 h under indicated conditions and blotted for HER2. **(c)** BS4-induced degradation is HER2-specific and blocked by inhibition of lysosomal acidification**.** SkBr3 cells were incubated with BS4 and ± bafilomycinA1 (Baf) for indicated times and blotted for HER2, the biparatopic Ab heavy chain (BS4) and other cell surface receptors as controls. **(d,e)** BS4-triggered ADE uptake is not inhibited by bafilomycinA1. SkBr3 cells treated with CytoD or Baf as indicated were incubated with dylight650 labelled BS4 antibody (red) for 30 min and after fixation and surface staining with anti-human 488 (green) analysed by confocal microscopy and post-processed by subtraction of surface from total BS4 staining, which allowed for specific visualisation of intracellular BS4 **(d)**, or flow-cytometry **(e)** means ± SD, n=8 independent experiments, ns (non-significant) $p>0.05$, **** $p<0.0001$, one-way ANOVA with Dunnett's multiple comparison test. **(f)** SkBr3 cells endocytosing BS4 were fixed after indicated times and stained for lysosomes using a LAMP-1 specific antibody. **(g)** Quantification of BS4 localisation to lysosomes (non-linear fitted curve, n=8 randomly chosen fields of view with a total of at least 40 cells per condition). **(h)** Co-localisation of stress-induced endocytic vesicles with LAMP-1. SkBr3 cells treated with Baf were heat shocked for 5 min (at 50 °C) or washed in an acidic buffer (pH3.5) followed by 0.5 or 16 h incubation at 37 °C. After fixation surface HER2 was stained (false colour-coded in white) before permeabilization and staining for total HER2 (green) and LAMP-1 (red). Cell sections were analysed by confocal microscopy and post-processed by subtraction of surface from total HER2 staining, which allowed for specific visualisation of intracellular HER2.

Scale bars: 10 µm. Source data are provided as a Source Data file.
