# Supplementary material for: Cell surface protein aggregation triggers endocytosis to maintain plasma membrane proteostasis
Source: Nat Commun. 2023 Feb 28;14:947. doi: 10.1038/s41467-023-36496-y (PMC9974993; doi:10.1038/s41467-023-36496-y)
Supplement: Supplementary file 3 — Description of Additional Supplementary Files [file 41467_2023_36496_MOESM3_ESM.pdf]

Description of Additional Supplementary Files

File name: Supplementary Movie 1

Description: BS4 marks large endocytic vesicles. Dynamics of BS4-dylight650 endocytosis in SkBr3 cells at indicated times after antibody addition. Time [min:sec]. Scale bar: 5 µm.

File name: Supplementary Movie 2

Description: BS4 binding does not alter plasma membrane movements. Cells expressing GPI-membrane bound GFP were imaged before and immediately after addition of BS4-dylight650. Time [min:sec].

File name: Supplementary Movie 3

Description: Concentration of surface bound BS4 by inwards moving lamellipodium. Cells expressing GPI-membrane bound GFP were imaged immediately after addition of BS4-dylight650. Time [min:sec]. Scale bar: 5 µm.

File name: Supplementary Movie 4

Description: 3D view of BS4 concentration by inwards moving lamellipodium wave. Cells expressing GPI-membrane bound GFP were imaged immediately after addition of BS4-dylight650.

File name: Supplementary Movie 5

Description: Rac1 inhibition prevents plasma membrane movement. Cells expressing GPI-membrane bound GFP were imaged before and after addition of Rac1 inhibitor EHT1864. Time [min:sec].

File name: Supplementary Movie 6

Description: Morphodynamics of BS4 uptake. Cells expressing GPI-membrane bound GFP were imaged immediately after addition of BS4-dylight650. Time [min:sec].

File name: Supplementary Movie 7

Description: BS4 uptake morphologically resembles macropinocytosis. Cells expressing GPI-membrane bound GFP were imaged immediately after addition of BS4-dylight650. 3D views, surface rendering of the plasma membrane (green) and frame number (1 frame/10s) are displayed.

File name: Supplementary Movie 8

Description: Uptake dynamics of wheat-germ-agglutinin (WGA) resemble BS4 endocytosis. Dylight650 labelled WGA or biparatopic antibody BS4 was added onto SkBr3 cells, and imaging started immediately after. Time [min:sec].
