# Supplementary material for: Cell surface protein aggregation triggers endocytosis to maintain plasma membrane proteostasis
Source: Nat Commun. 2023 Feb 28;14:947. doi: 10.1038/s41467-023-36496-y (PMC9974993; doi:10.1038/s41467-023-36496-y)
Supplement: Supplementary file 12 — Reporting Summary [file 41467_2023_36496_MOESM12_ESM.pdf]

nature portfolio

Corresponding author(s): Harvey T. McMahon

Last updated by author(s): Jan 9, 2023

Reporting Summary

Nature Portfolio wishes to improve the reproducibility of the work that we publish. This form provides structure for consistency and transparency in reporting. For further information on Nature Portfolio policies, see our Editorial Policies and the Editorial Policy Checklist.

Statistics

For all statistical analyses, confirm that the following items are present in the figure legend, table legend, main text, or Methods section.

n/a	Confirmed	
☐	☒	The exact sample size (*n*) for each experimental group/condition, given as a discrete number and unit of measurement
☐	☒	A statement on whether measurements were taken from distinct samples or whether the same sample was measured repeatedly
☐	☒	The statistical test(s) used AND whether they are one- or two-sided<br>*Only common tests should be described solely by name; describe more complex techniques in the Methods section.*
☒	☐	A description of all covariates tested
☒	☐	A description of any assumptions or corrections, such as tests of normality and adjustment for multiple comparisons
☐	☒	A full description of the statistical parameters including central tendency (e.g. means) or other basic estimates (e.g. regression coefficient) AND variation (e.g. standard deviation) or associated estimates of uncertainty (e.g. confidence intervals)
☐	☒	For null hypothesis testing, the test statistic (e.g. *F*, *t*, *r*) with confidence intervals, effect sizes, degrees of freedom and *P* value noted<br>*Give P values as exact values whenever suitable.*
☒	☐	For Bayesian analysis, information on the choice of priors and Markov chain Monte Carlo settings
☒	☐	For hierarchical and complex designs, identification of the appropriate level for tests and full reporting of outcomes
☒	☐	Estimates of effect sizes (e.g. Cohen's *d*, Pearson's *r*), indicating how they were calculated

Our web collection on statistics for biologists contains articles on many of the points above.

Software and code

Policy information about availability of computer code

Data collection	Live microscopy data was collected using the NIS Elements AR (V5.11.01) software package (Nikon). Fixed cell confocal images were acquired using the ZEN (V2011 SP7 & V3.5 Blue edition) software package (Zeiss). Western Blots were imaged using Image Lab (V5.2.1, Biorad). Incucyte-based monitoring of antibody uptake and cell growth were done using the IncuCyte S3 Software package (V2018B, Sartorius). FACS data was acquired using FACSDiva (V6.2, BD Biosciences).
Data analysis	Live microscopy images were deconvolved using the NIS Elements AR software package(V5.11.01, Nikon), and time-lapse 3D depth-color coded movies generated using the same software. Surface rendering of the plasma membrane was done using Imaris (V9.7.2 x64, Oxford Instruments). Maximum intensity Z and time projections were done in FIJI V2.3.0 (http://imagej.nih.gov/ij). Fixed cell confocal images were processed using FIJI. Band intensity on Western bolts was quantified using FIJI. Incucyte-based quantification of antibody uptake and cell growth was done using the IncuCyte S3 Software package (V2018B, Sartorius). FACS data was analyzed using FACSDiva (V6.2, BD Biosciences). Quantifications, statistics, and graphical representation of data were obtained using Excel (V15.24, Microsoft) and Prism (V8.4.3 & V9, Graphpad).

For manuscripts utilizing custom algorithms or software that are central to the research but not yet described in published literature, software must be made available to editors and reviewers. We strongly encourage code deposition in a community repository (e.g. GitHub). See the Nature Portfolio guidelines for submitting code & software for further information.

Data

Policy information about availability of data

All manuscripts must include a data availability statement. This statement should provide the following information, where applicable:

- Accession codes, unique identifiers, or web links for publicly available datasets
- A description of any restrictions on data availability
- For clinical datasets or third party data, please ensure that the statement adheres to our policy

Expression constructs of human proteins are based on the following reference sequences available at https://www.ncbi.nlm.nih.gov/nucleotide/: HER2 NM_004448.4, Rac1 NM_006908.5, Ras1 NM_005343.4. VAV1-3 expression data in Fig. S8i is from proteinatlas.org: VAV1 (ENSG00000141968), VAV2 (ENSG00000160293), VAV3 (ENSG00000134215).
The authors declare that all other data supporting the findings of this study are available within the paper and its supplementary information files.

Human research participants

Policy information about studies involving human research participants and Sex and Gender in Research.

Reporting on sex and gender	N/A
Population characteristics	N/A
Recruitment	N/A
Ethics oversight	N/A

Note that full information on the approval of the study protocol must also be provided in the manuscript.

Field-specific reporting

Please select the one below that is the best fit for your research. If you are not sure, read the appropriate sections before making your selection.

☒ Life sciences ☐ Behavioural & social sciences ☐ Ecological, evolutionary & environmental sciences

For a reference copy of the document with all sections, see nature.com/documents/nr-reporting-summary-flat.pdf

Life sciences study design

All studies must disclose on these points even when the disclosure is negative.

Sample size	No statistical method was used to predetermine the sample size, but given the observed small variance among independent experimental replicates we considered the following sample size sufficient:<br>For microscopy based experiments at least three independent replicates were performed and quantifications are based on 10 randomly chosen fields of view.<br>FACS data are based on three independent replicates analyzing at least 10,000 cells each.<br>Incucyte data are based on three independent replicates, with 6-8 replicate well per experiment.<br>Western blots show a representative results of three independent replicates, and band quantitations are based on data obtained from the three replicates.
Data exclusions	Replicate experiments were excluded from the analysis in case that cell viability had been severely compromised due to technical or unknown faults.
Replication	At least three independent experimental replicates were conducted. The replication experiments were performed on different days and thus using a variation in cell passage number. All replications were successful.
Randomization	Cell cultures were randomly assigned to different experimental treatment groups.
Blinding	Investigators were not blinded to experimental groups during data acquisition and analysis. However, quantification analysis is either assisted by or fully based on computer-aided signal detection applying the same parameters. Hence, initial blinding was not performed as the same qauntification criteria were applied to different experimental groups.

Reporting for specific materials, systems and methods

We require information from authors about some types of materials, experimental systems and methods used in many studies. Here, indicate whether each material, system or method listed is relevant to your study. If you are not sure if a list item applies to your research, read the appropriate section before selecting a response.

Materials & experimental systems

n/a	Involved in the study
☐	☒ Antibodies
☐	☒ Eukaryotic cell lines
☒	☐ Palaeontology and archaeology
☒	☐ Animals and other organisms
☒	☐ Clinical data
☒	☐ Dual use research of concern

Methods

n/a	Involved in the study
☒	☐ ChIP-seq
☐	☒ Flow cytometry
☒	☐ MRI-based neuroimaging

Antibodies

Antibodies used

HER2-specific antibodies Trastuzumab, 39S, BS4 as well as TfR–specific antibodies 226, 289 and 292 were produced by AstraZeneca and are available upon request.
Antibodies recognising the HER2 cytoplasmic domain (2242), pHER2 (2241), HER3 (12708), the HA-tag (2367) and EGFR (4267) were from Cell Signaling Technology. An antibody recognising the HER2 ecto-domain (AF1129) was from R&D Systems. Antibodies recognising VAV1 (Ab245440), VAV2 (Ab52640), VAV3 (Ab52938), GFP (ab290), actin (ab6276), calnexin (ab22595) were from Abcam. Other antibodies were, phospho-tyrosine (P5872, Merck), Rac1 (610650, BD biosciences), TfR (13-6800, Thermo Fisher Scientific), Na/K-ATPase (NB300-146, Novus Biologicals) and Lamp1 (clone H4A3, Developmental Studies Hybridoma Bank). Secondary antibodies: anti-human unconjugated (A18819) anti-human Alexa488 (A11013), anti-human Alexa568 (A21090), anti-human Alexa647 (A21445), anti-rabbit Alexa488 (A11008), anti-mouse Alexa488 (A11001), anti-mouse Alexa568 (A11004) were from Thermo Fisher Scientific. HRP-conjugated secondary antibodies: anti-mouse (172-1011) anti-human (172-1033) anti-rabbit (172-1019) and anti-goat (172-1034) were from BioRad.

Validation

HER2-specific antibodies Trastuzumab, 39S, BS4 have been previously validated (Li, J. Y. et al. https://doi.org:10.1016/j.ccell.2015.12.008). Human TfR–specific antibodies 226, 289 and 292 were validated to recognize folded epitopes in Tfr ecto-domain in https://www.lens.org/lens/patent/194-315-831-066-396/fulltext and https://www.lens.org/lens/patent/101-316-354-557-993/fulltext.
Human-specific antibody recognizing the HER2 cytoplasmic domain (2242) was validated for Western-blot and Immunohistochemistry by Cell Signaling Technology. The anti-pHER2 (2241) antibody recognizing human and mouse Phospho-HER2/ErbB2 (Tyr877) was validated for Western-blot by Cell Signaling Technology. HA-tag-specific antibody (2367) was validated for Western-blot and Immunofluorescence by Cell Signaling Technology. The anti-EGFR (2241) antibody recognizing human, monkey and mouse EGFR was validated for Western-blot and Immunofluorescence by Cell Signaling Technology. Human-specific antibody recognizing the HER2 ecto-domain (AF1129) was validated for Western-blot by R&D Systems. Human VAV1-specific antibody (Ab245440) was validated for Western-blot by abcam. Recombinant Anti-VAV2 antibody (Ab52640) recognising human and mouse VAV2 was validated in knock-out cells by Western-blot by abcam. Recombinant Anti-VAV3 antibody (Ab52938) recognizing human VAV3 was validated in knock-out cells by Western-blot by abcam. Anti-GFP antibody (ab290) was validated for Western-blot and immunofluorescence by abcam. Anti-actin (ab6276) recognising Mouse, Rat, Cow, Dog, Human, African green monkey, Chinese hamster beta-actin was KO-validated for Western-blot and immunofluorescence by abcam. KO-validated anti-calnexin antibody specific for mouse, rat and human was validated for Western-blot and Immunofluorescence by abcam. Anti-phosphotyrosine antibody was validated for western blot and immunofluorescence by Merck. Anti-Rac1 antibody (610650) was validated for Western-blot and immunofluorescence by BD biosciences. Anti-Na/K-ATPase recognizing human, mouse, rat, bovine, canine xenopus, and yest protein was validated for western-blot and immunofluorescence by Novus Biologicals. Anti-Lamp1, recognizing human, monkey, hamster and rat protein was validated for western-blot and immunofluorescence by Developmental Studies Hybridoma Bank.

Eukaryotic cell lines

Policy information about cell lines and Sex and Gender in Research

Cell line source(s)

SkBr3 (ATCC HTB-30), SkOv3 (ATCC HTB-770), MCF7 (ATCC HTB-22), Hela cells (ECACC 93021013), U2OS cells (ECACC 92022711), CHO-K1 cells (ATCC CCL-61), Spodoptera frugiperda (Sf9) cells (Thermo Fisher Scientific 11496015)

Authentication

Cell line authentication was performed by ATCC and ECACC using STR analysis.

Mycoplasma contamination

All cell lines were routinely checked and tested negative for Mycoplasma contamination.

Commonly misidentified lines
(See ICLAC register)

None of the cell lines used in this study are listed in the ICLAC register.

Flow Cytometry

Plots

Confirm that:

☒ The axis labels state the marker and fluorochrome used (e.g. CD4-FITC).

☒ The axis scales are clearly visible. Include numbers along axes only for bottom left plot of group (a 'group' is an analysis of identical markers).

☒ All plots are contour plots with outliers or pseudocolor plots.

☒ A numerical value for number of cells or percentage (with statistics) is provided.

Methodology

Sample preparation	Cells were detached using trypsin, pelleted, resuspended in 100 mM Tris pH 8.5 150 mM NaCl and 50 mM TCEP and immediately examined.
Instrument	LSRFortessa cell analyser (BD Bioscience)
Software	FACSDiva Version 6.2 was used to acquire and analyze flow cytometry data.
Cell population abundance	A minimum of 10,000 cells were assessed with laser lines appropriate to the fluorophores used.
Gating strategy	Initial gating was area vs height to eliminate doublets and gating on forward and side scatter to exclude debris. Cells selected by this strategy were further gated on GFP if transfected, providing intrinsic untransfected controls for each cell population. Transferrin and BS4 uptake was measured in the red and far-red channel, respectively both in untransfected (GFP-) and transfected (GFP+) cells.

☒ Tick this box to confirm that a figure exemplifying the gating strategy is provided in the Supplementary Information.
