# Supplementary material for: Cell surface protein aggregation triggers endocytosis to maintain plasma membrane proteostasis
Source: Nat Commun. 2023 Feb 28;14:947. doi: 10.1038/s41467-023-36496-y (PMC9974993; doi:10.1038/s41467-023-36496-y)
Supplement: Supplementary file 13 — Source Data [file 41467_2023_36496_MOESM13_ESM.zip › Source data_blotsandgels figures and supplementary figures.pdf]

Figure 3b

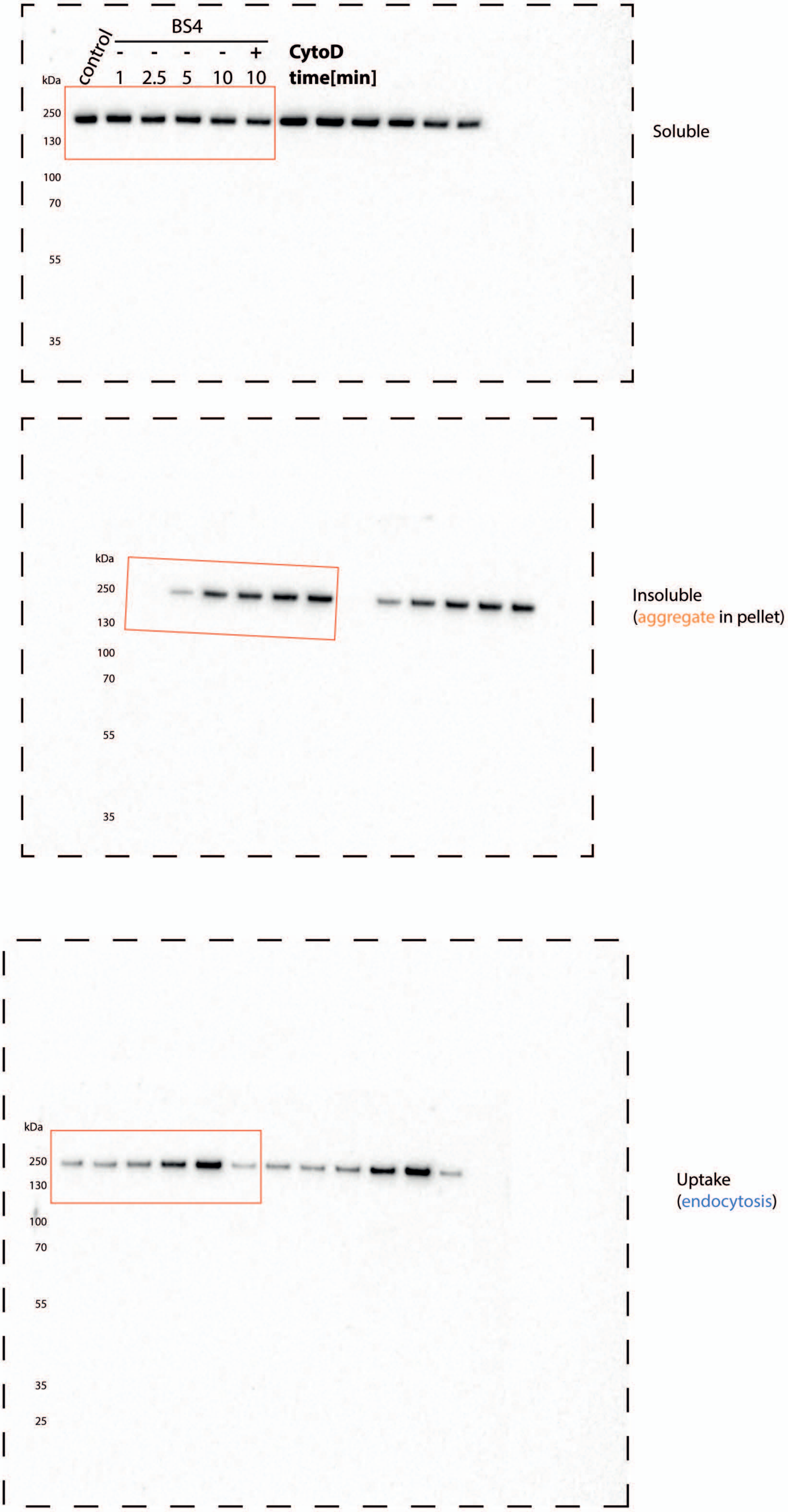

HER2

Figure 3d

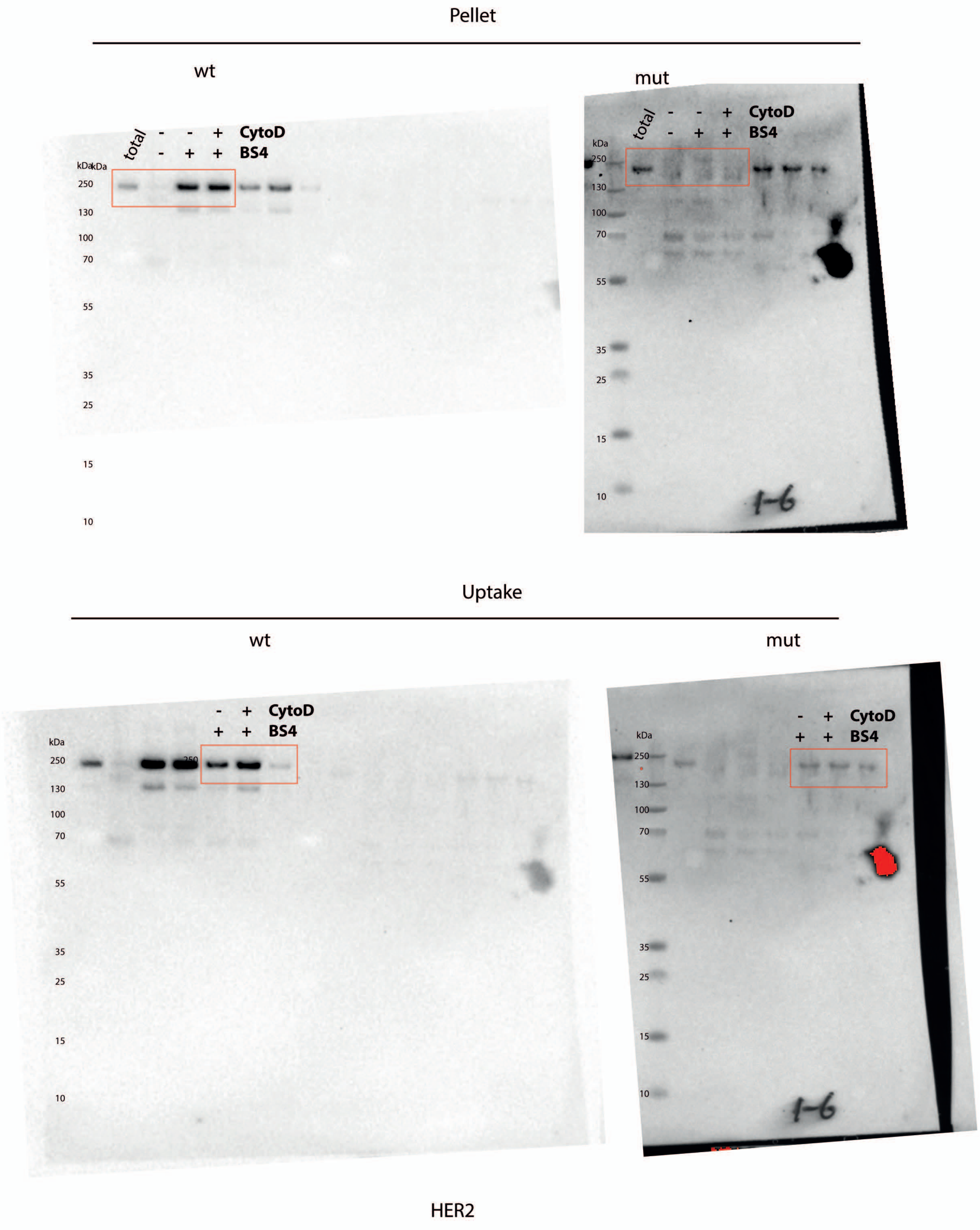
Pellet
wt
total
- - + CytoD
- + + BS4
kDa
250
130
100
70
55
35
25
15
10
mut
total
- - + CytoD
- + + BS4
kDa
250
130
100
70
55
35
25
15
10
Uptake
wt
- + CytoD
+ + BS4
kDa
250
130
100
70
55
35
25
15
10
mut
- + CytoD
+ + BS4
kDa
250
130
100
70
55
35
25
15
10
HER2

Figure 4a

Pellet

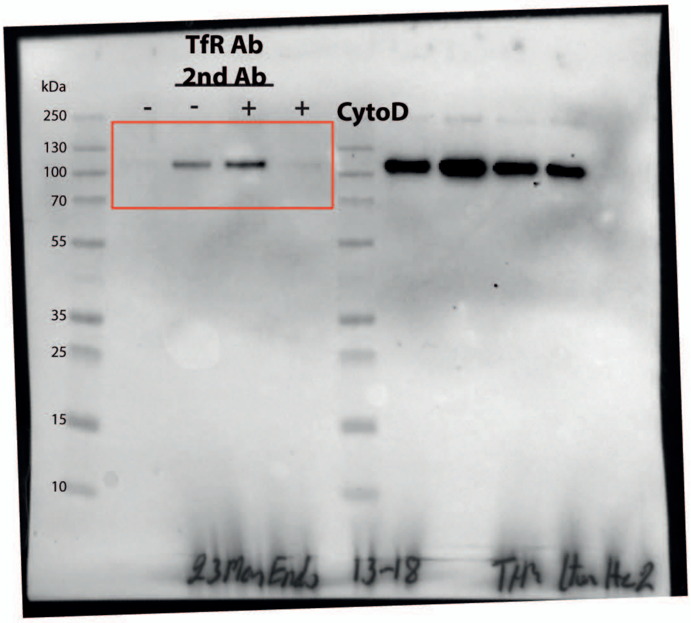

Uptake

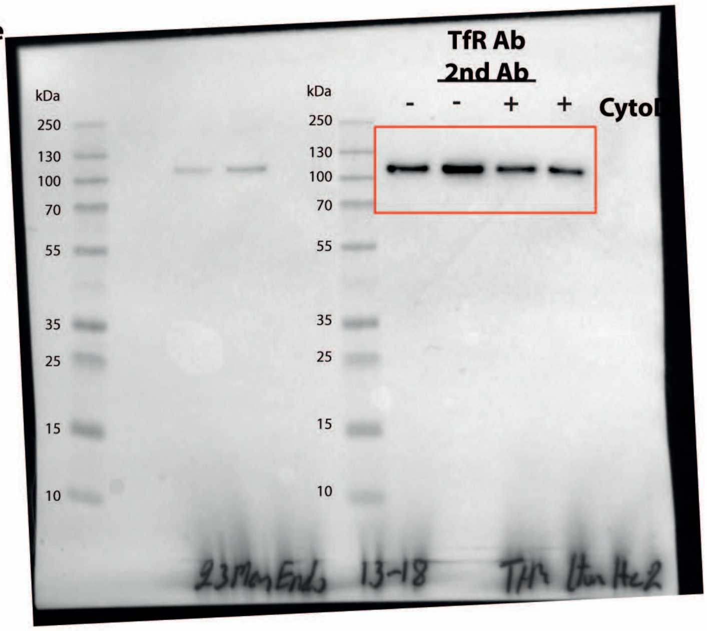

TfR

Figure 5a 1st part

HER2

Pellet

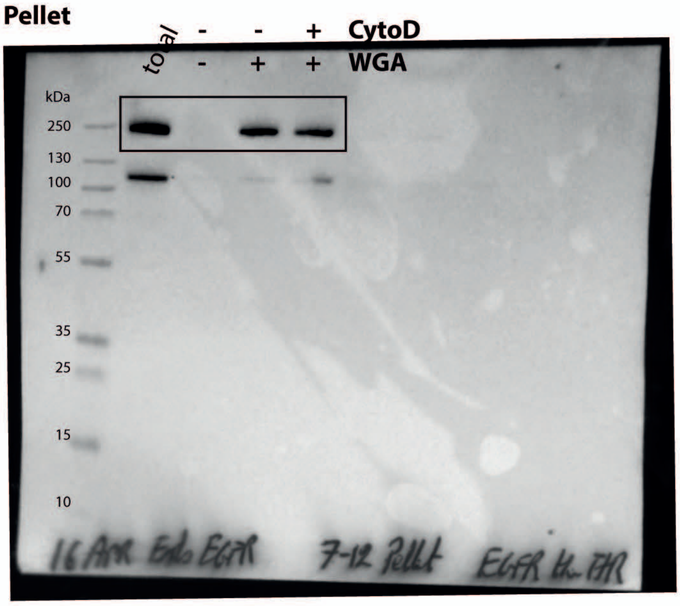

Uptake

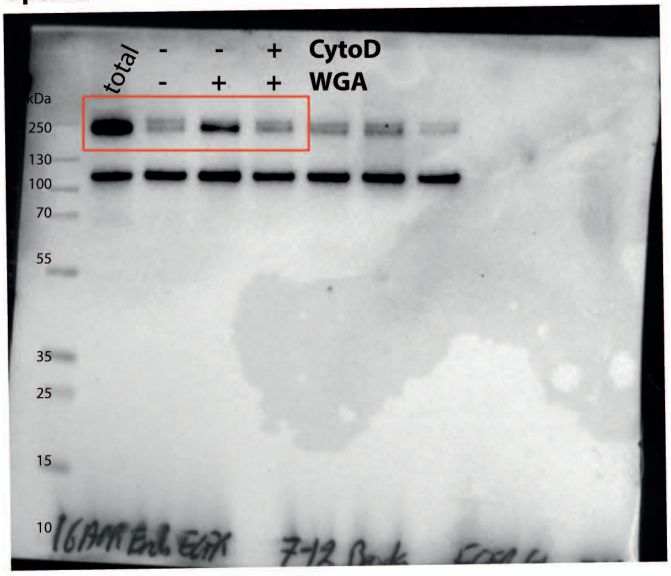

EGFR

Pellet

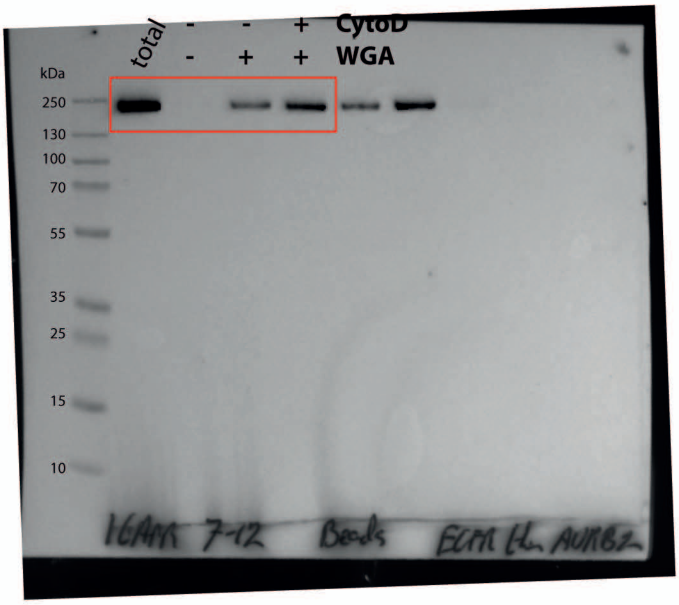

Uptake

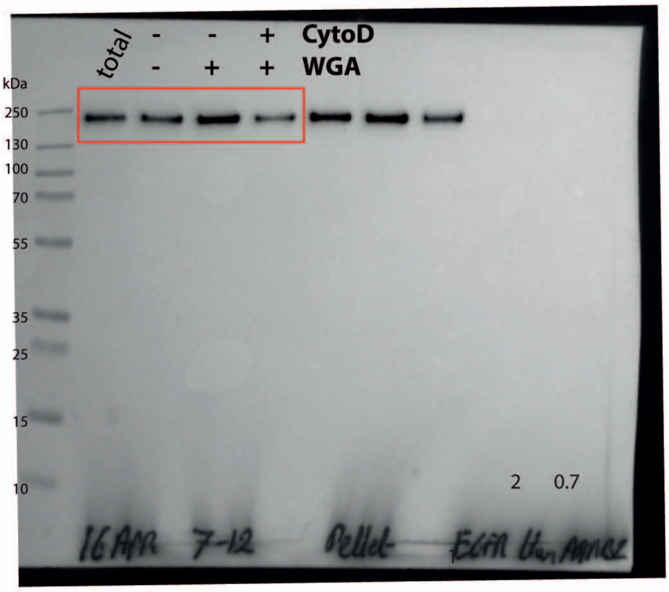

Figure 5a 2nd part

TfR

Pellet

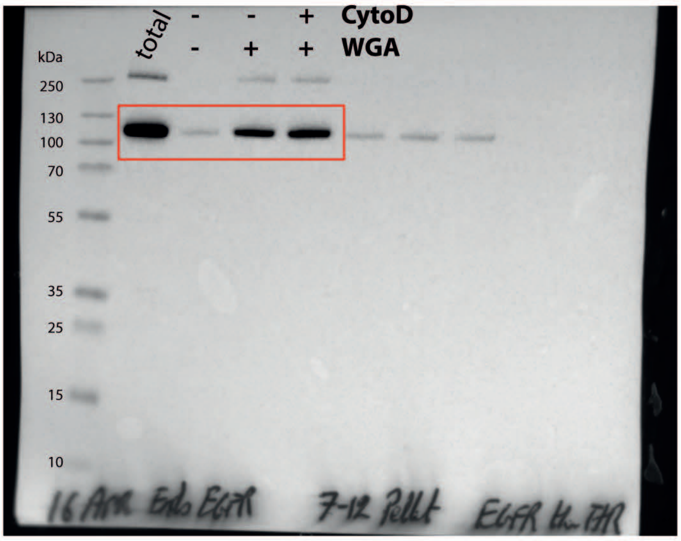

Uptake

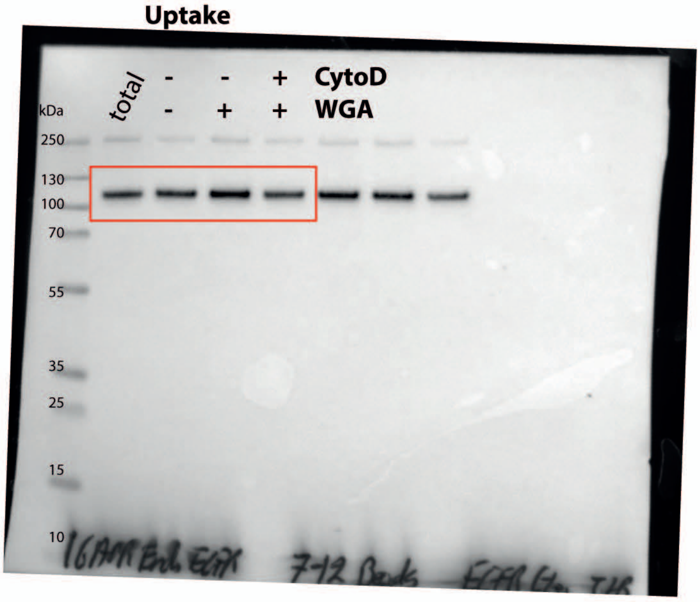

Na/K ATPase

Pellet

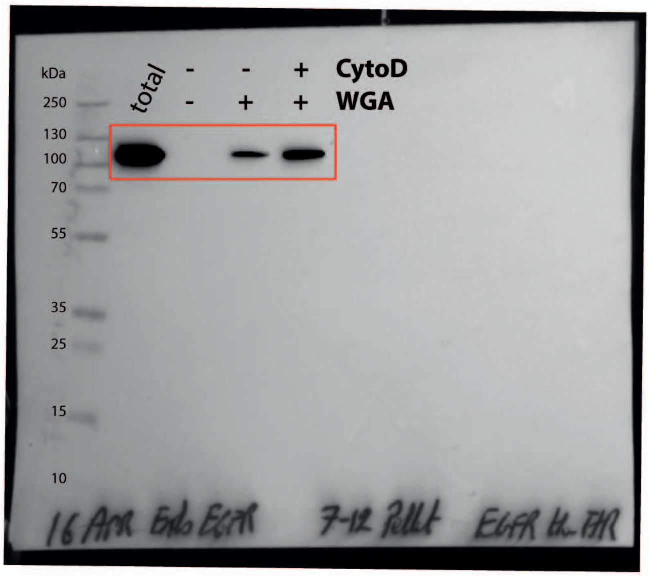

Uptake

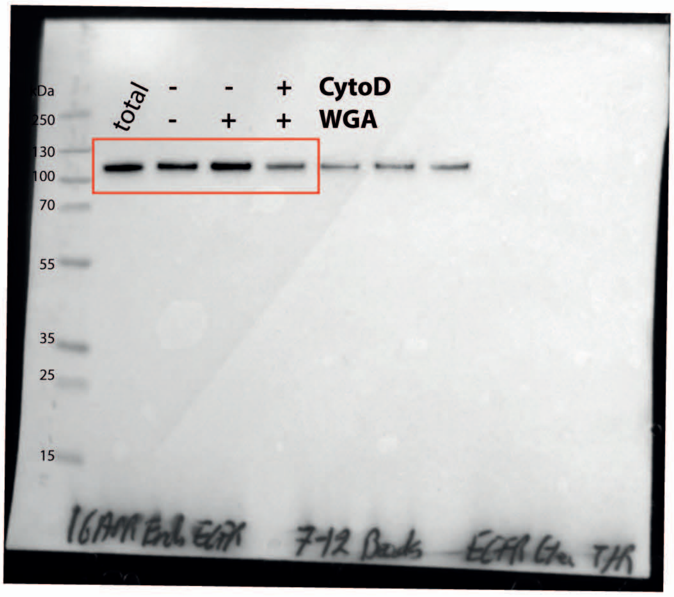

Figure 6a

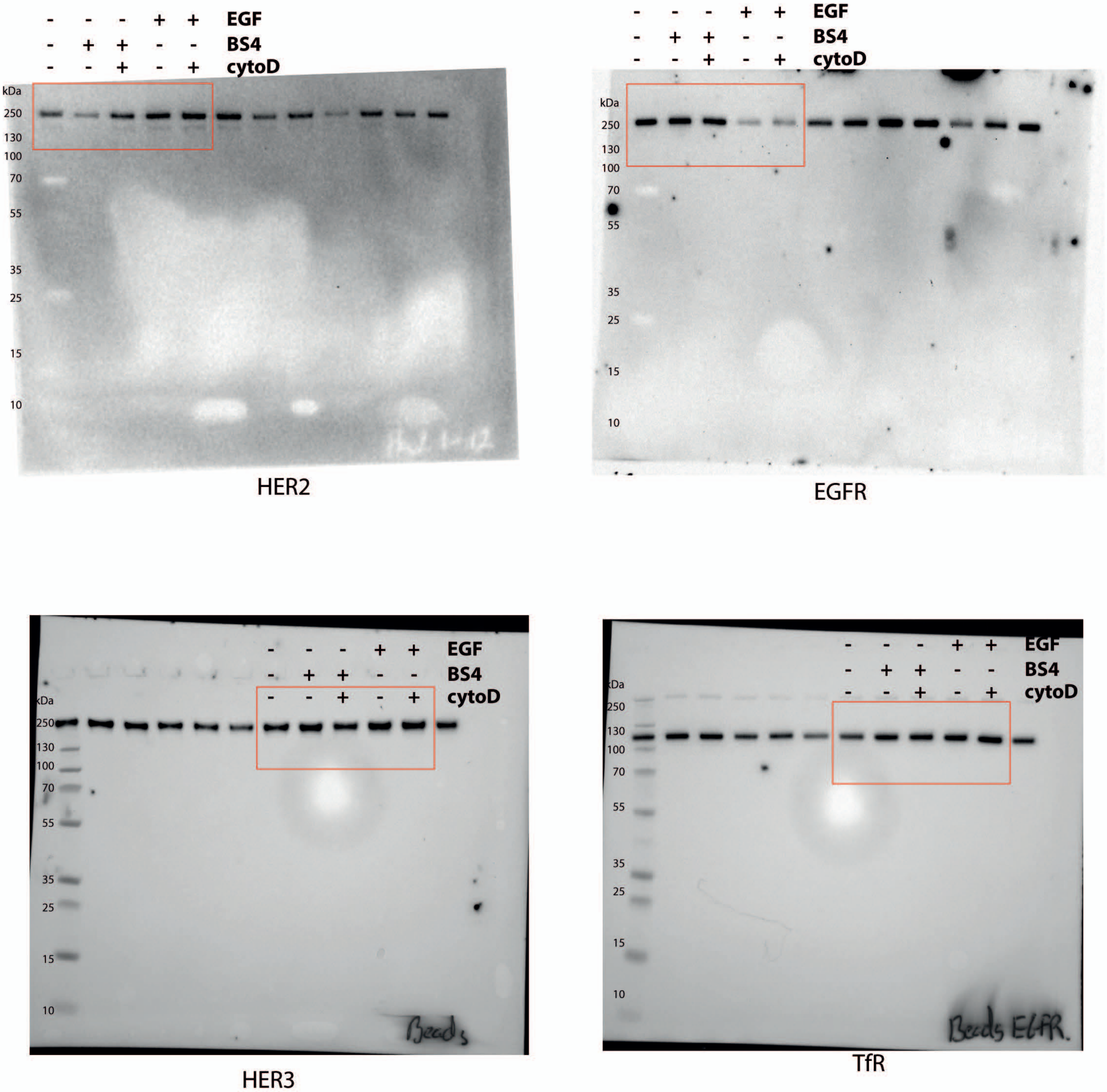
EGF
BS4
cytoD
kDa
250
130
100
70
55
35
25
15
10
HER2
EGFR
HER3
TfR
Beads
Beads EGFR

Figure 6e

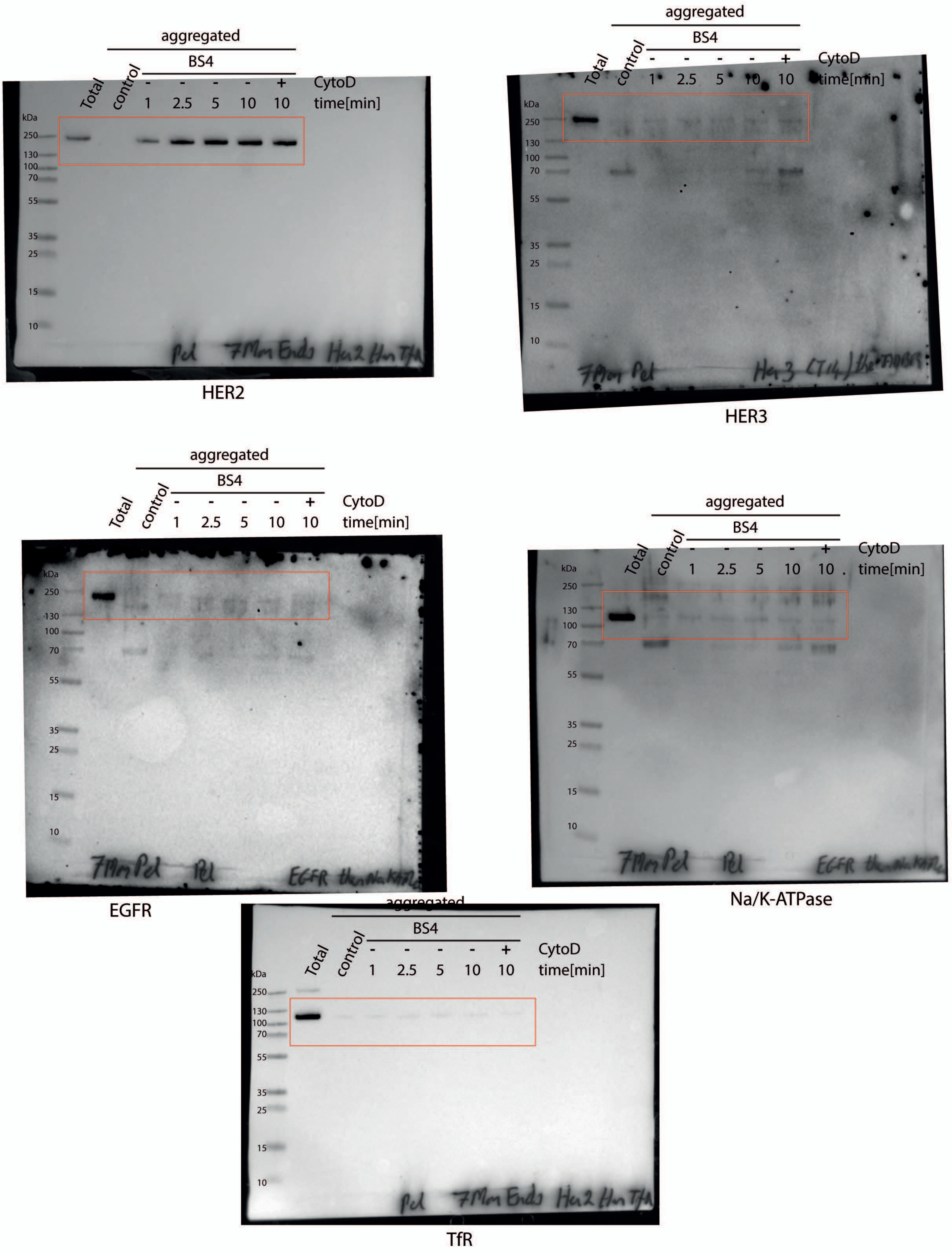
aggregated
BS4
Total
control
- - - - +
CytoD
1 2.5 5 10 10
time[min]
kDa
250
130
100
70
55
35
25
15
10
HER2
HER3
EGFR
Na/K-ATPase
TfR

Figure 6f

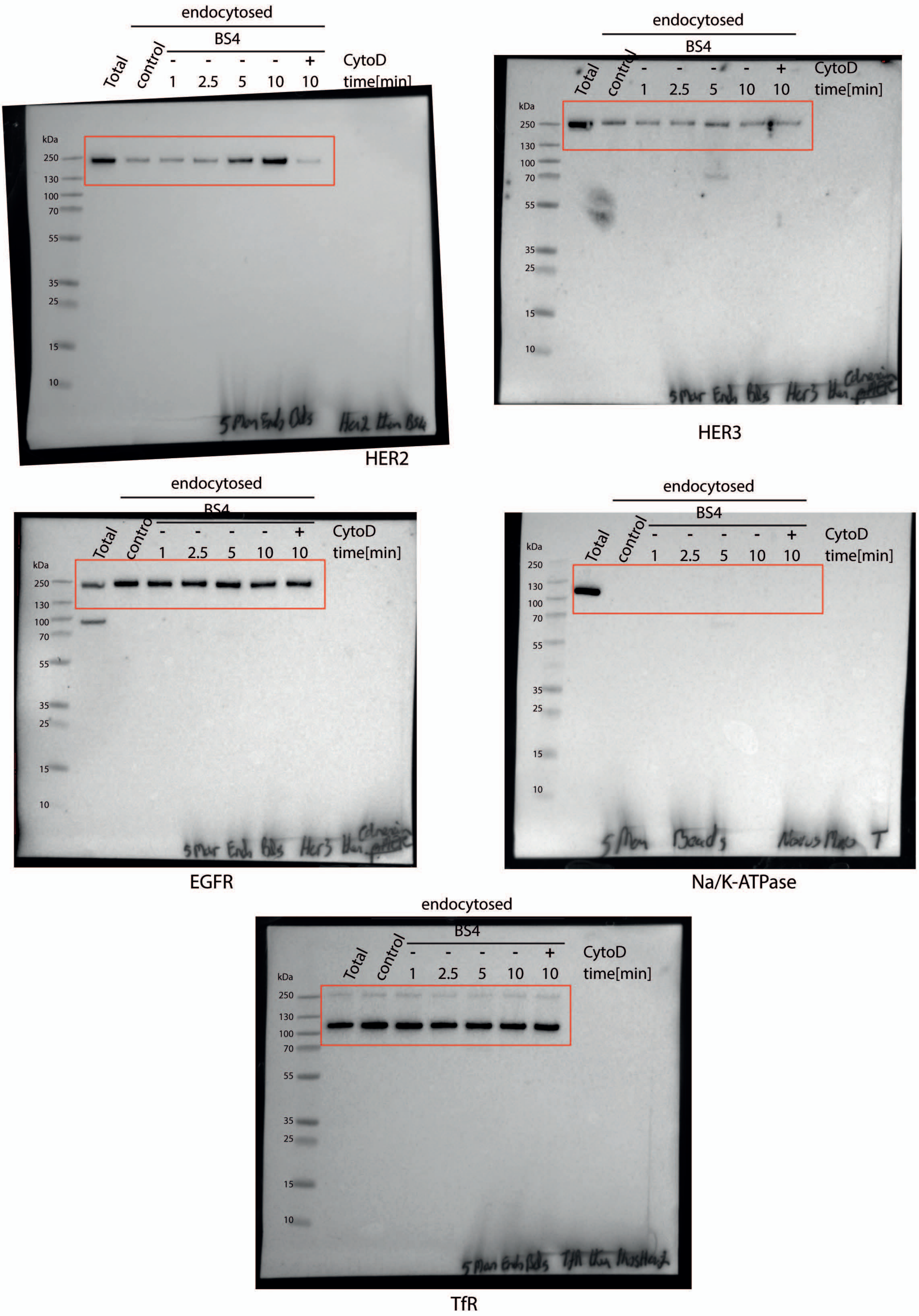
endocytosed
BS4
Total
control
- - - - +
CytoD
1 2.5 5 10 10
time[min]
kDa
250
130
100
70
55
35
25
15
10
HER2
HER3
EGFR
Na/K-ATPase
TfR

Figure 7a

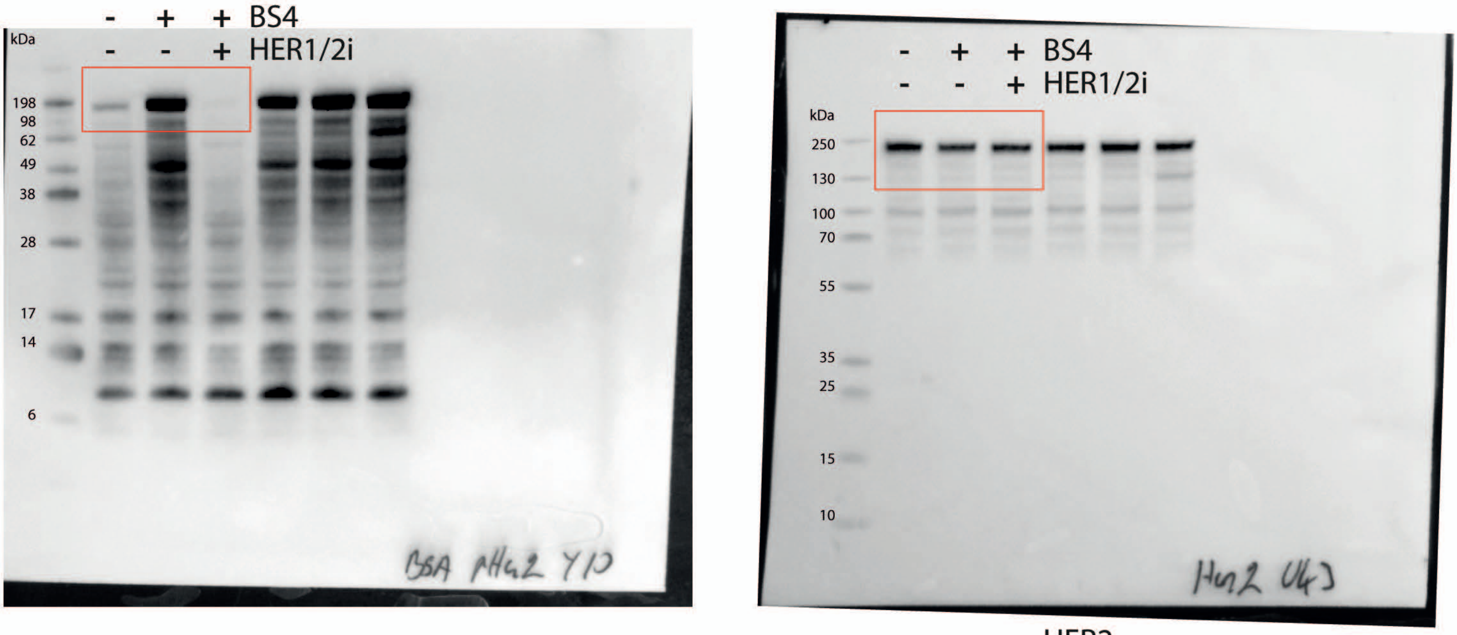

pHER2

HER2

Figure 8a

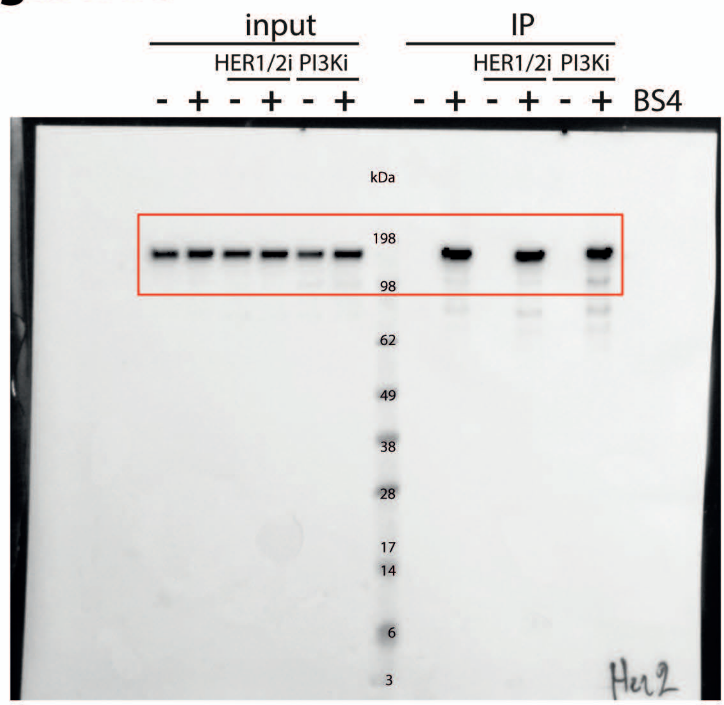

HER2

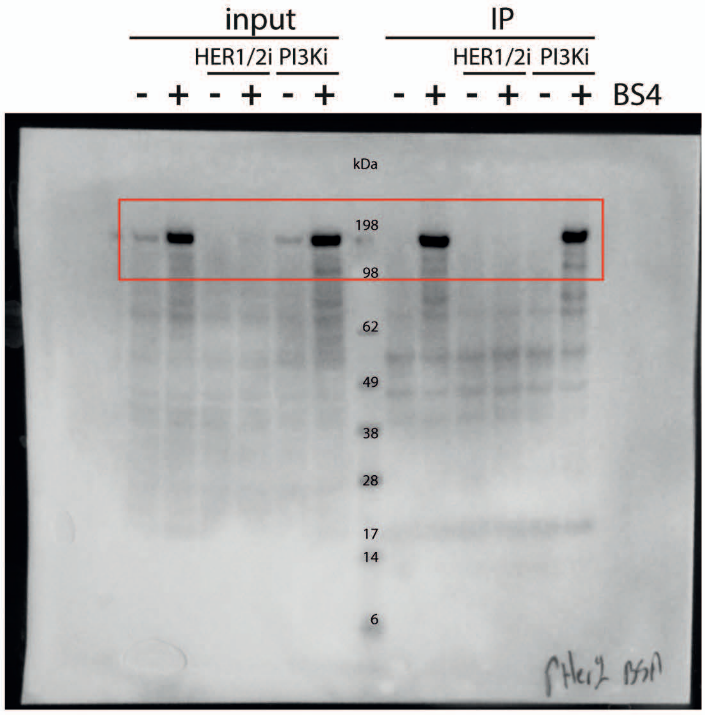

p-Y

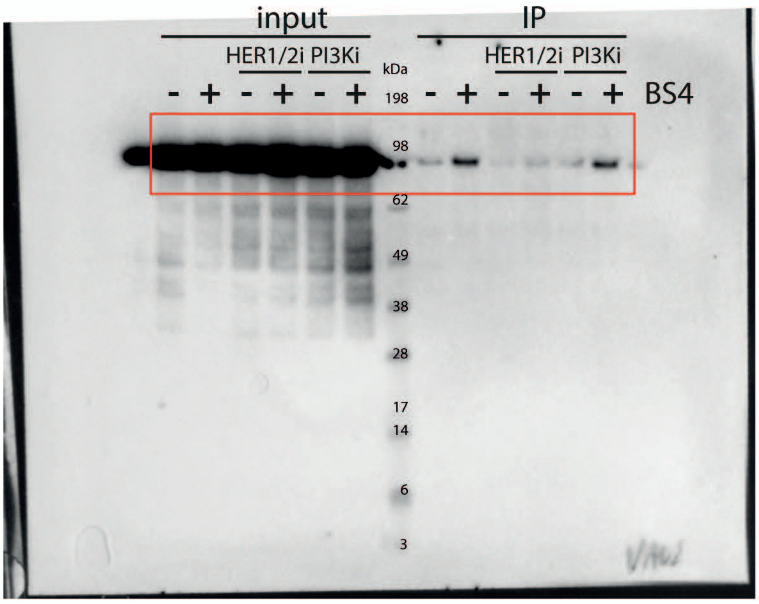

VAV2

Figure 9a

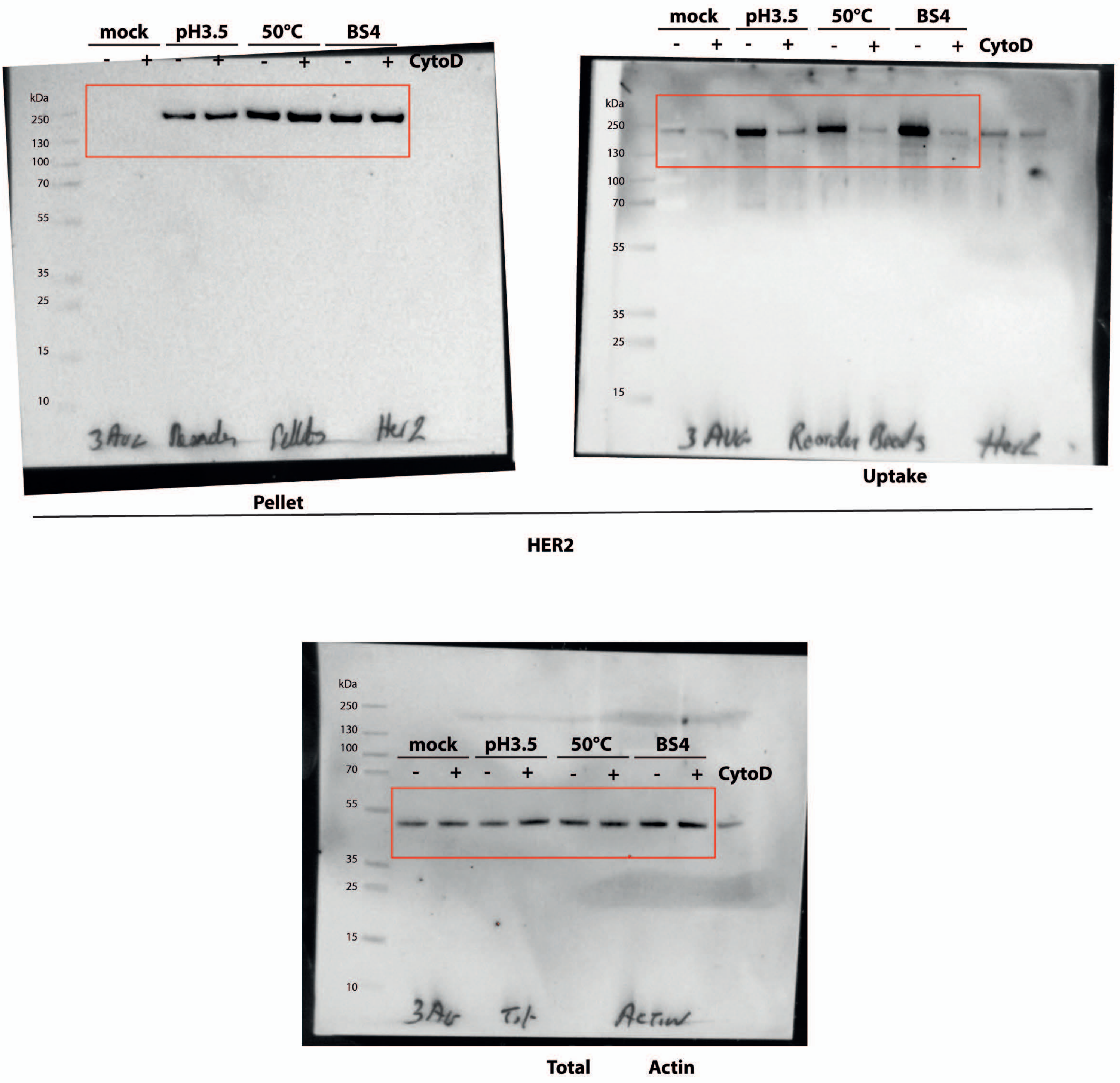
mock
pH3.5
50°C
BS4
- + - + - + - +
CytoD
kDa
250
130
100
70
55
35
25
15
10
Pellet
Uptake
HER2
Total
Actin

Figure 10a

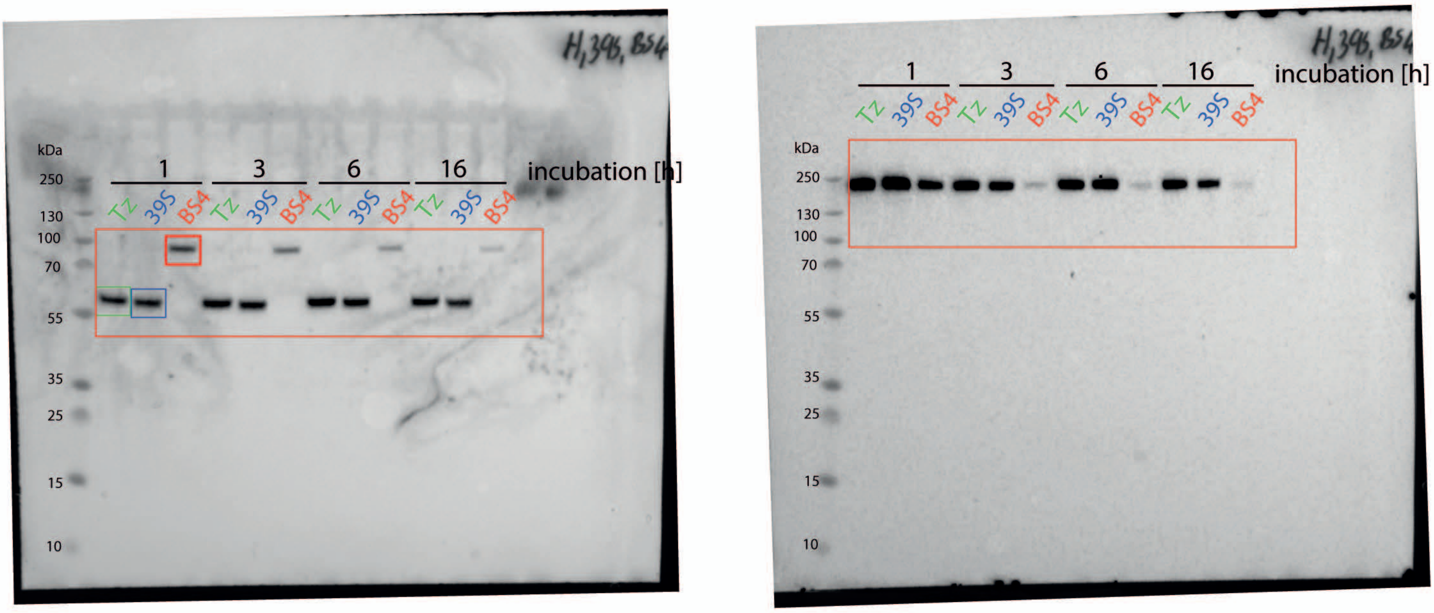

ab
(heavy chain)

HER2

Figure 10b

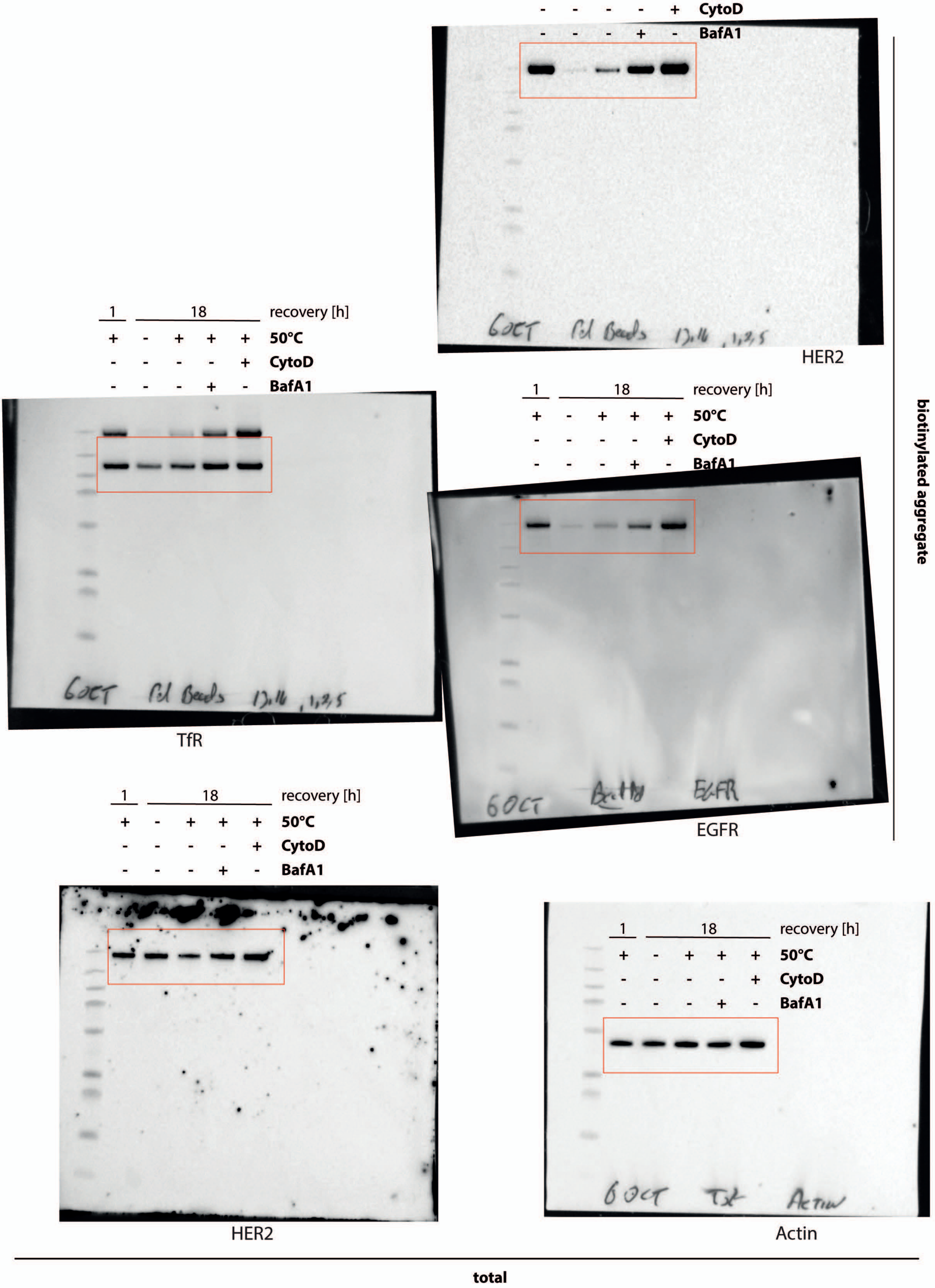
1
18
recovery [h]
50°C
CytoD
BafA1
HER2
TfR
EGFR
HER2
Actin
biotinylated aggregate
total

Figure S4a

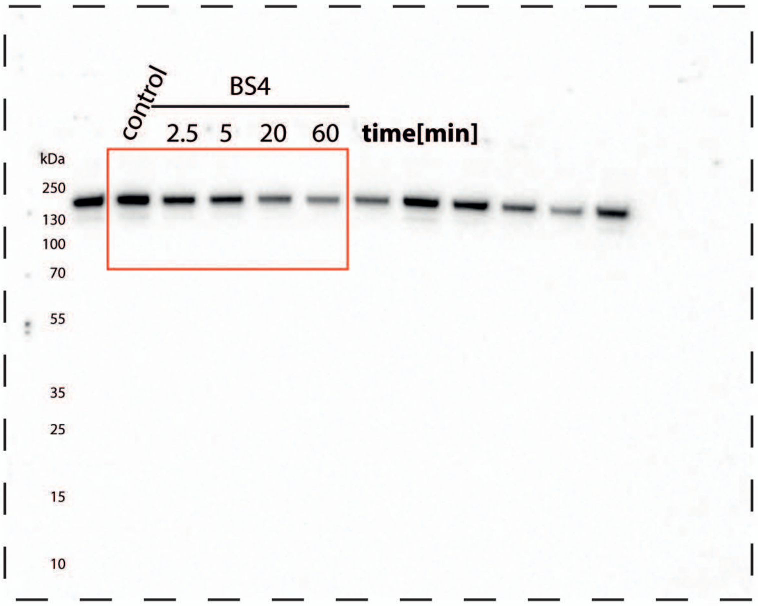

Soluble

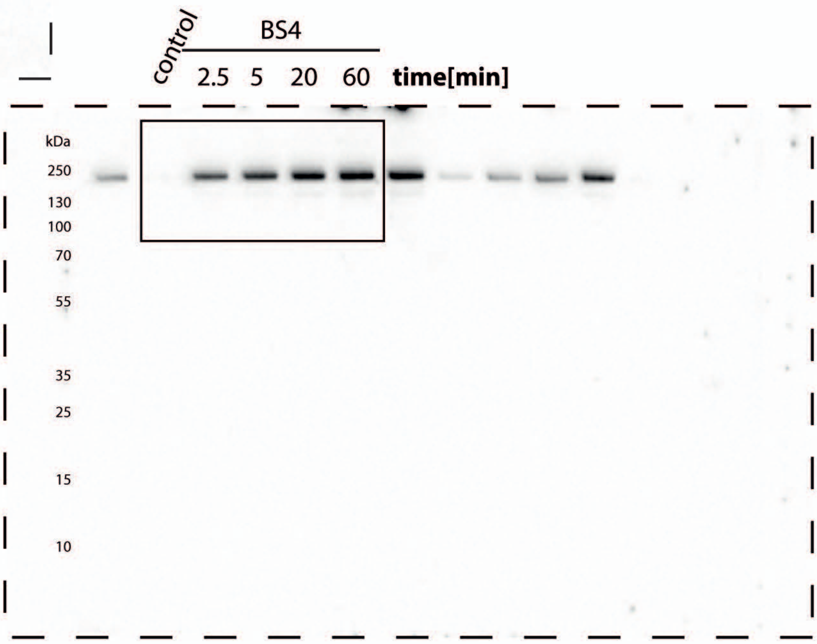

Insoluble
(aggregate in pellet)

Figure S4e

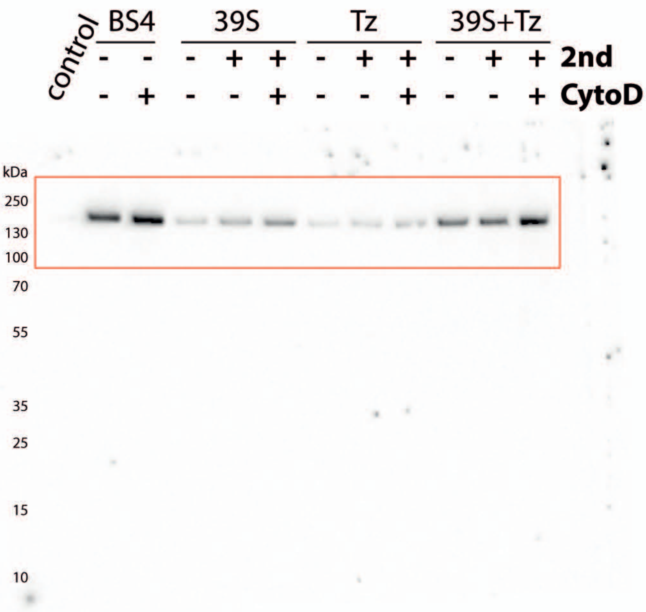

Pellet

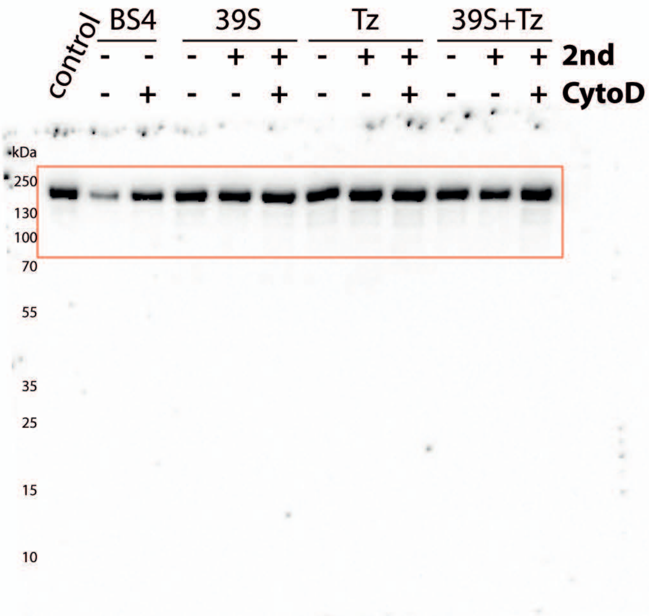

Surface

Her2

Figure S4f

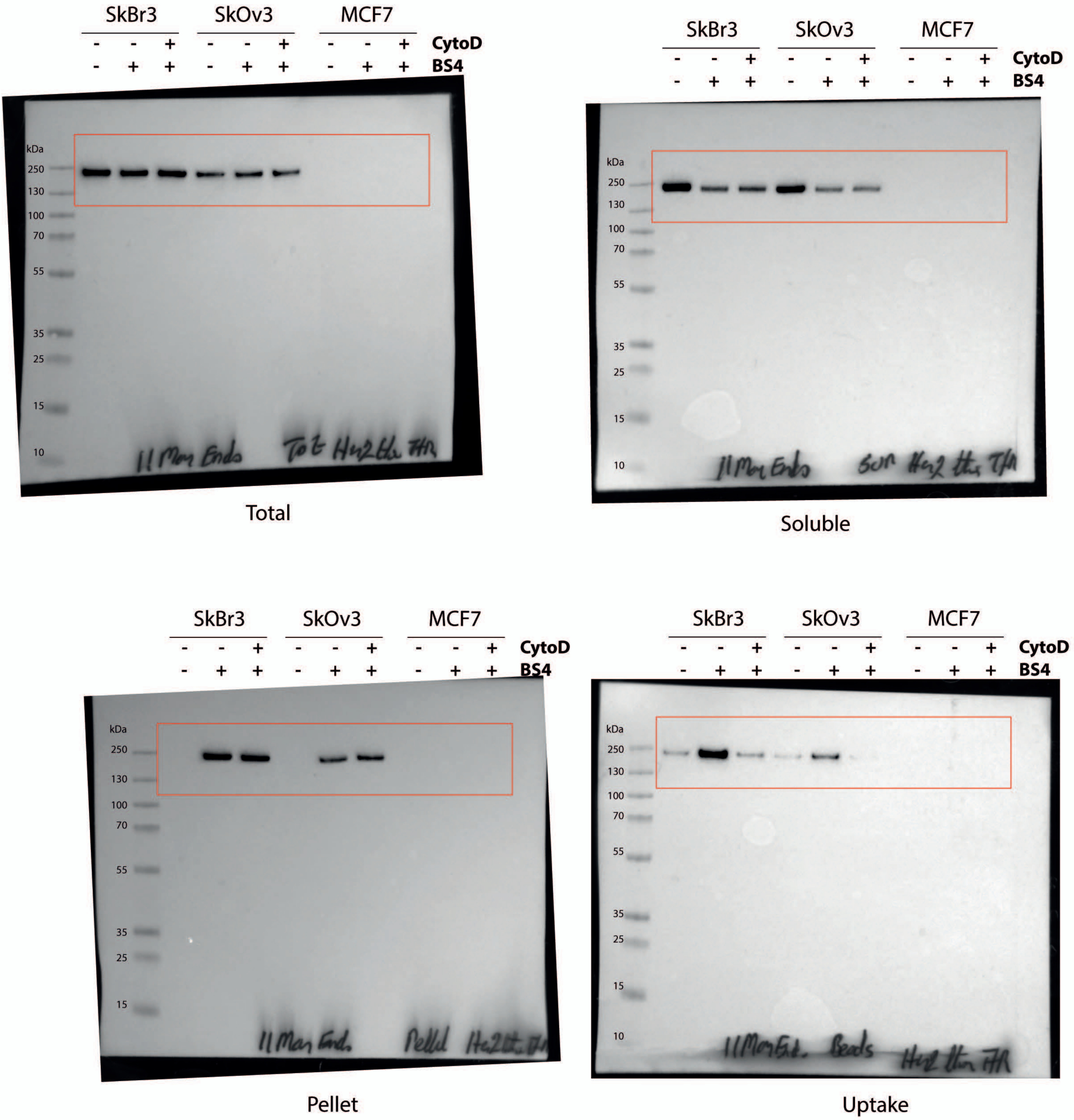

HER2

Figure S4f (100x exp blots for MCF-7 cells)

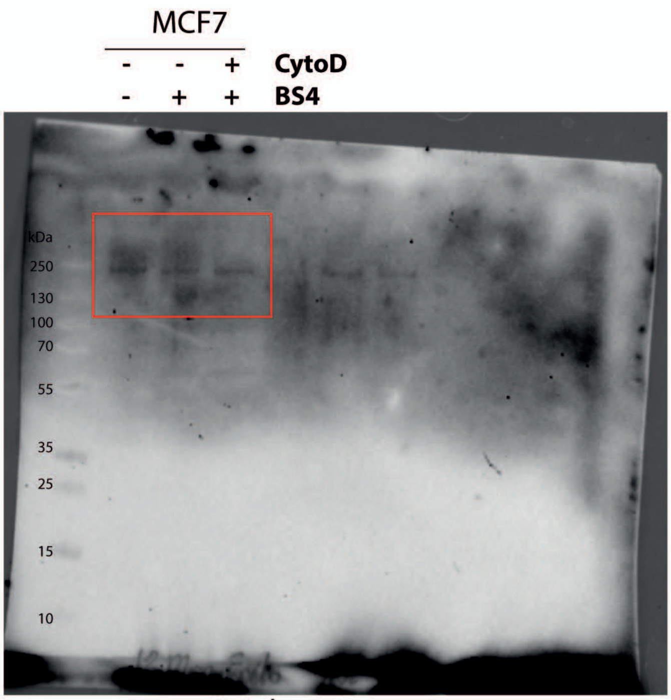

total **100x exp**

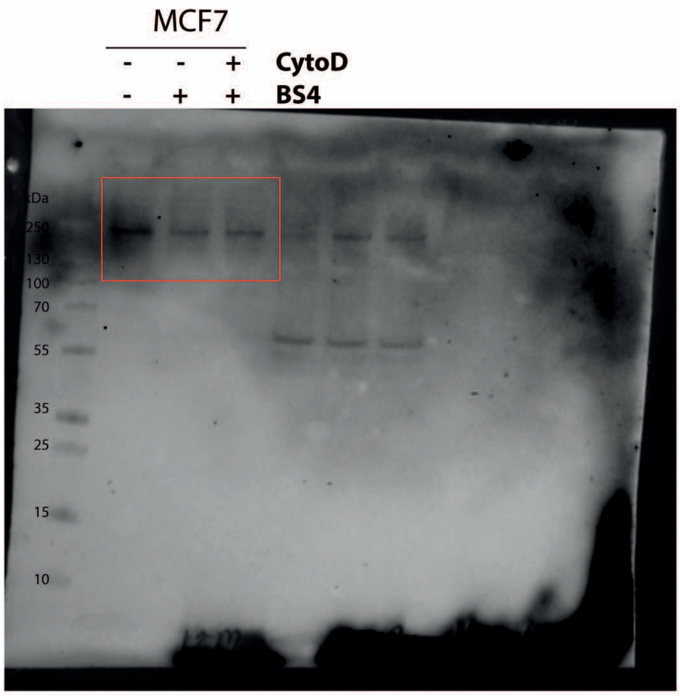

soluble **100x exp**

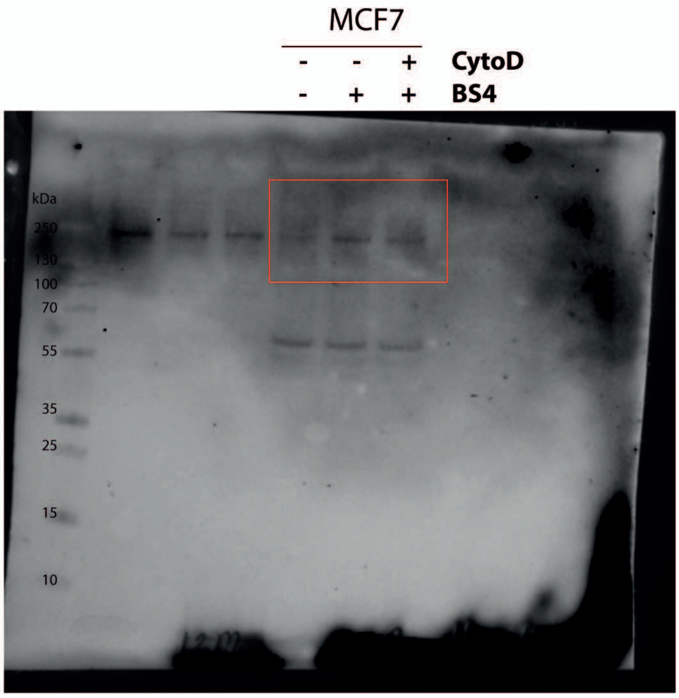

Pellet **100x exp**

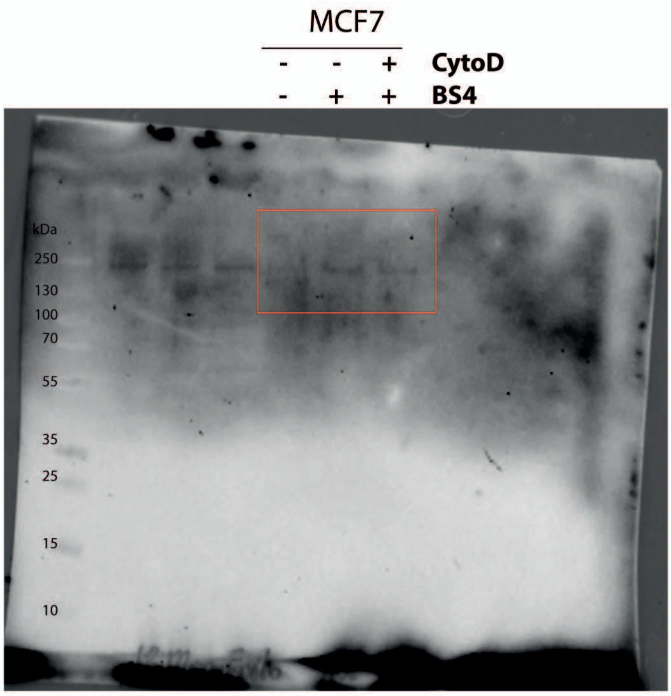

Uptake **100x exp**

HER2

Figure S5a

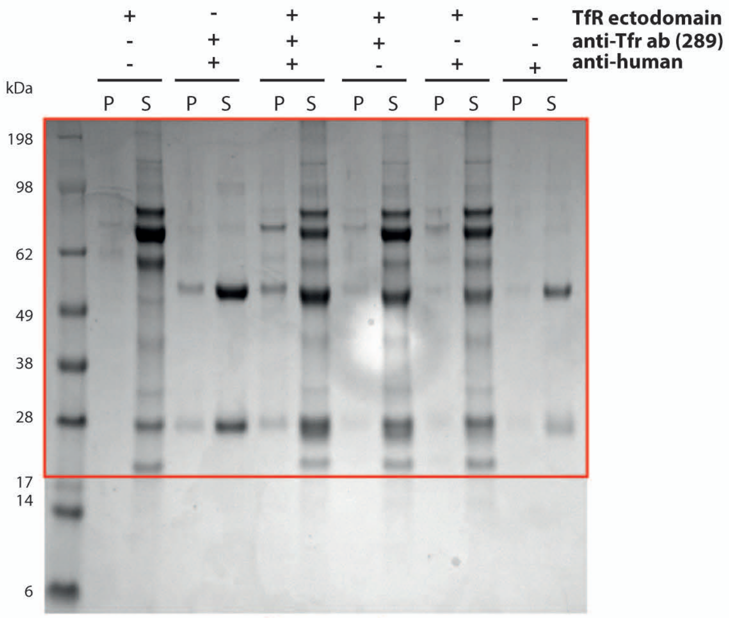

Coomassie

Figure S6b

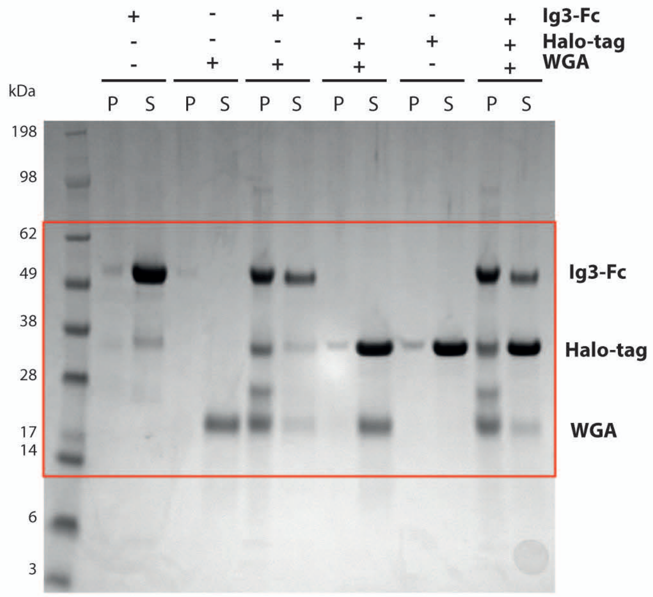

Coomassie

Fig S8a

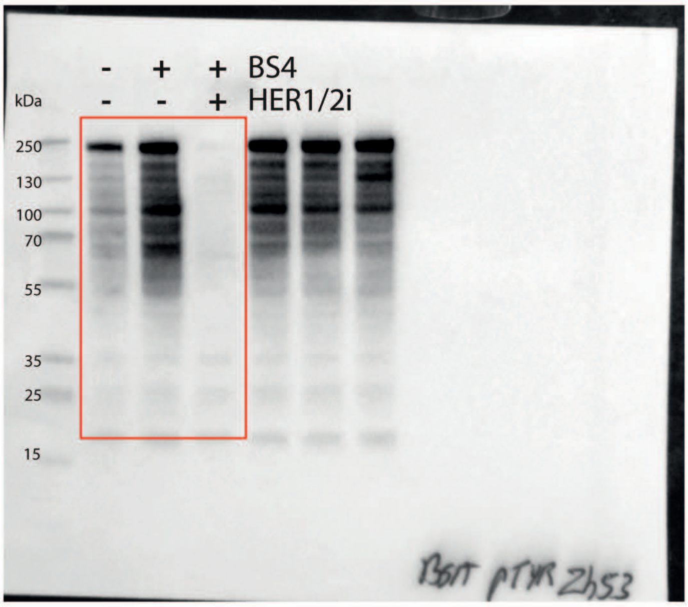

p-tyrosine

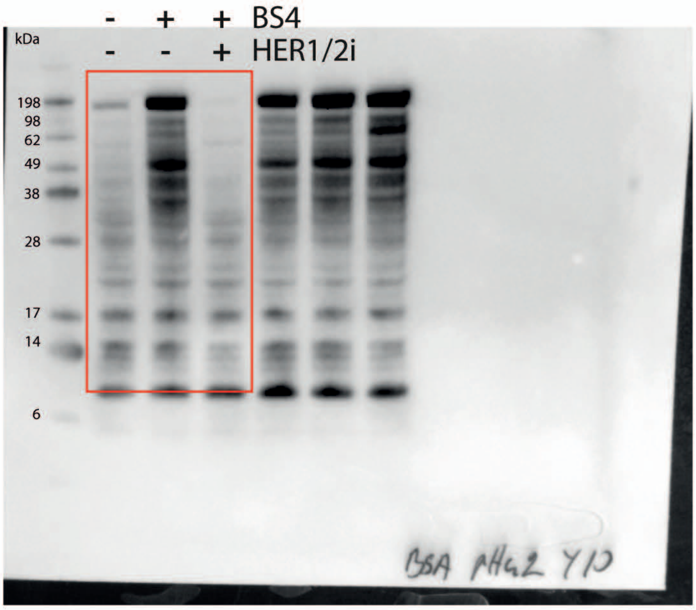

pHER2

Figure S9d

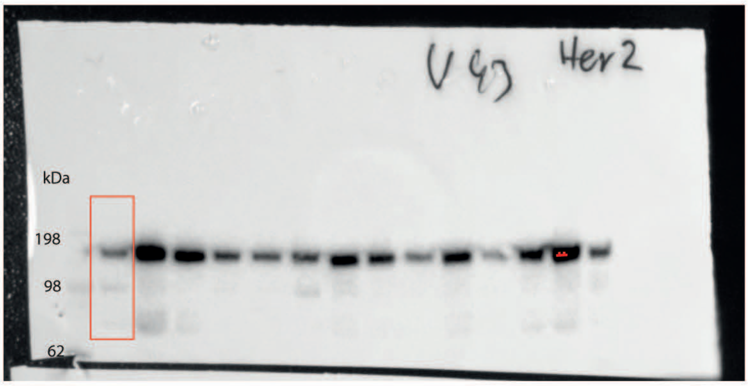

HER2

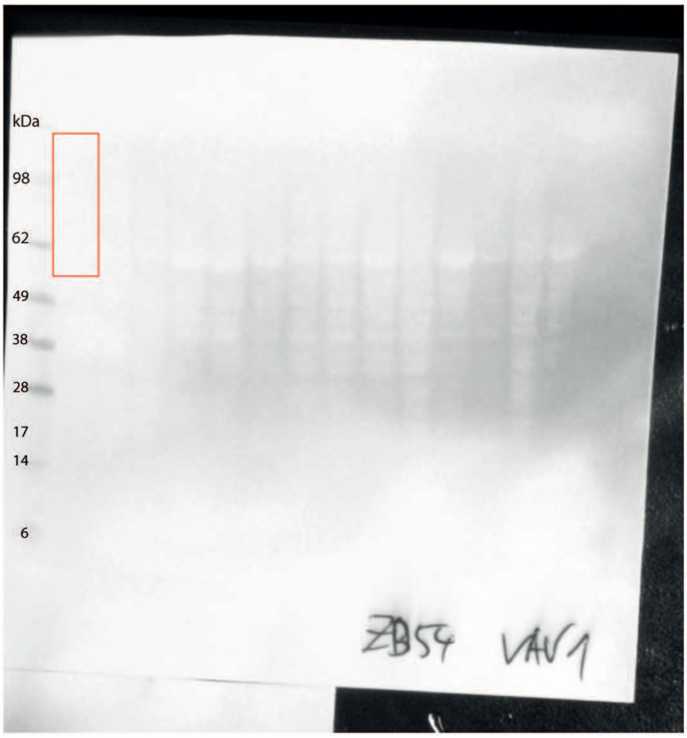

VAV1

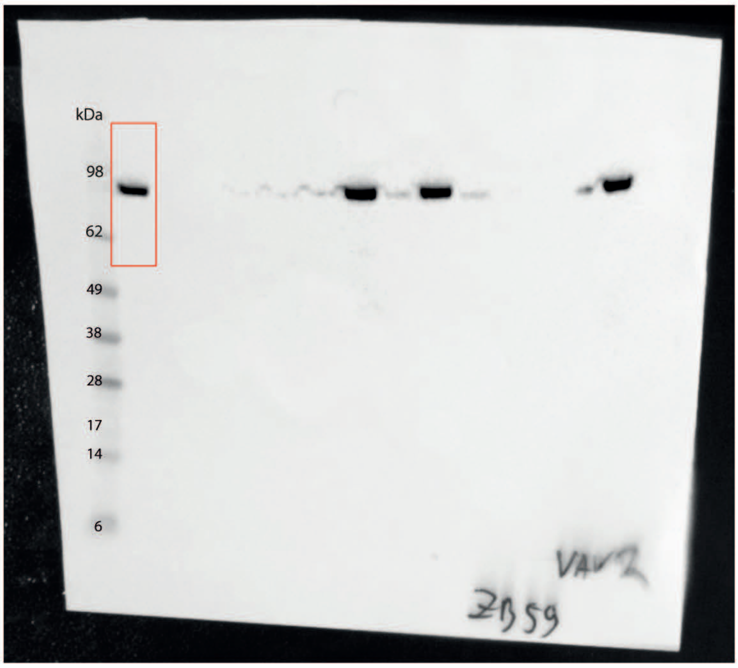

VAV2

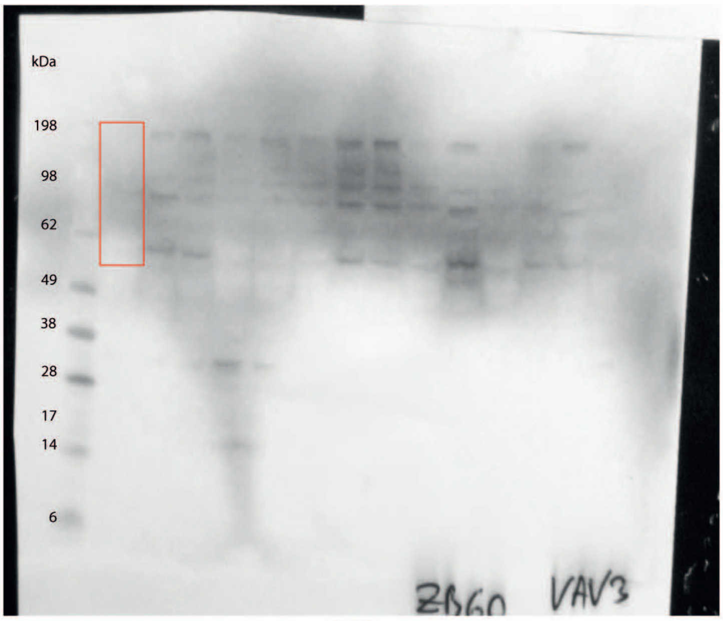

VAV3

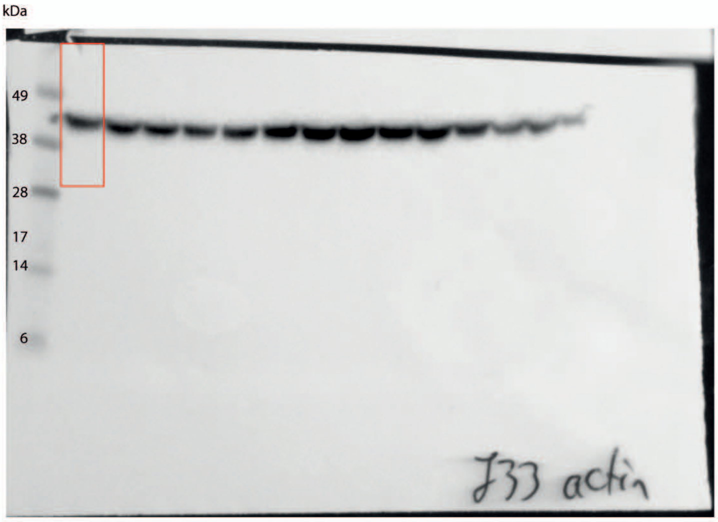

actin

Figure S9f

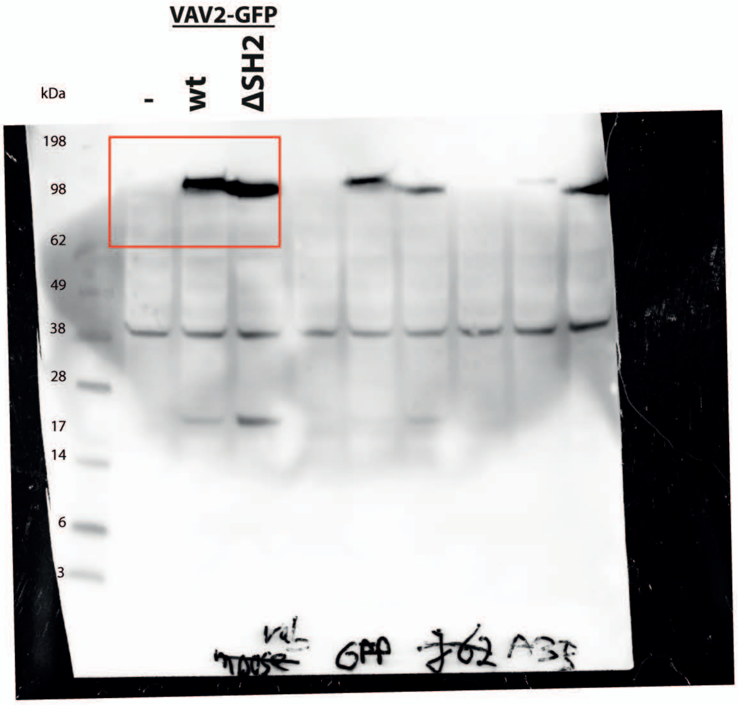

GFP

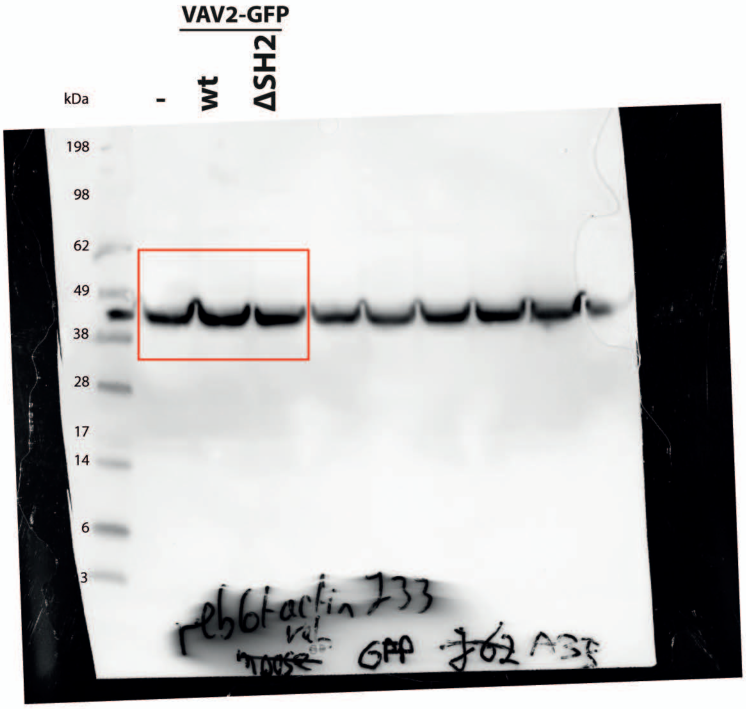

actin

Figure S10a

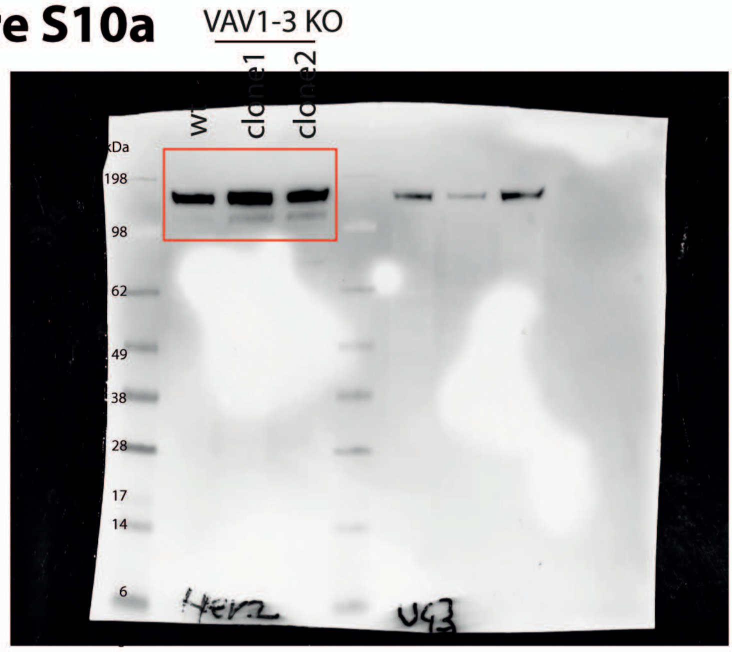

HER2

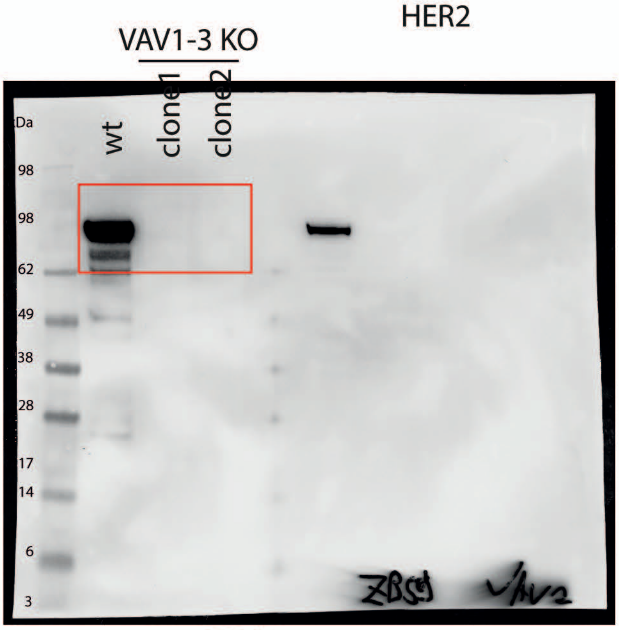

VAV2

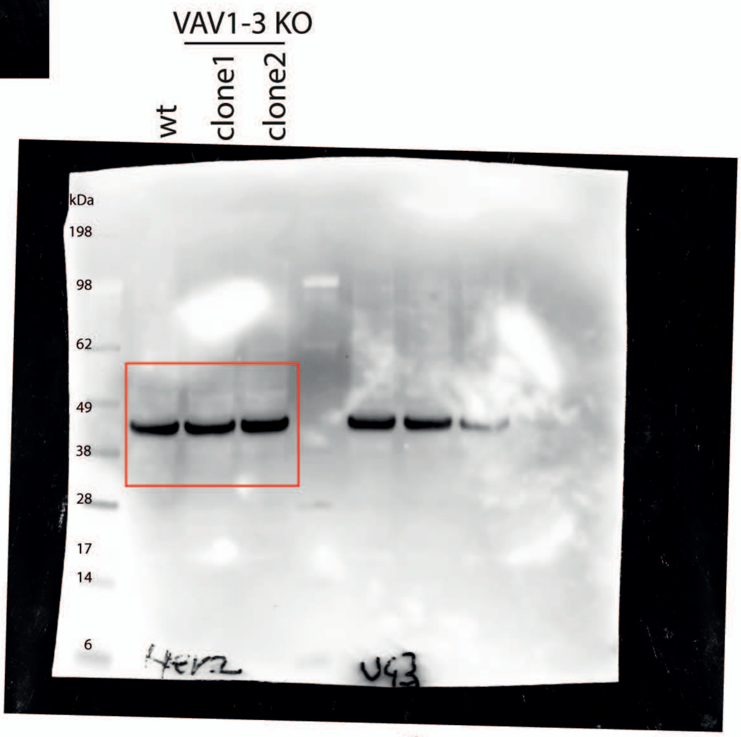

actin

Figure S10c

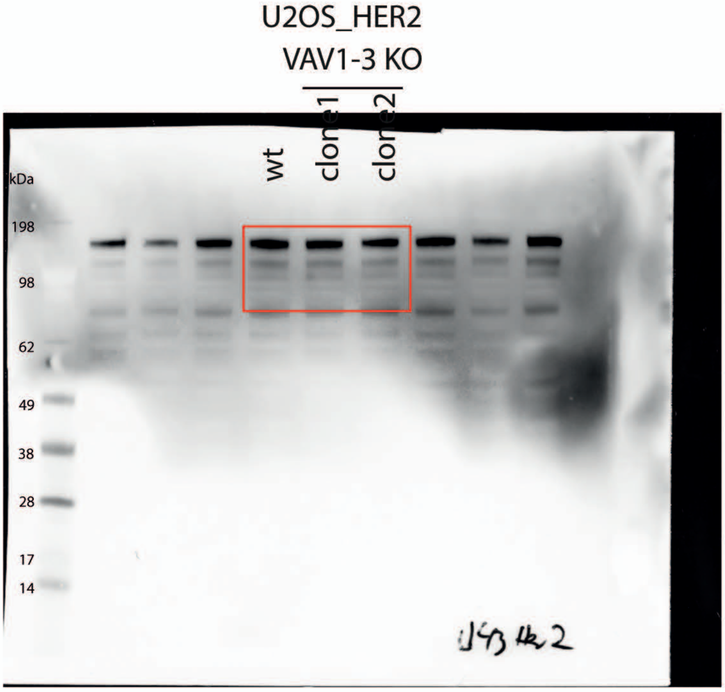

HER2

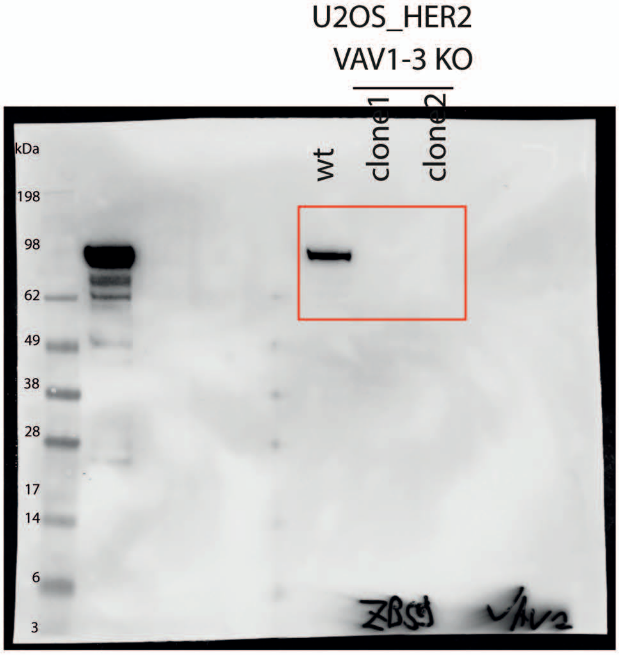

VAV2

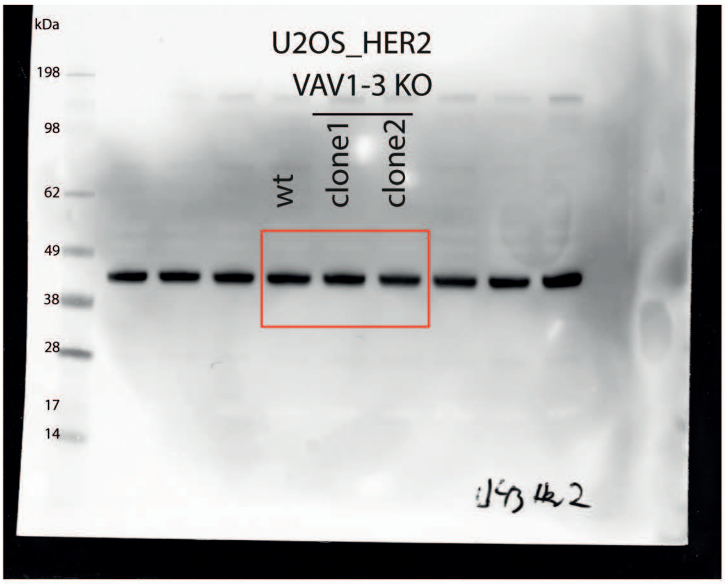

actin

Figure S10g

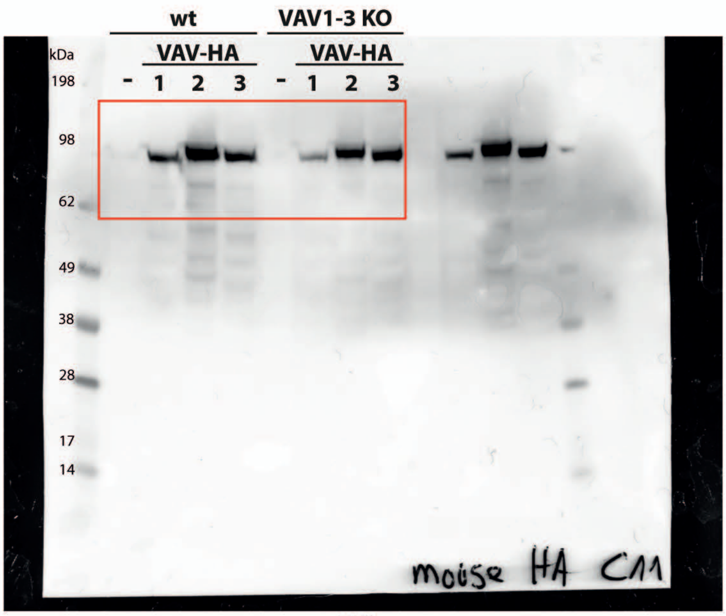

HA

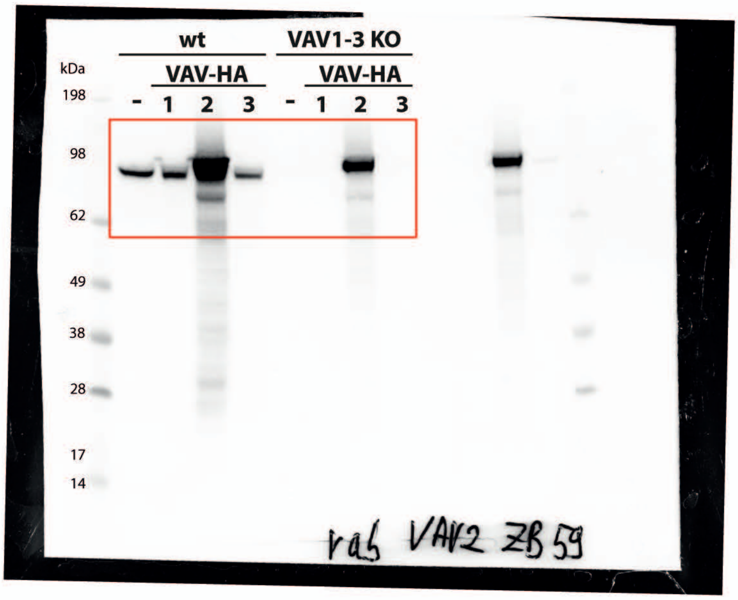

VAV2

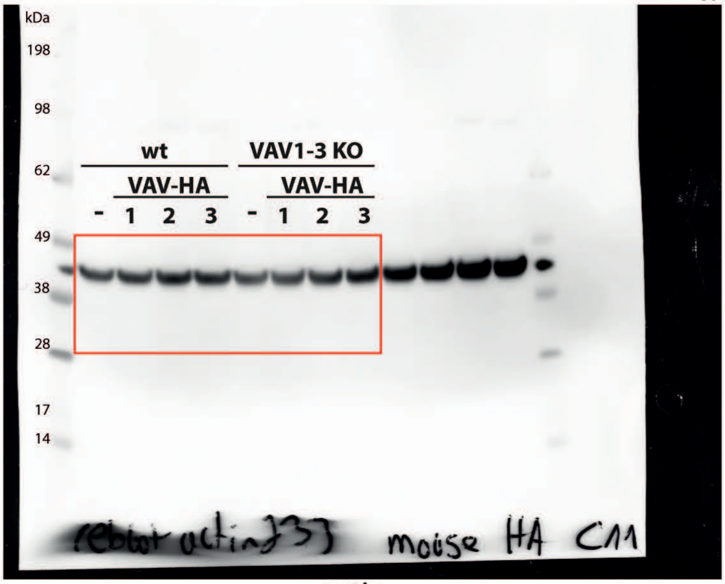

actin

Figure S10h

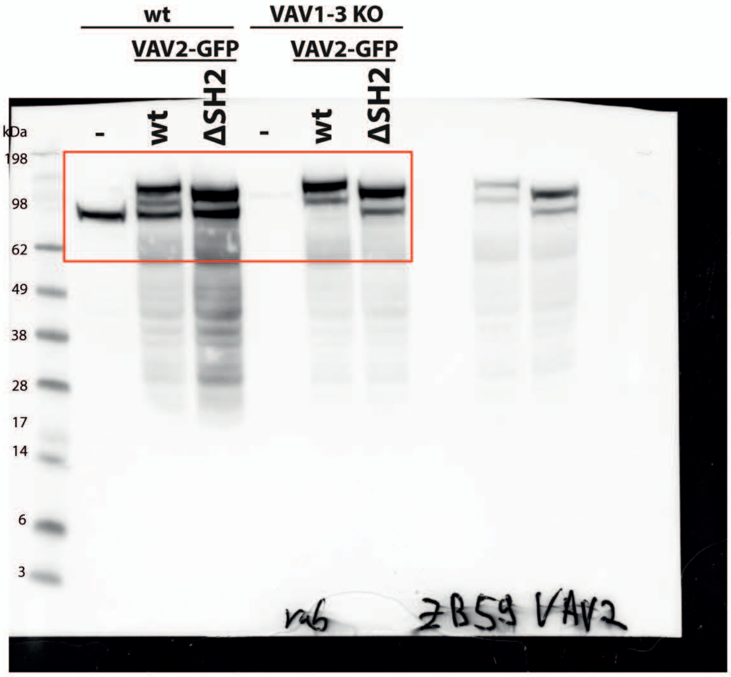

VAV2

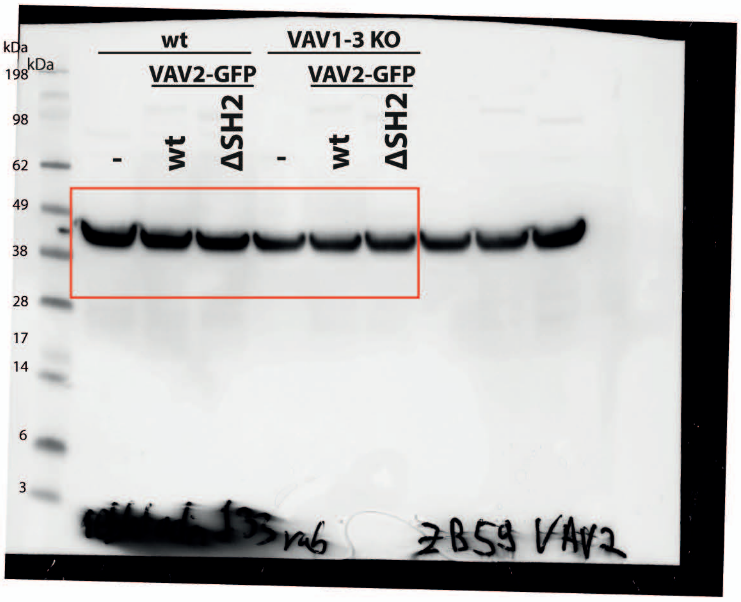

actin

Figure S11a

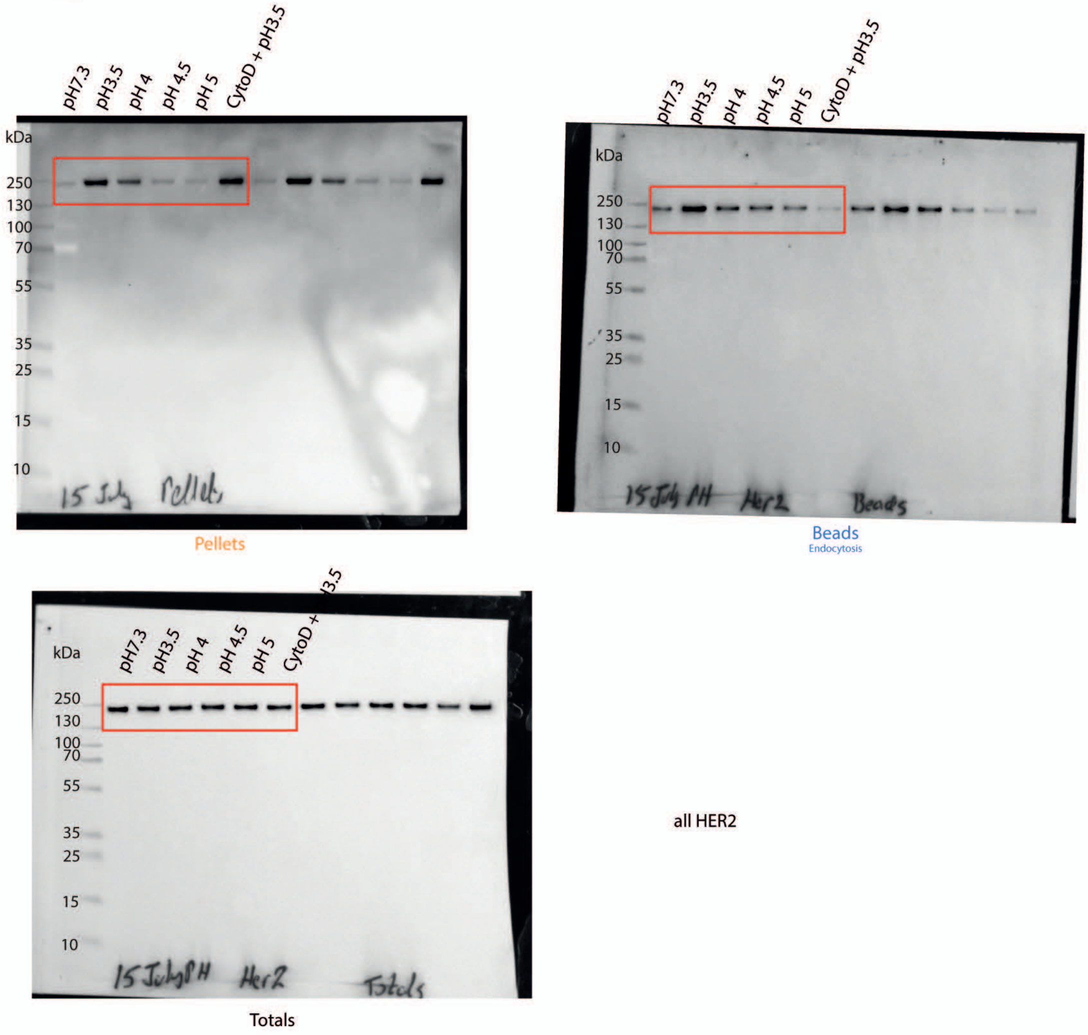

Figure S11b

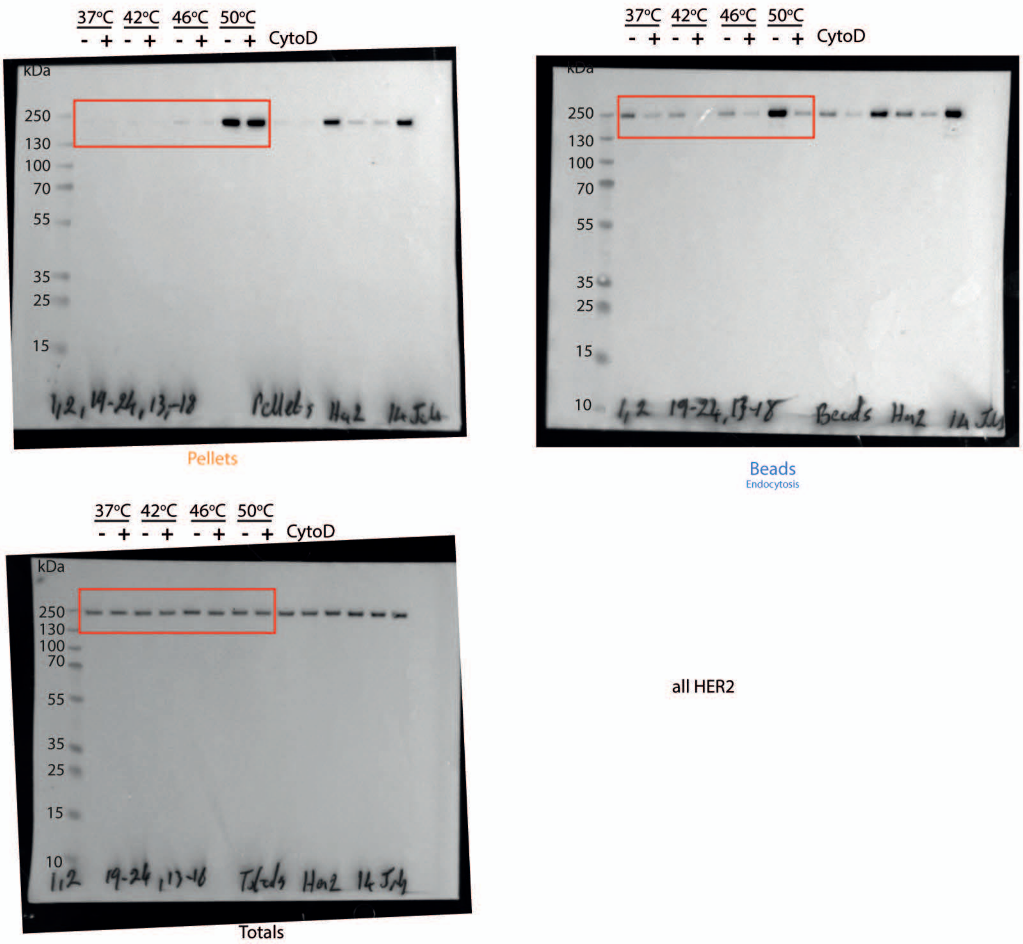

Figure S11e #1

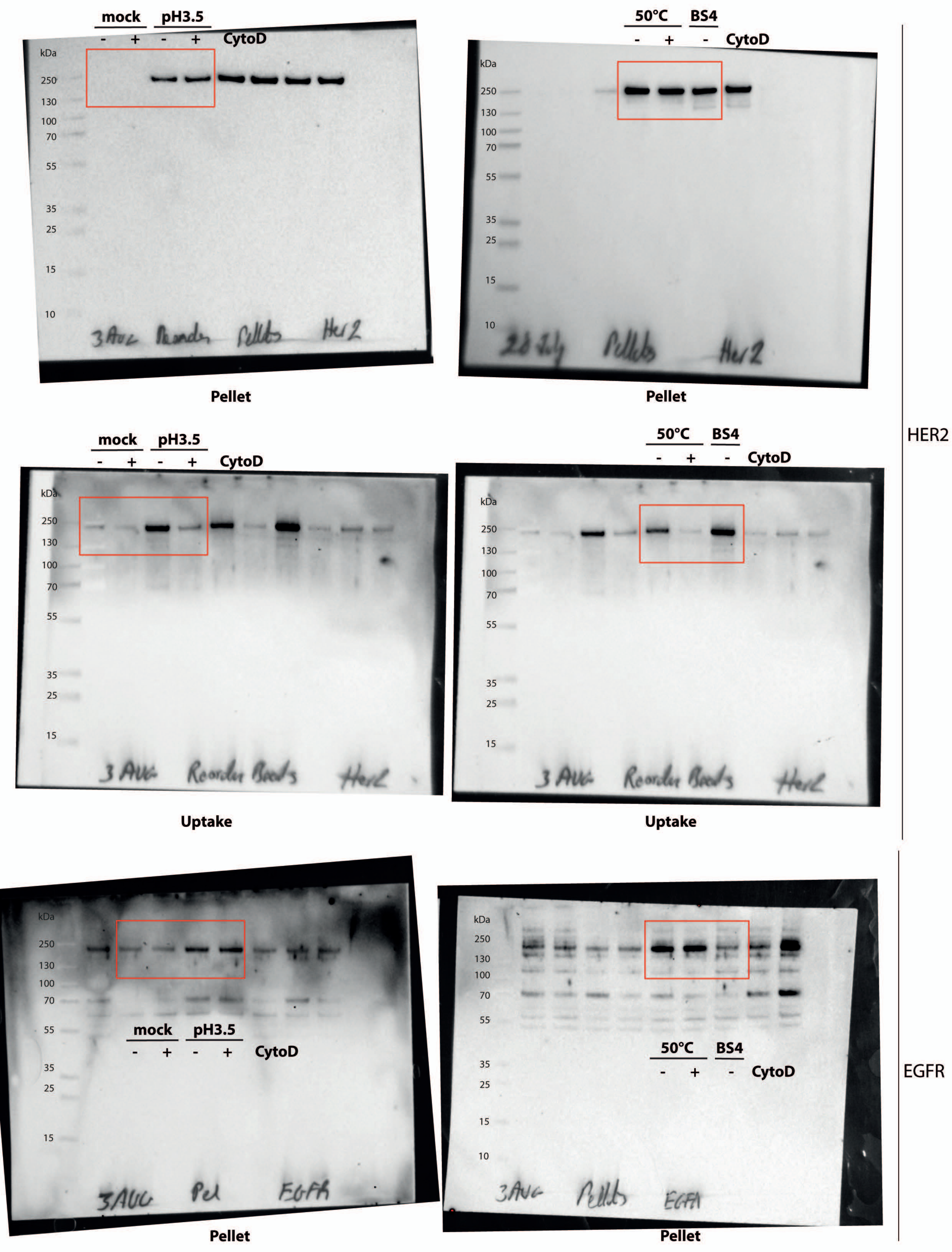

Figure S11e #2

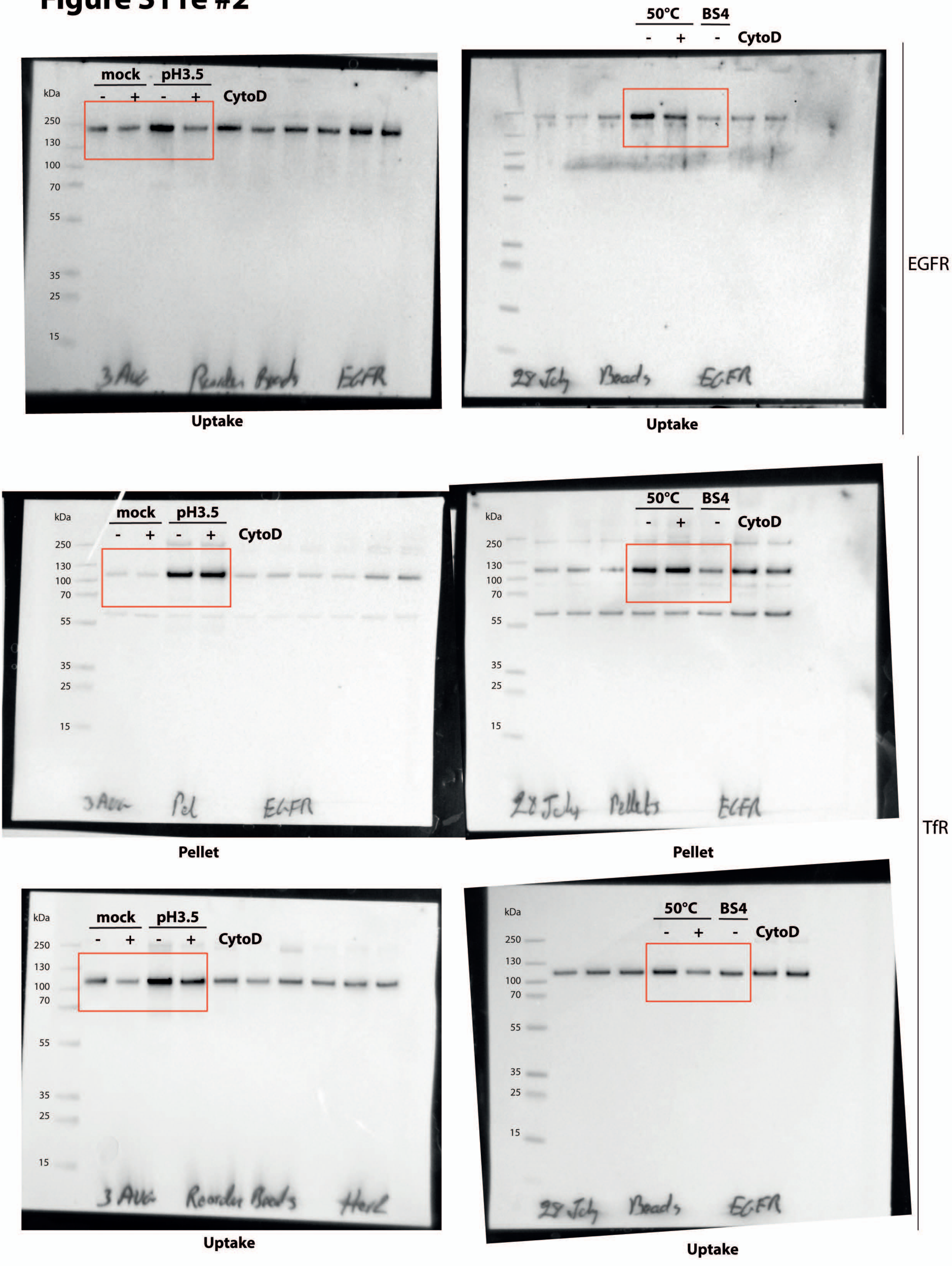

Figure S11e #3

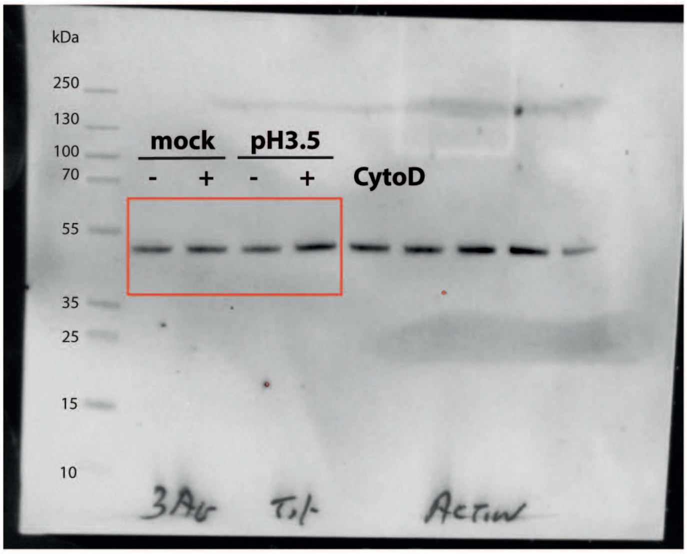

Total

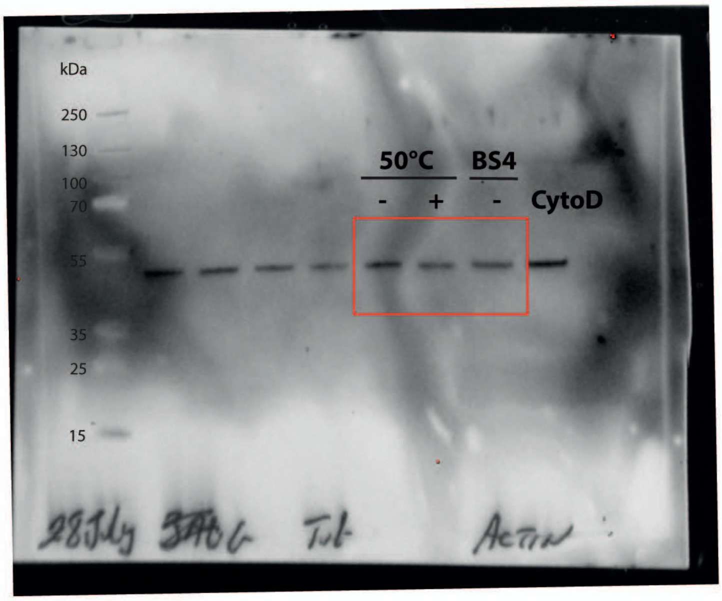

Total

Actin

Figure S13b

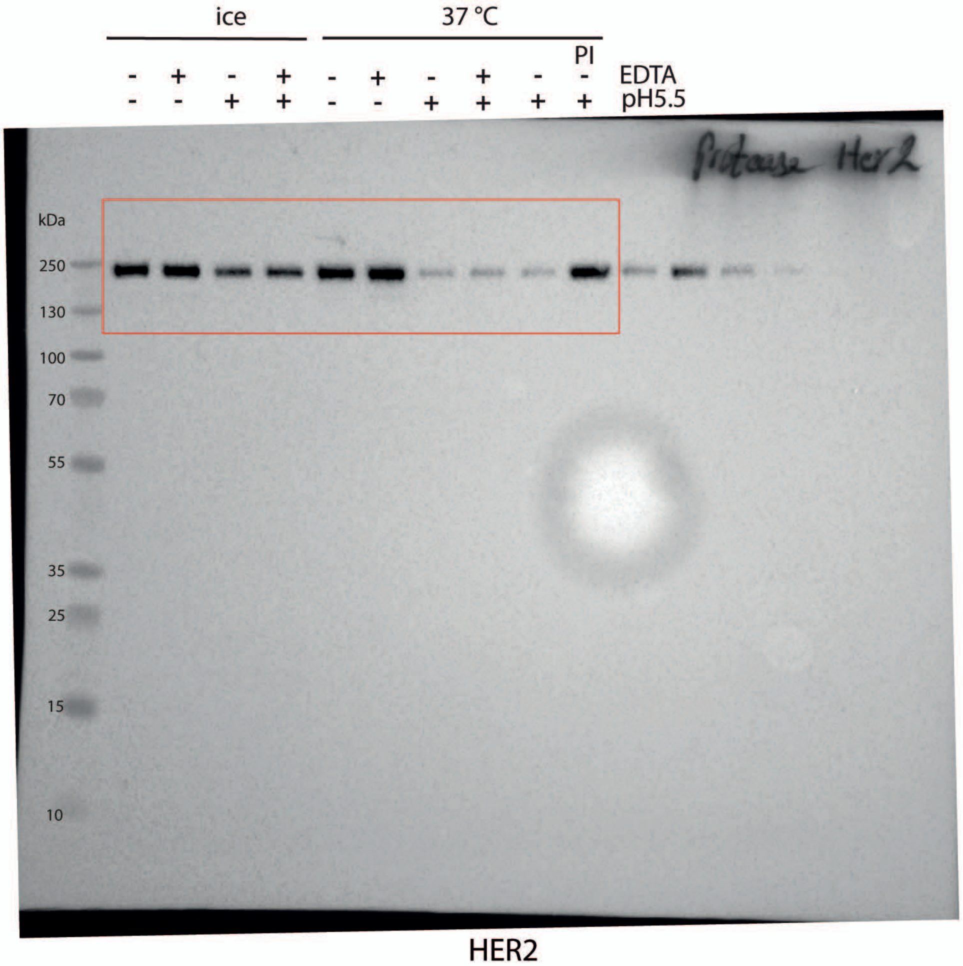

HER2

Figure S13c

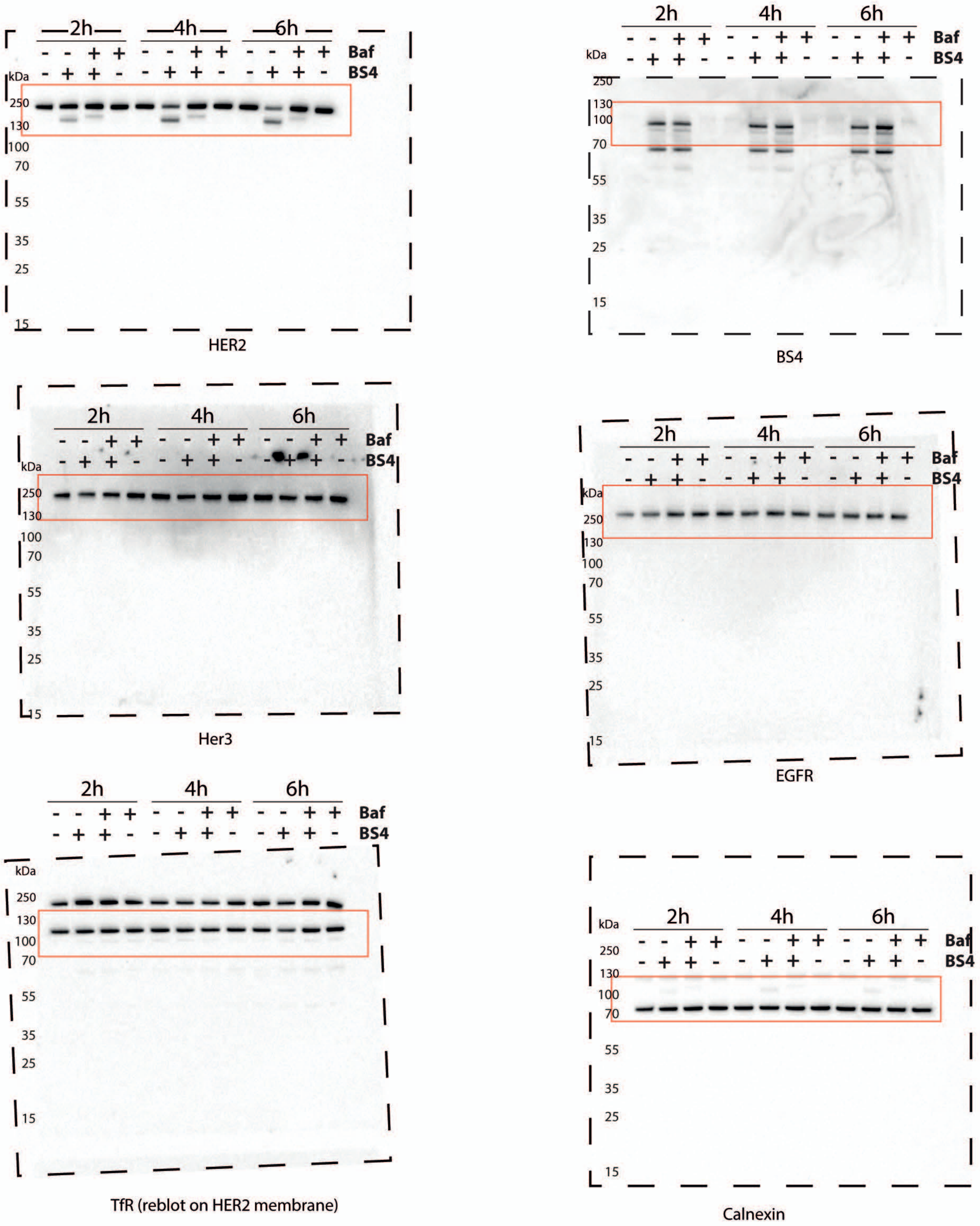
2h
4h
6h
Baf
BS4
kDa
250
130
100
70
55
35
25
15
HER2
BS4
Her3
EGFR
TfR (reblot on HER2 membrane)
Calnexin
