# Supplementary material for: Cell surface protein aggregation triggers endocytosis to maintain plasma membrane proteostasis
Source: Nat Commun. 2023 Feb 28;14:947. doi: 10.1038/s41467-023-36496-y (PMC9974993; doi:10.1038/s41467-023-36496-y)
Supplement: Supplementary file 13 — Source Data [file 41467_2023_36496_MOESM13_ESM.zip › Spot_coloc.zip!/Readme file for spot-colocalisation macro.docx]

**Readme file for spot-colocalisation macro to be used in FIJI**

**System requirements:**

FIJI is written in Java, which allows it to run on Linux, Mac OS X and Windows, in both 32-bit and 64-bit modes.

We have run it on FIJI 2.3.0 and 2.9.0 in MacOS 11.7, but we expect it to be compatible with any setup that runs FIJI.

No non-standard hardware is required.

**Installation guide:**

Open FIJI and open the macro via File>Open>Spot_Coloc_Paul (selecting the file in whatever folder it is saved).

Installation time less than 1 minute.

**Demo, instructions for use:**

Open FIJI and macro as described above. Open dual channel image to be analyzed. Click “Run” and select parameters as desired (analysis parameters have to be kept constant between different experimental groups). Expected runtime depends on the settings and resulting objects, but typically is in the min range per image.
